# Supplementary figures and images for: Intraflagellar transport protein IFT172 contains a C-terminal ubiquitin-binding U-box-like domain involved in ciliary signaling
Source: eLife. 2026 Jun 23;14:RP104906. doi: 10.7554/eLife.104906 (PMC13290226; doi:10.7554/eLife.104906)

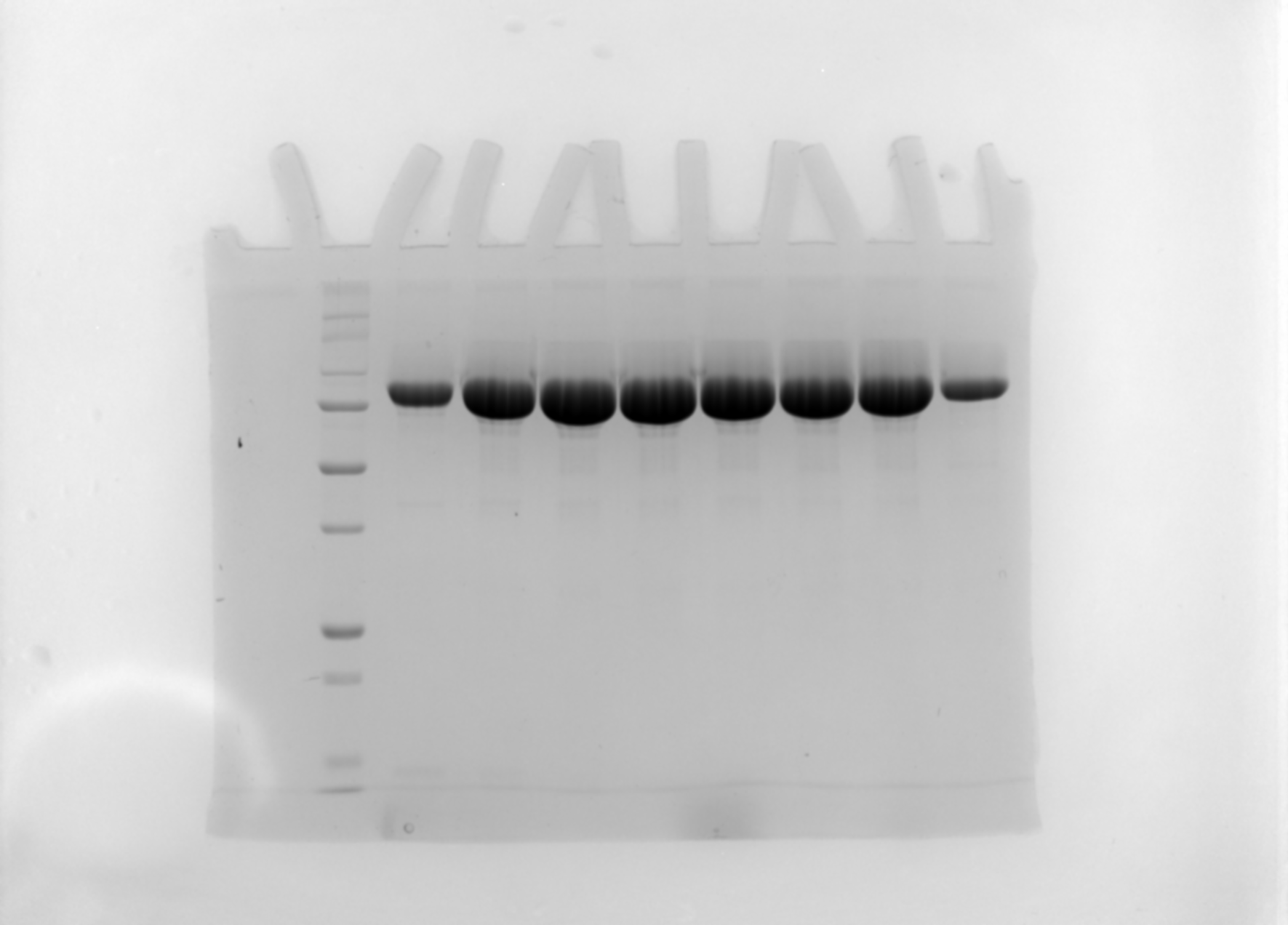

Supplement: Figure 1—figure supplement 1—source data 2. [file elife-104906-fig1-figsupp1-data2.zip › Figure 1-figure supplement 1-source data 2/Figure 1 - figure supplement 1 source data 2 - coomassie.tif]

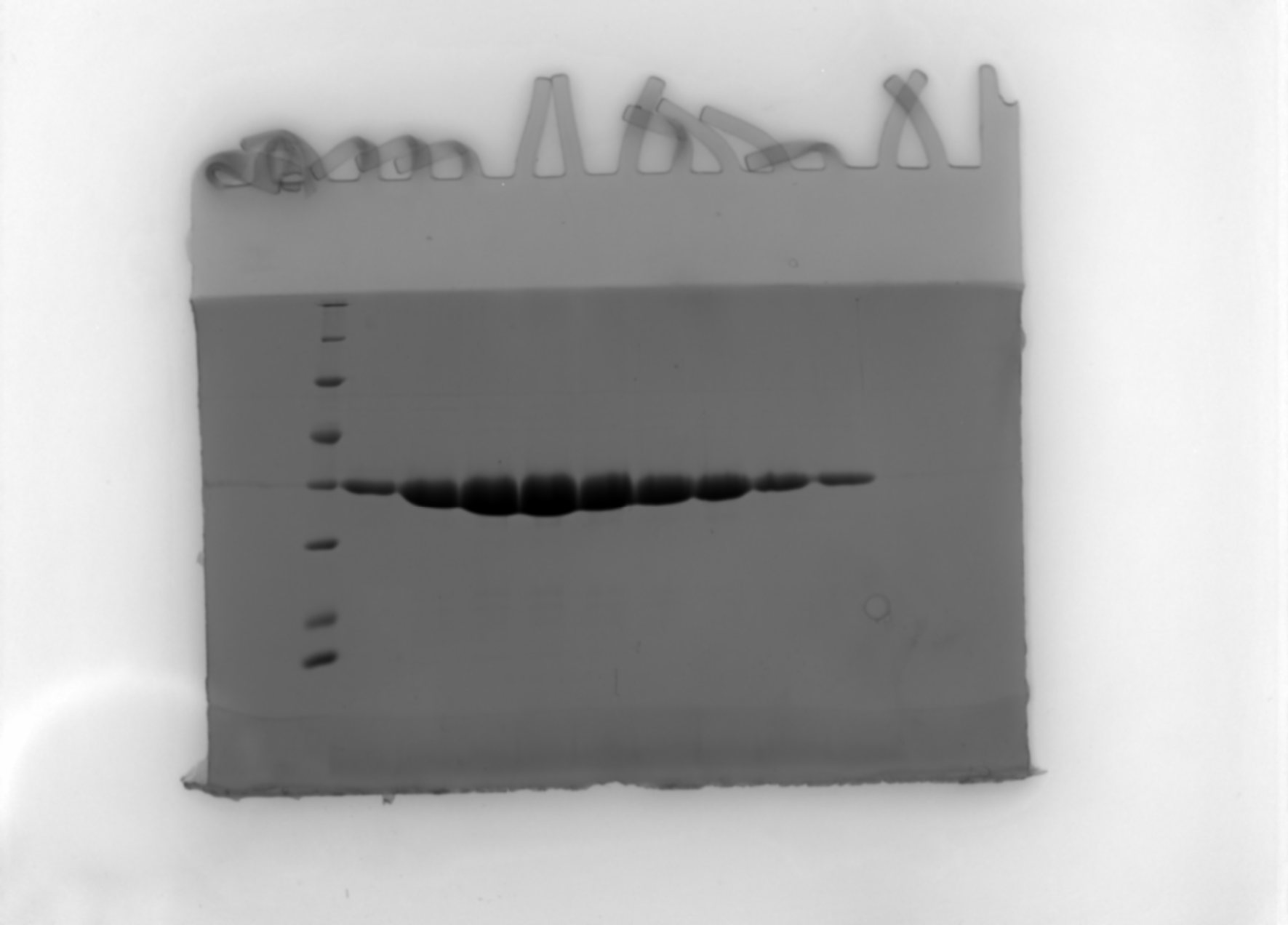

Supplement: Figure 2—figure supplement 1—source data 2. [file elife-104906-fig2-figsupp1-data2.zip › Figure 2-figure supplement 1-source data 2/Figure 2 - figure supplement 1 source data 2- coomassie.tif]

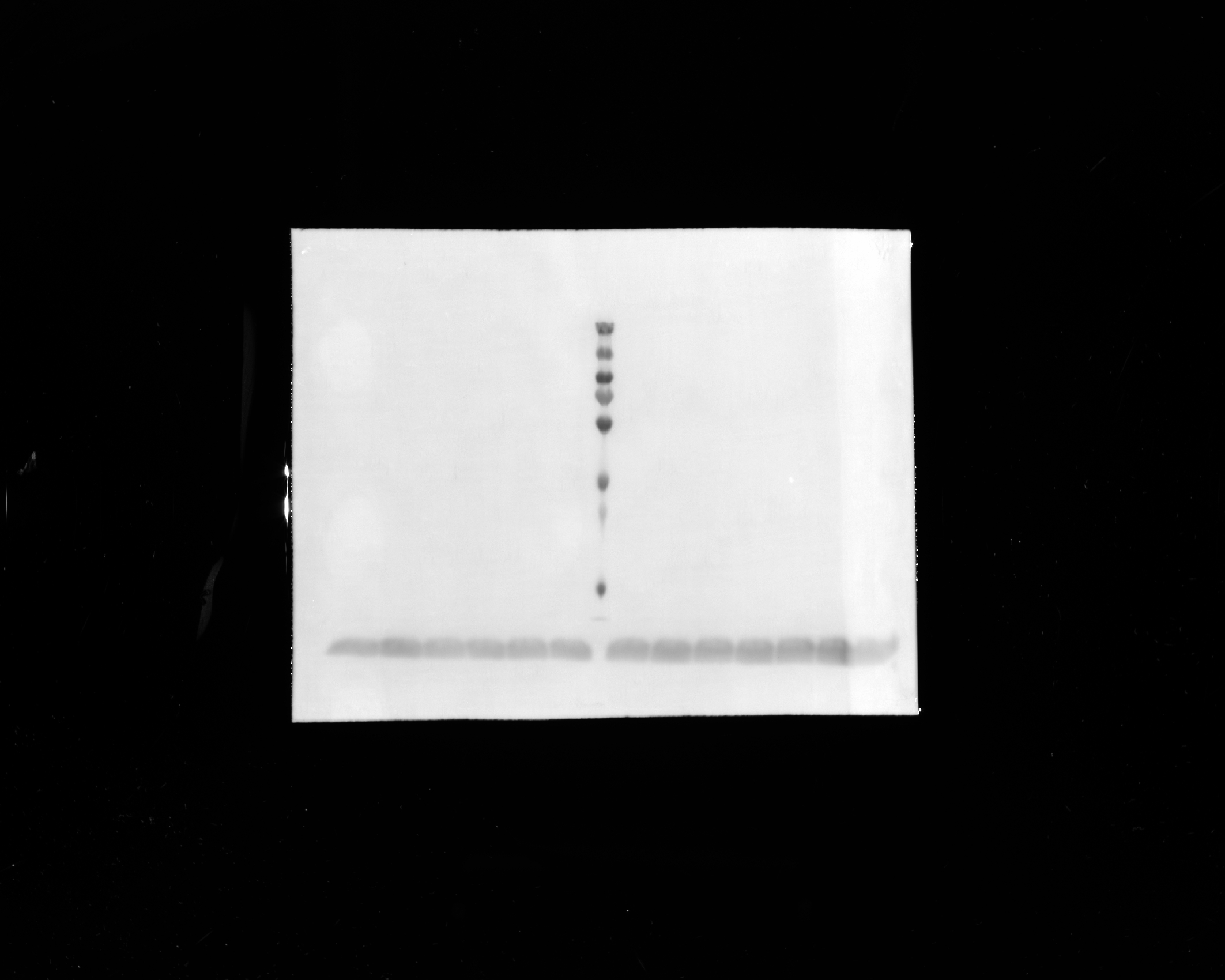

Supplement: Figure 3—source data 2. [file elife-104906-fig3-data2.zip › Figure 3-source data 2/Figure 3 source data 2 Panel A- blot.tif]

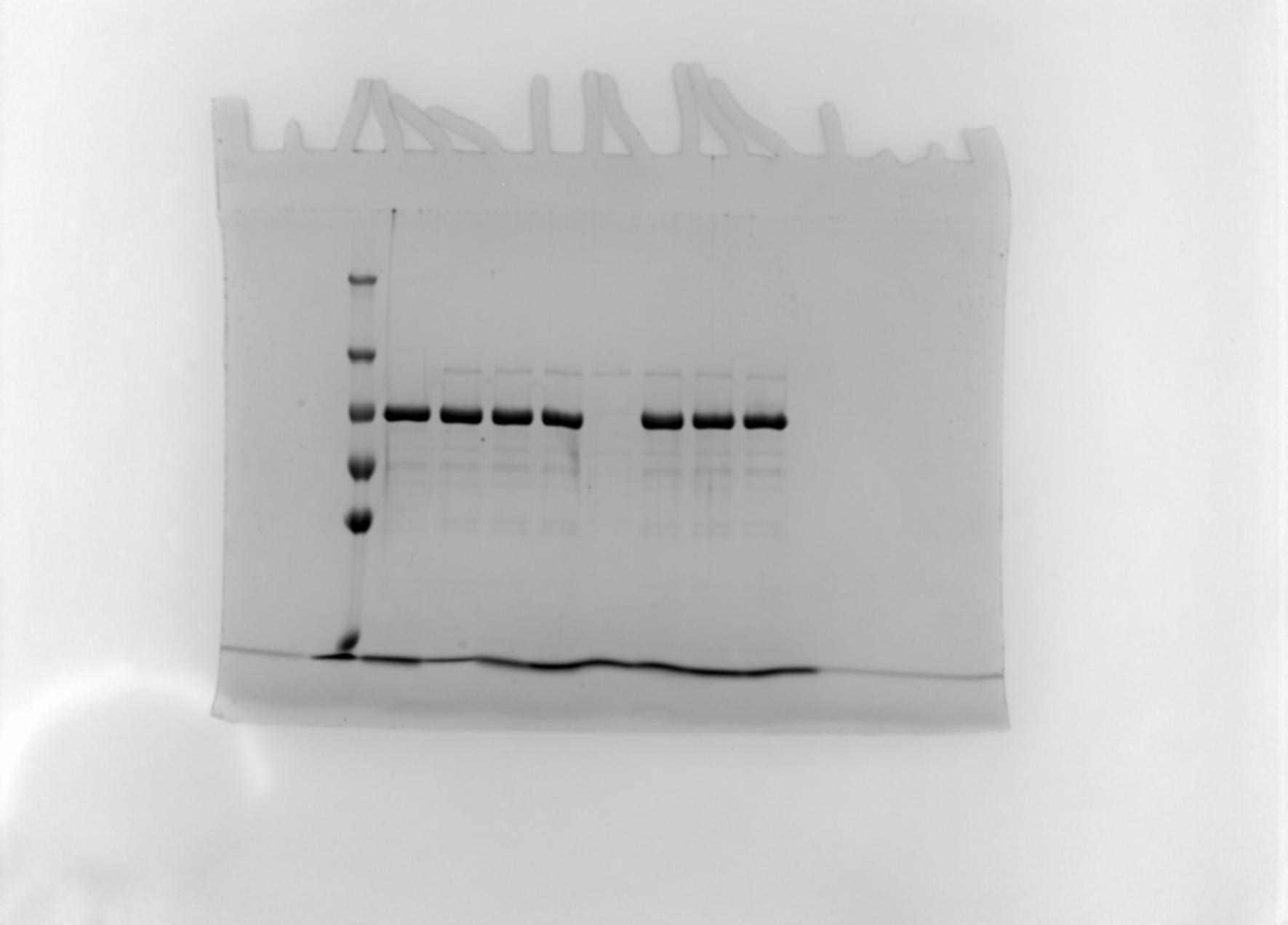

Supplement: Figure 3—source data 2. [file elife-104906-fig3-data2.zip › Figure 3-source data 2/Figure 3 source data 2 Panel D - coomassie.tif]

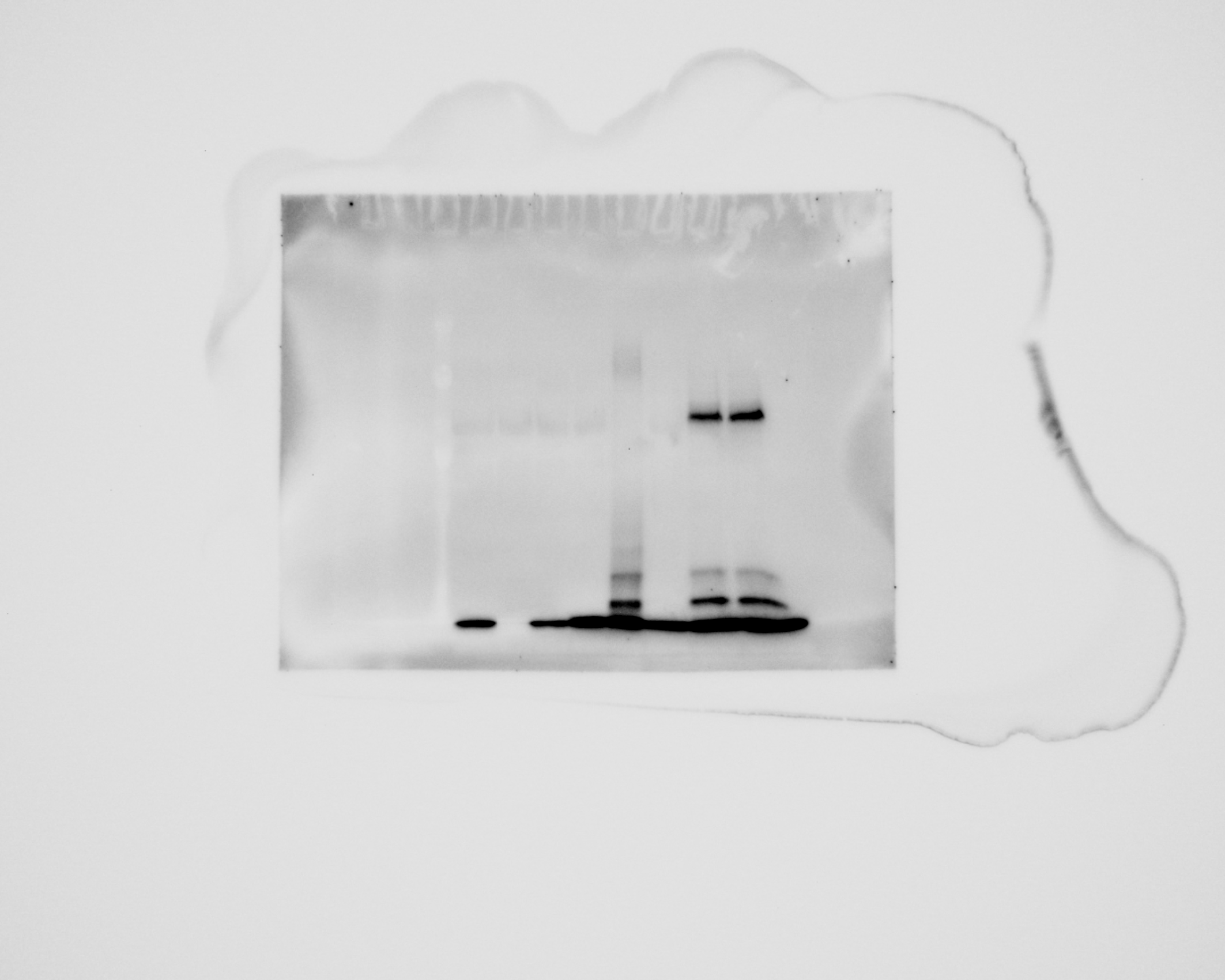

Supplement: Figure 3—source data 2. [file elife-104906-fig3-data2.zip › Figure 3-source data 2/Figure 3 source data 2 Panel D - ubiquitin.tif]

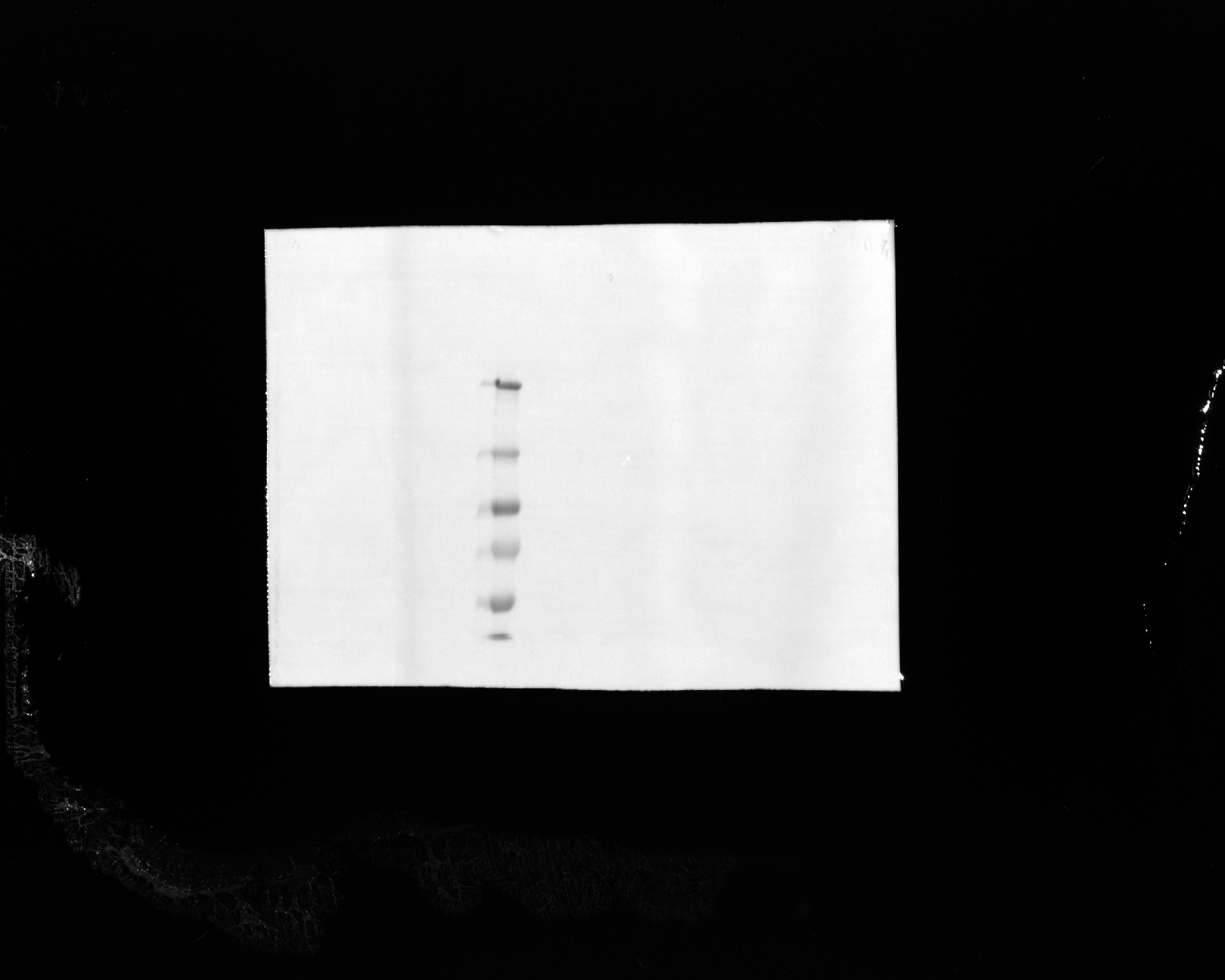

Supplement: Figure 3—source data 2. [file elife-104906-fig3-data2.zip › Figure 3-source data 2/Figure 3 source data 2 Panel F- blot.tif]

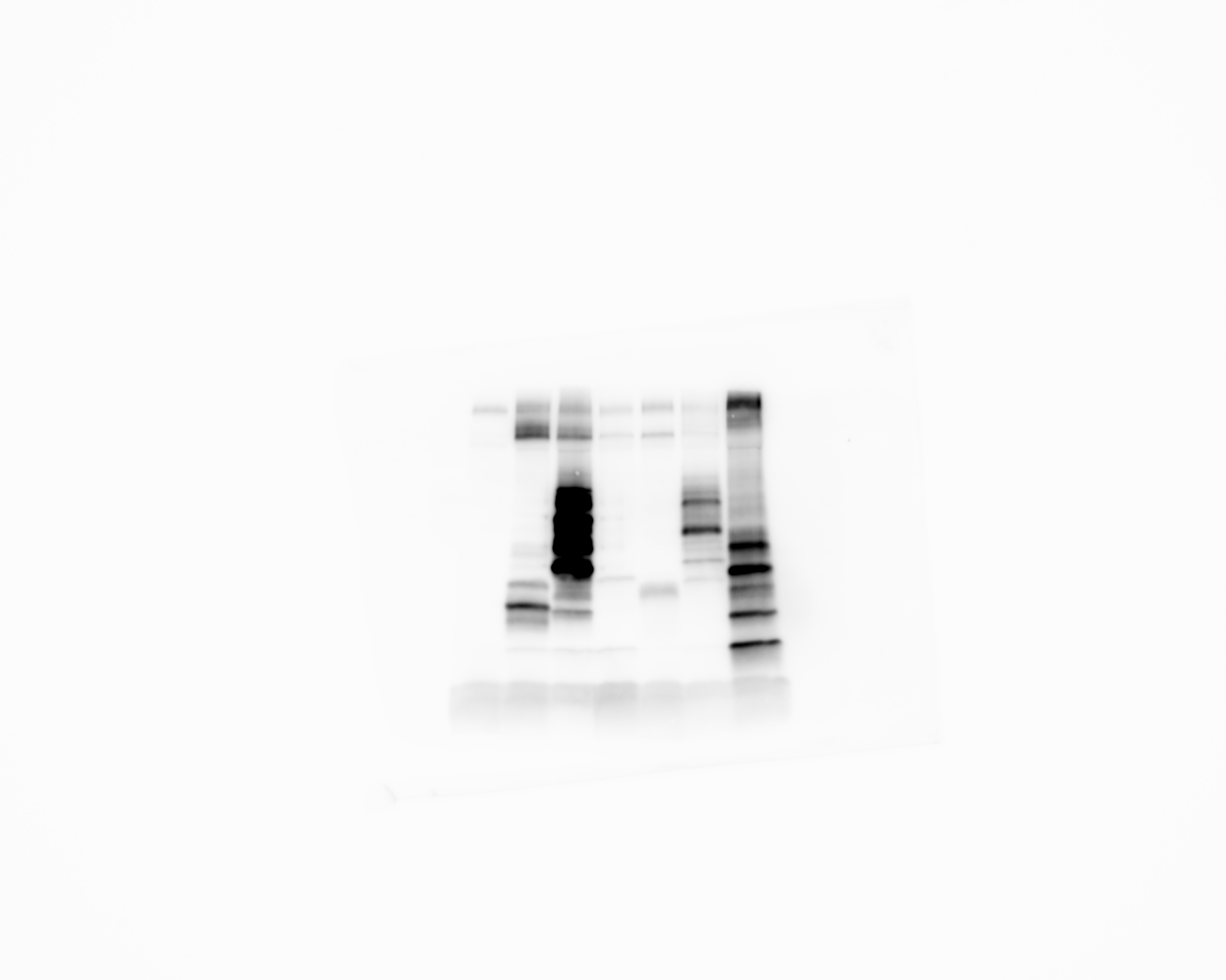

Supplement: Figure 3—source data 2. [file elife-104906-fig3-data2.zip › Figure 3-source data 2/Figure 3 source data 2 Panel B- ubiquitin 2..tif]

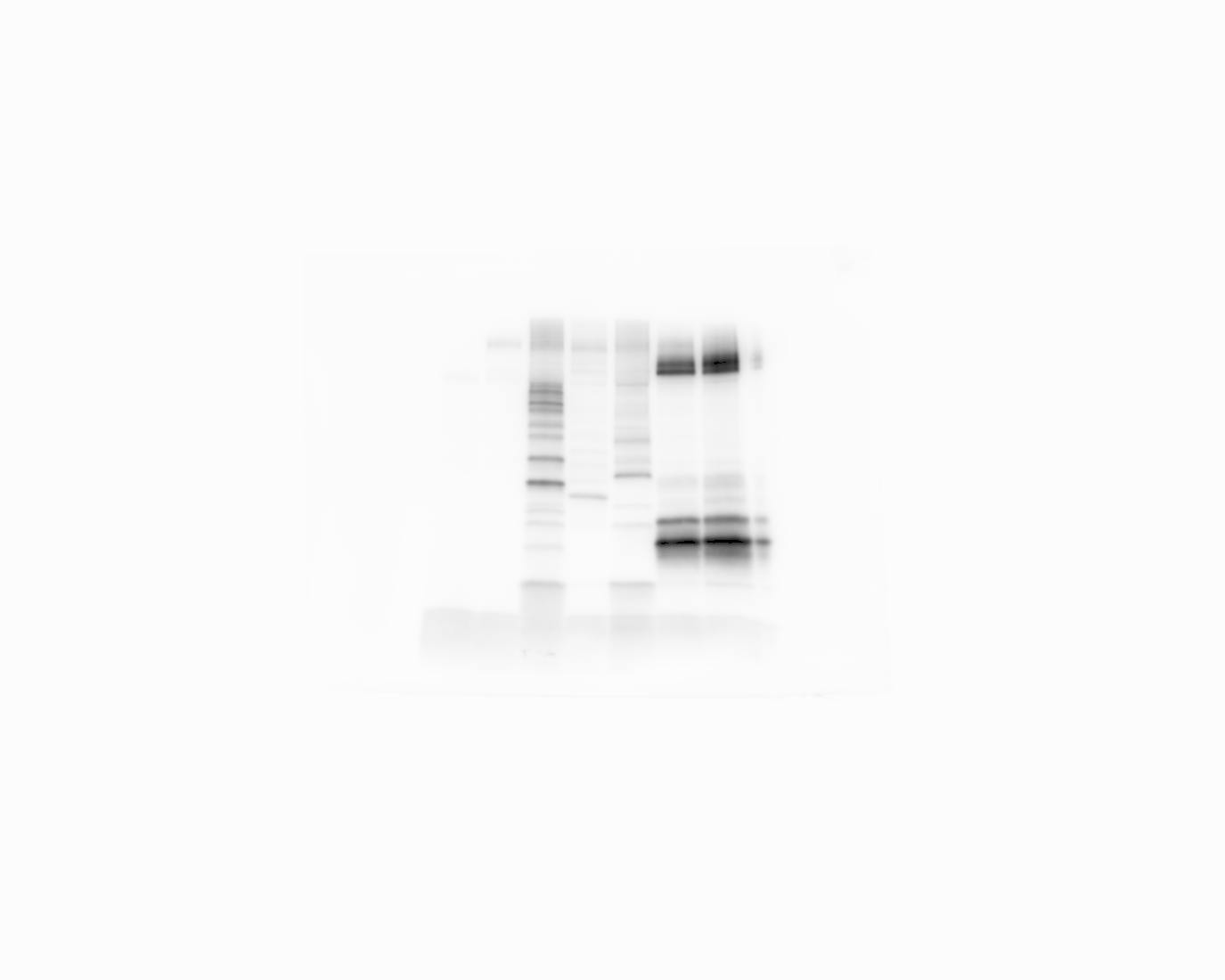

Supplement: Figure 3—source data 2. [file elife-104906-fig3-data2.zip › Figure 3-source data 2/Figure 3 source data 2 Panel B- ubiquitin 1.tif]

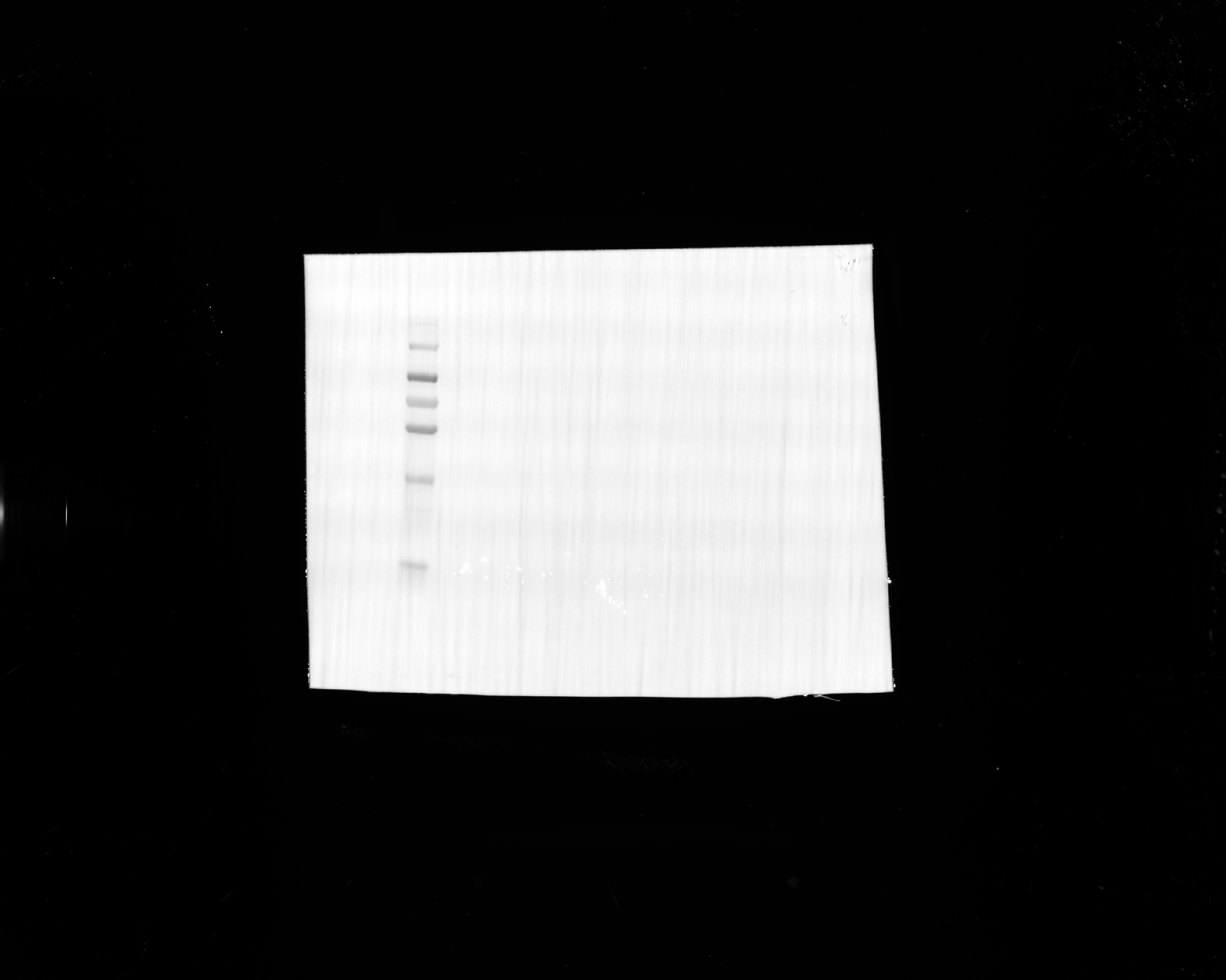

Supplement: Figure 3—source data 2. [file elife-104906-fig3-data2.zip › Figure 3-source data 2/Figure 3 source data 2 PanelB- blot 1.tif]

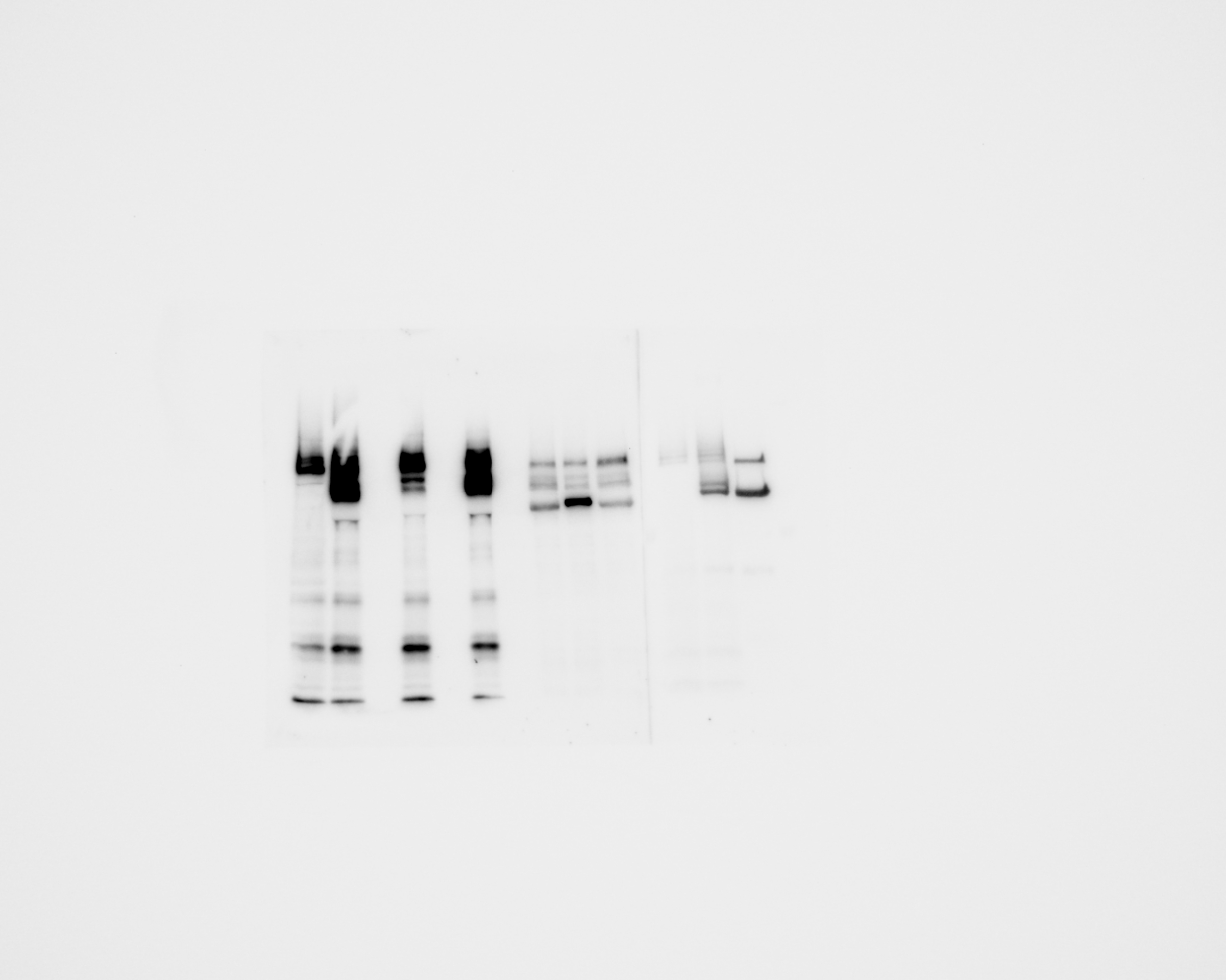

Supplement: Figure 3—source data 2. [file elife-104906-fig3-data2.zip › Figure 3-source data 2/Figure 3 source data 2 Panel C- His.tif]

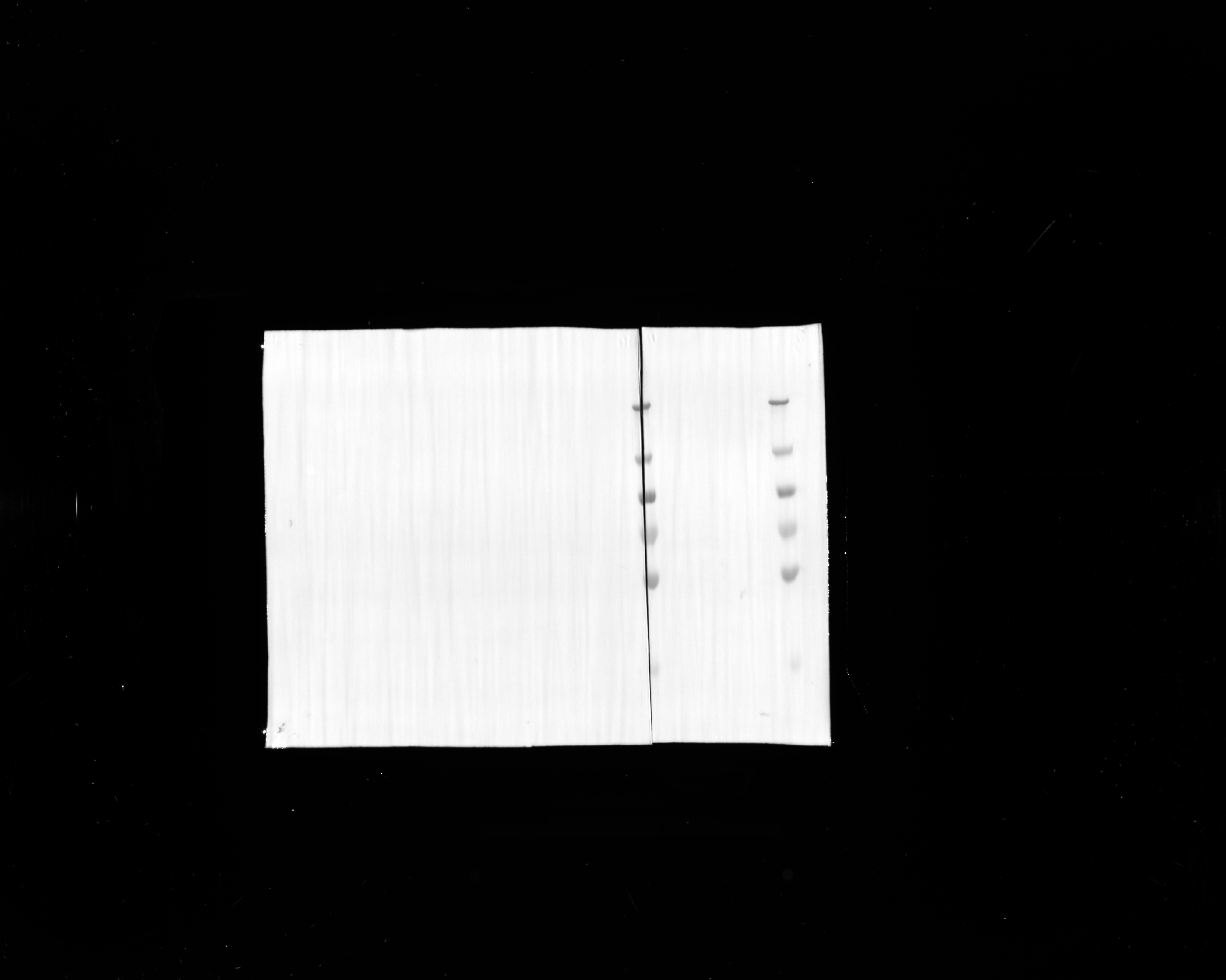

Supplement: Figure 3—source data 2. [file elife-104906-fig3-data2.zip › Figure 3-source data 2/Figure 3 source data 2 Panel C- blot.tif]

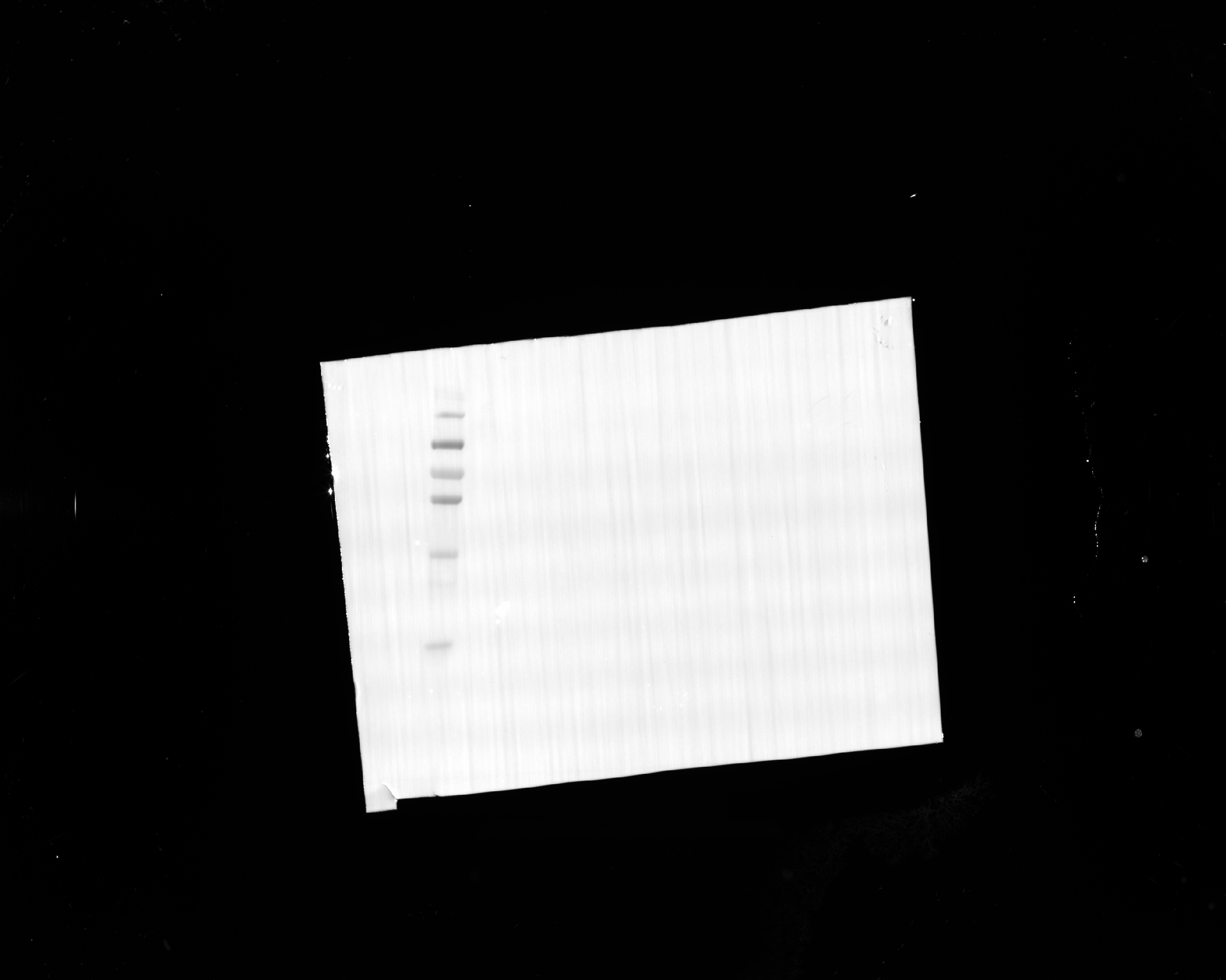

Supplement: Figure 3—source data 2. [file elife-104906-fig3-data2.zip › Figure 3-source data 2/Figure 3 source data 2 Panel B- blot 2.tif]

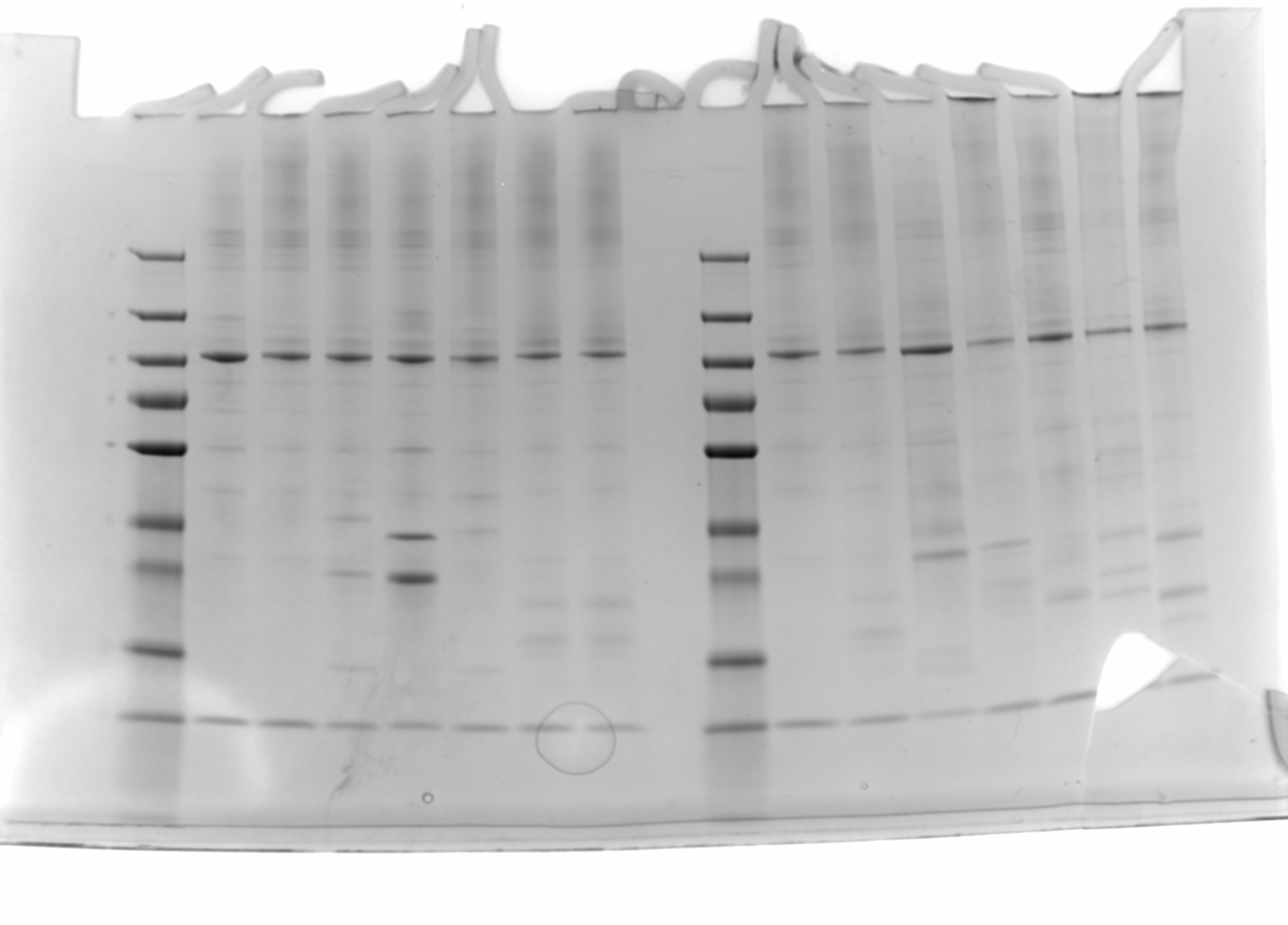

Supplement: Figure 3—source data 2. [file elife-104906-fig3-data2.zip › Figure 3-source data 2/Figure 3 source data 2 Panel B- coomassie.tif]

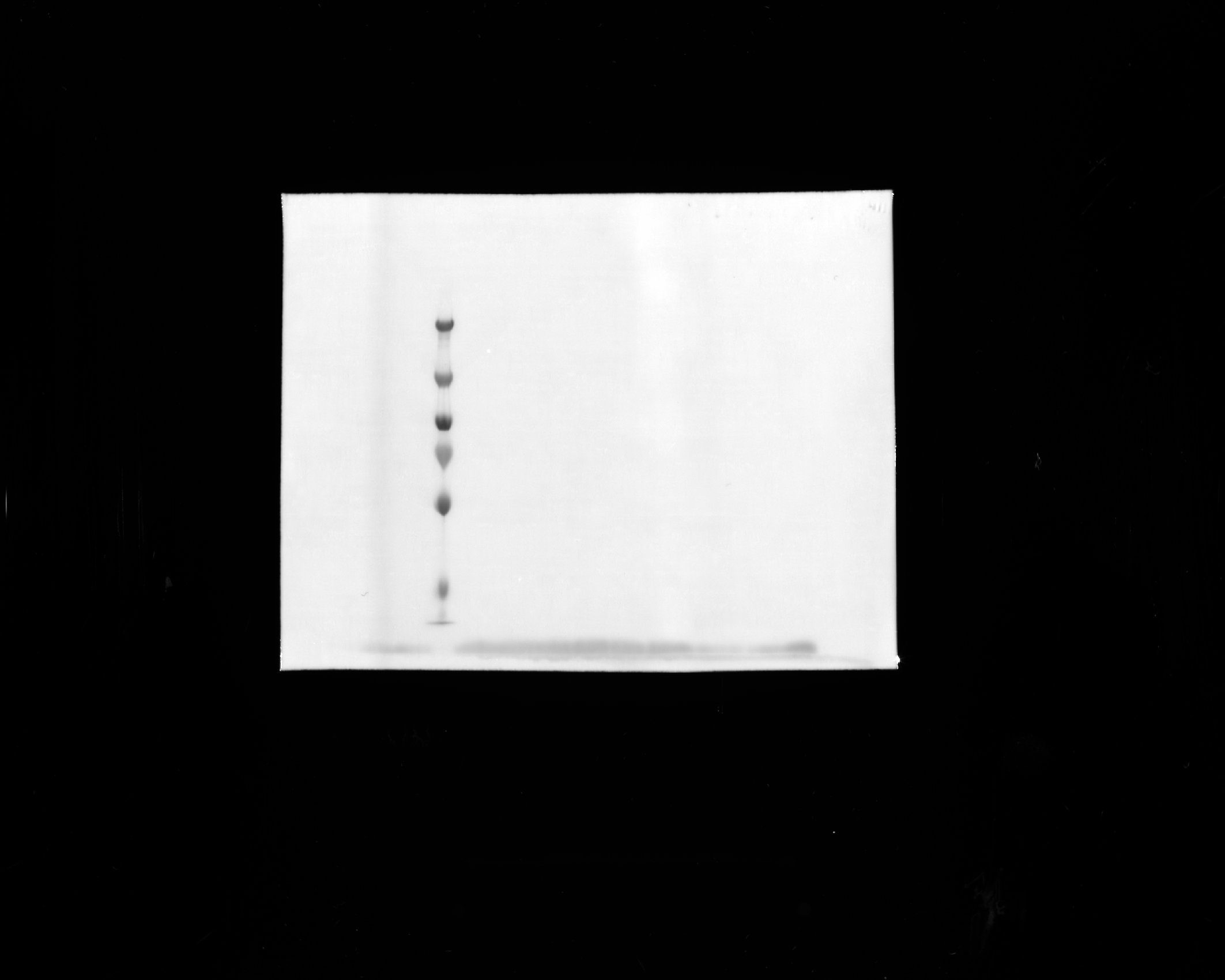

Supplement: Figure 3—source data 2. [file elife-104906-fig3-data2.zip › Figure 3-source data 2/Figure 3 source data 2 Panel D- blot.tif]

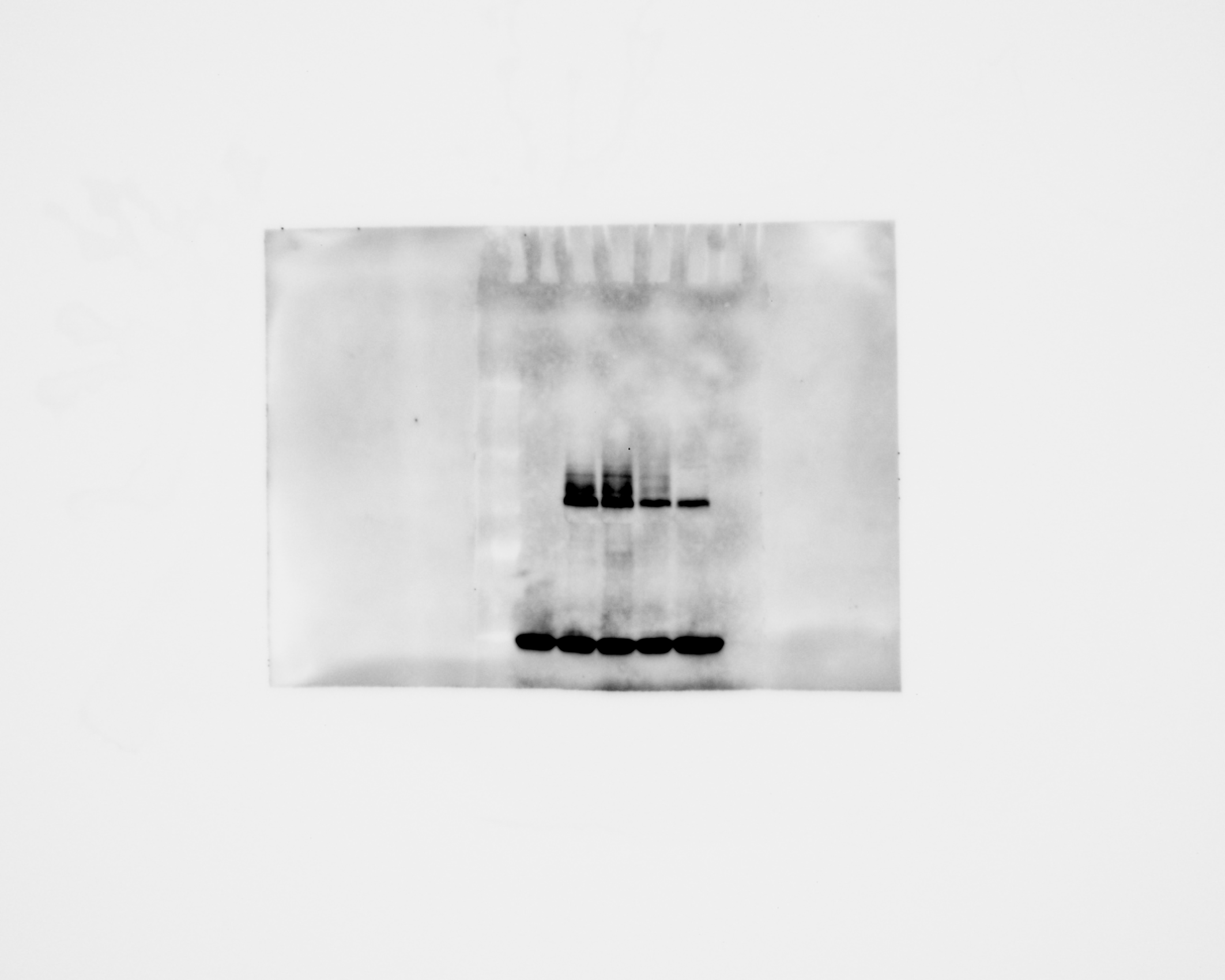

Supplement: Figure 3—source data 2. [file elife-104906-fig3-data2.zip › Figure 3-source data 2/Figure 3 source data 2 Panel F - ubiquitin.tif]

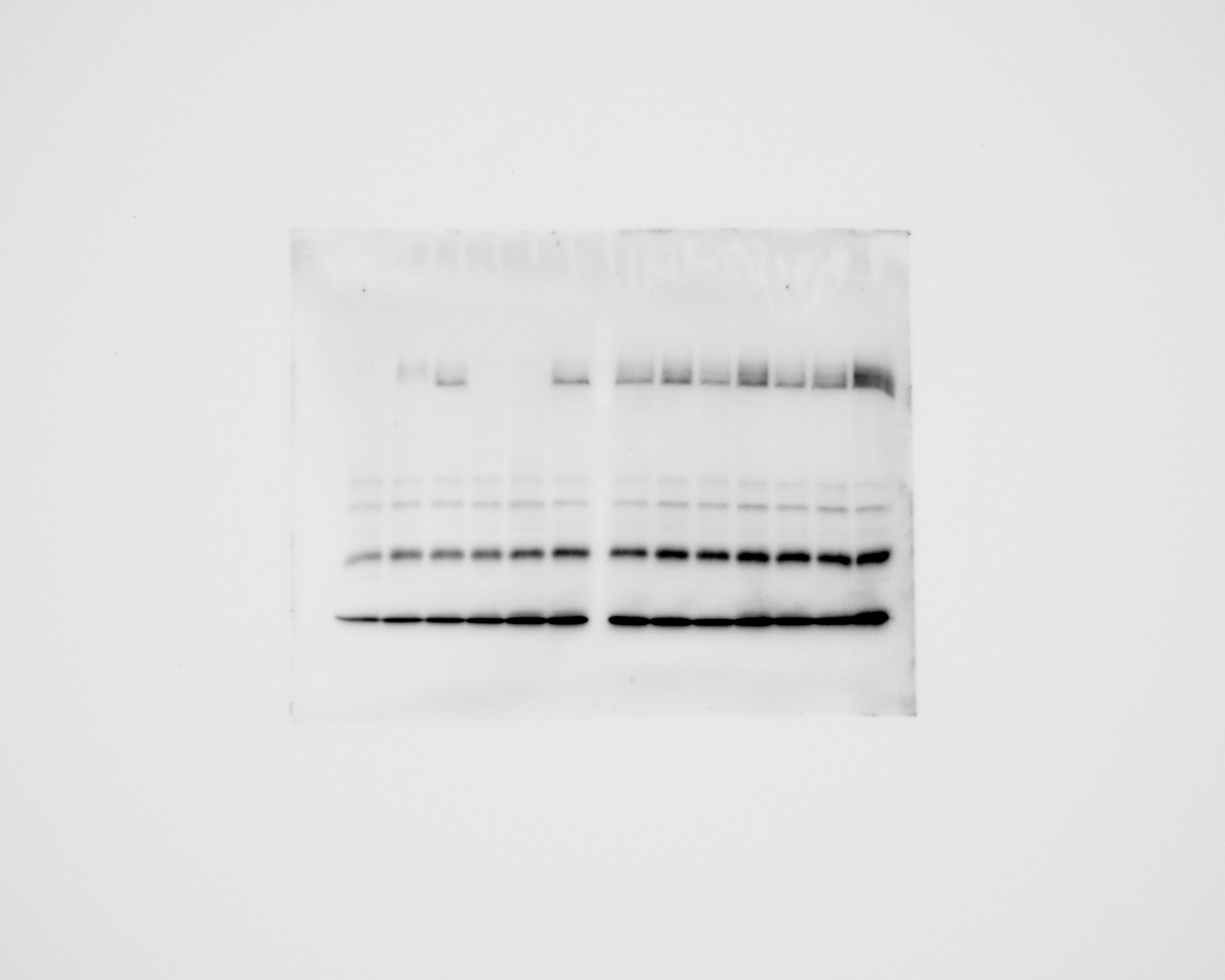

Supplement: Figure 3—source data 2. [file elife-104906-fig3-data2.zip › Figure 3-source data 2/Figure 3 source data 2 Panel A- ubiquitin.tif]

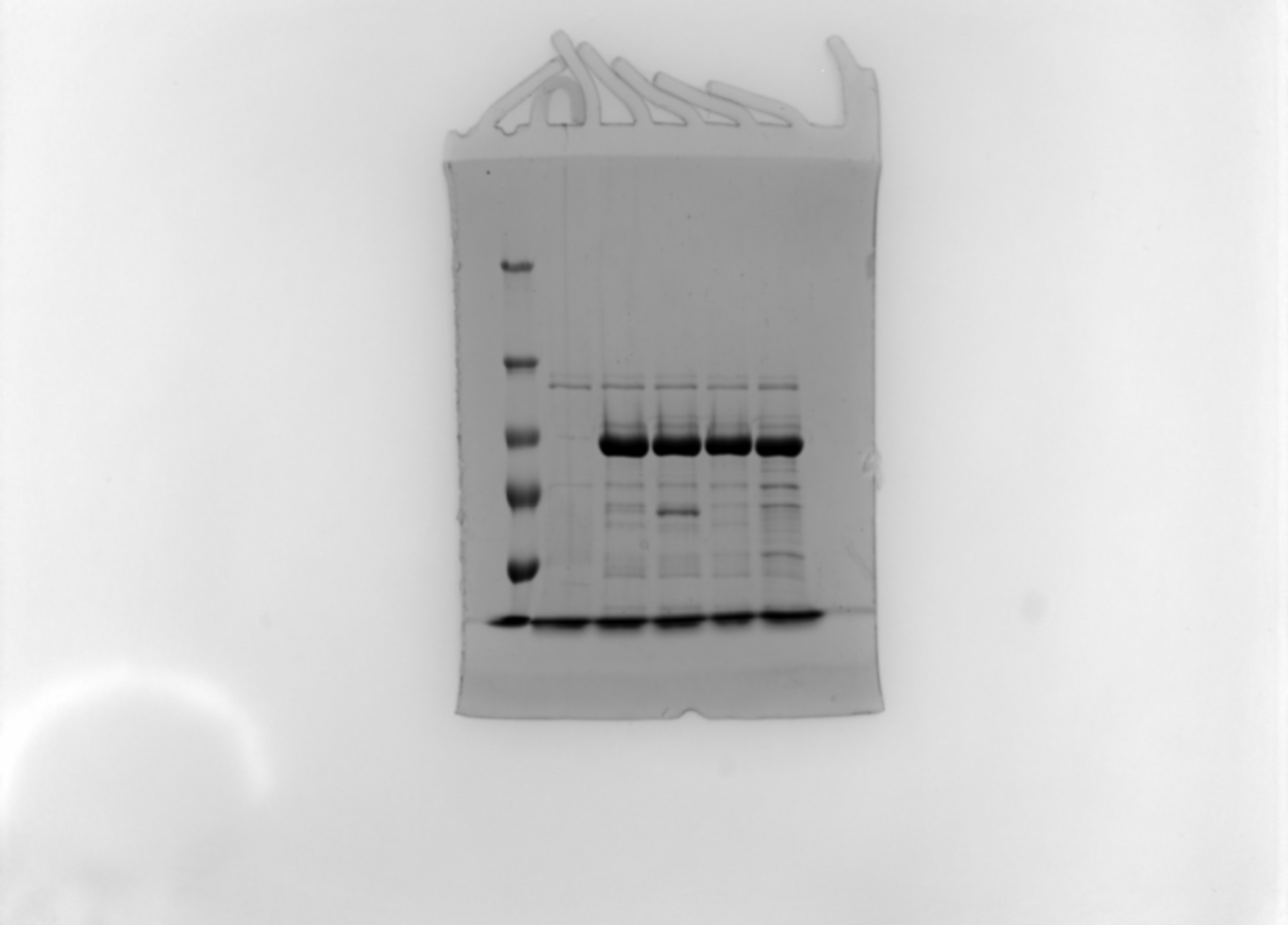

Supplement: Figure 3—source data 2. [file elife-104906-fig3-data2.zip › Figure 3-source data 2/Figure 3 source data 2 Panel F - coomassie.tif]

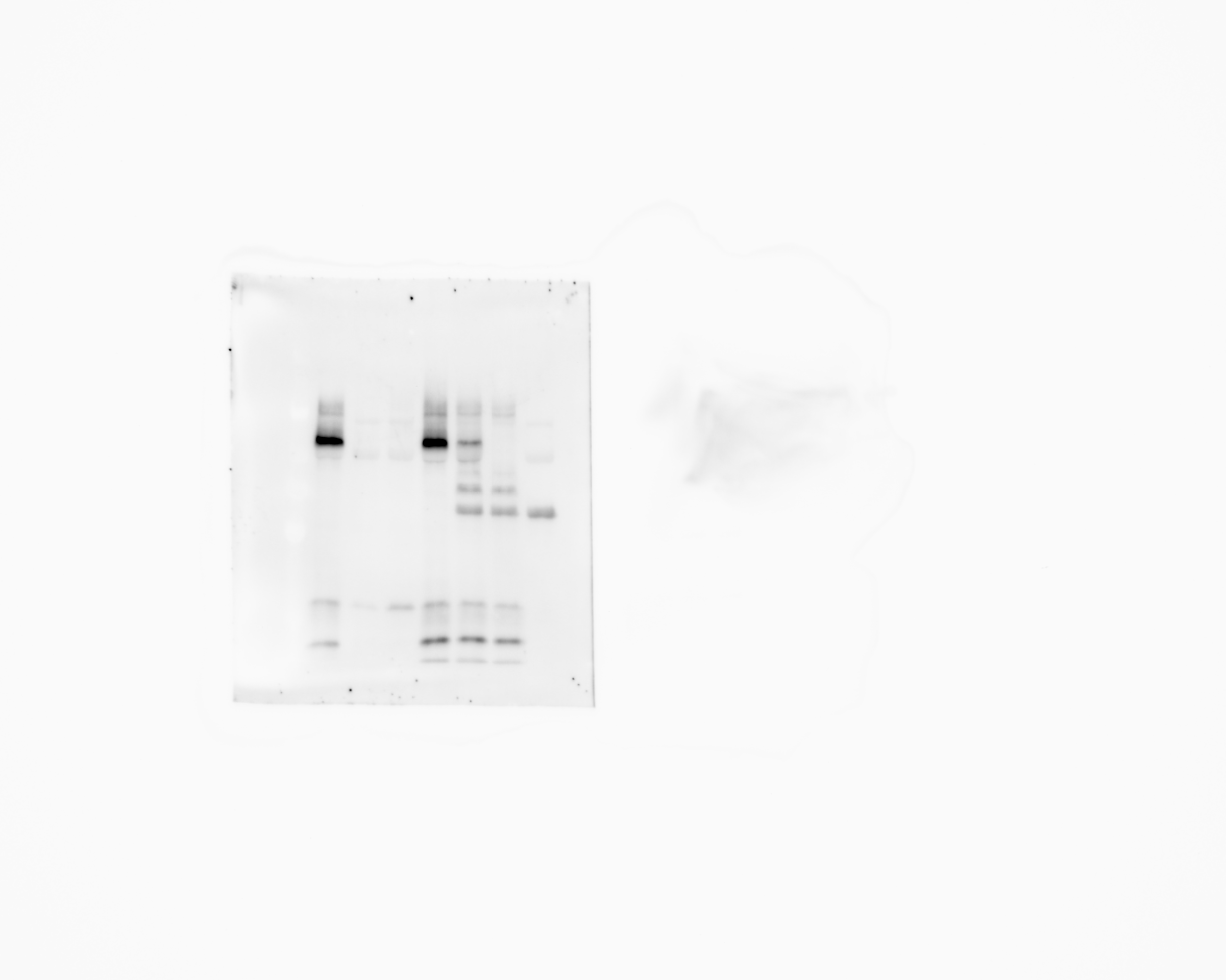

Supplement: Figure 3—figure supplement 1—source data 2. [file elife-104906-fig3-figsupp1-data2.zip › Figure 3-figure supplement 1-source data 2/Figure 3 - figure supplement 1 Source data 2 Panel A- ubiquitin.tif]

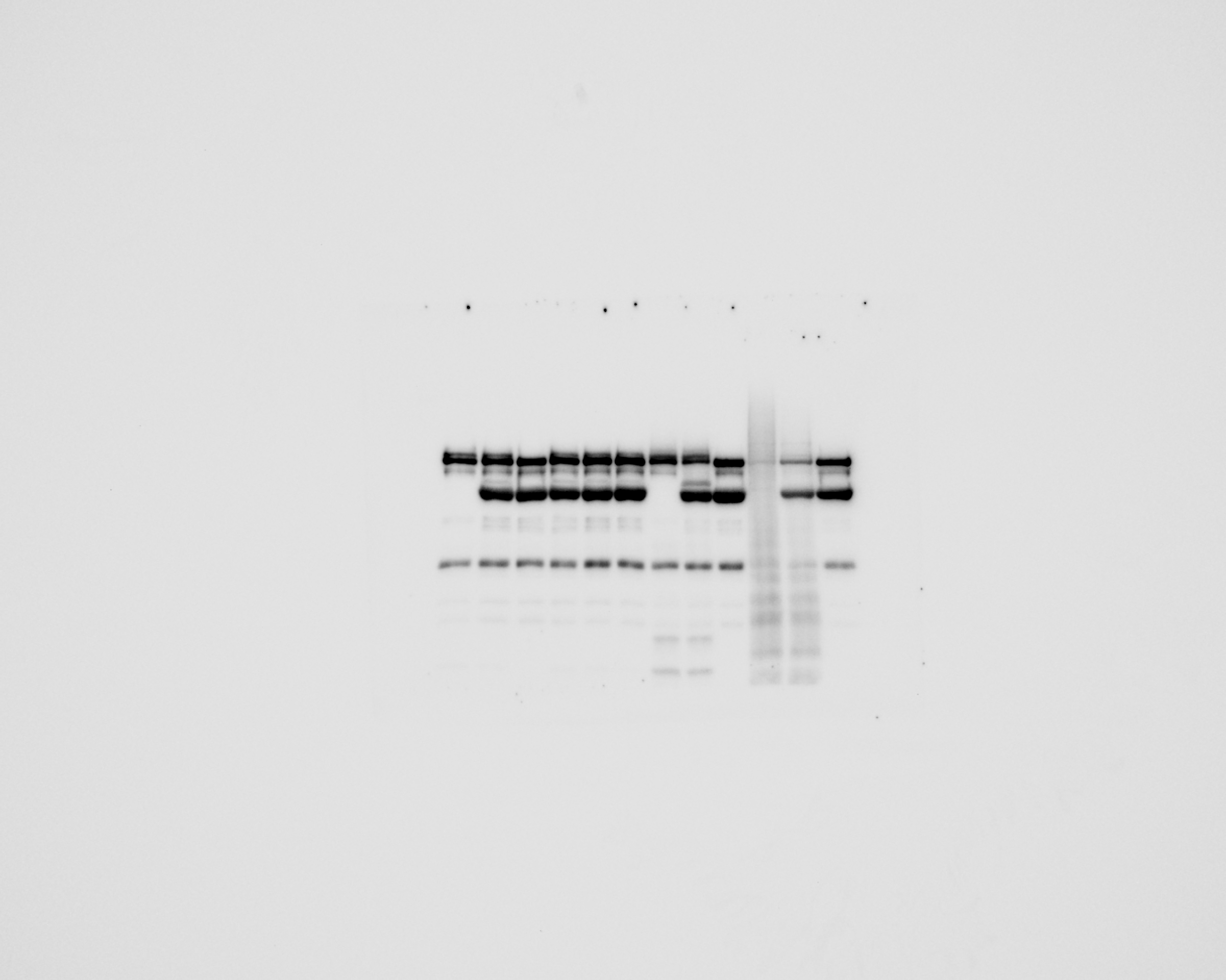

Supplement: Figure 3—figure supplement 1—source data 2. [file elife-104906-fig3-figsupp1-data2.zip › Figure 3-figure supplement 1-source data 2/Figure 3 - figure supplement 1 Source data 2 Panel B- His.tif]

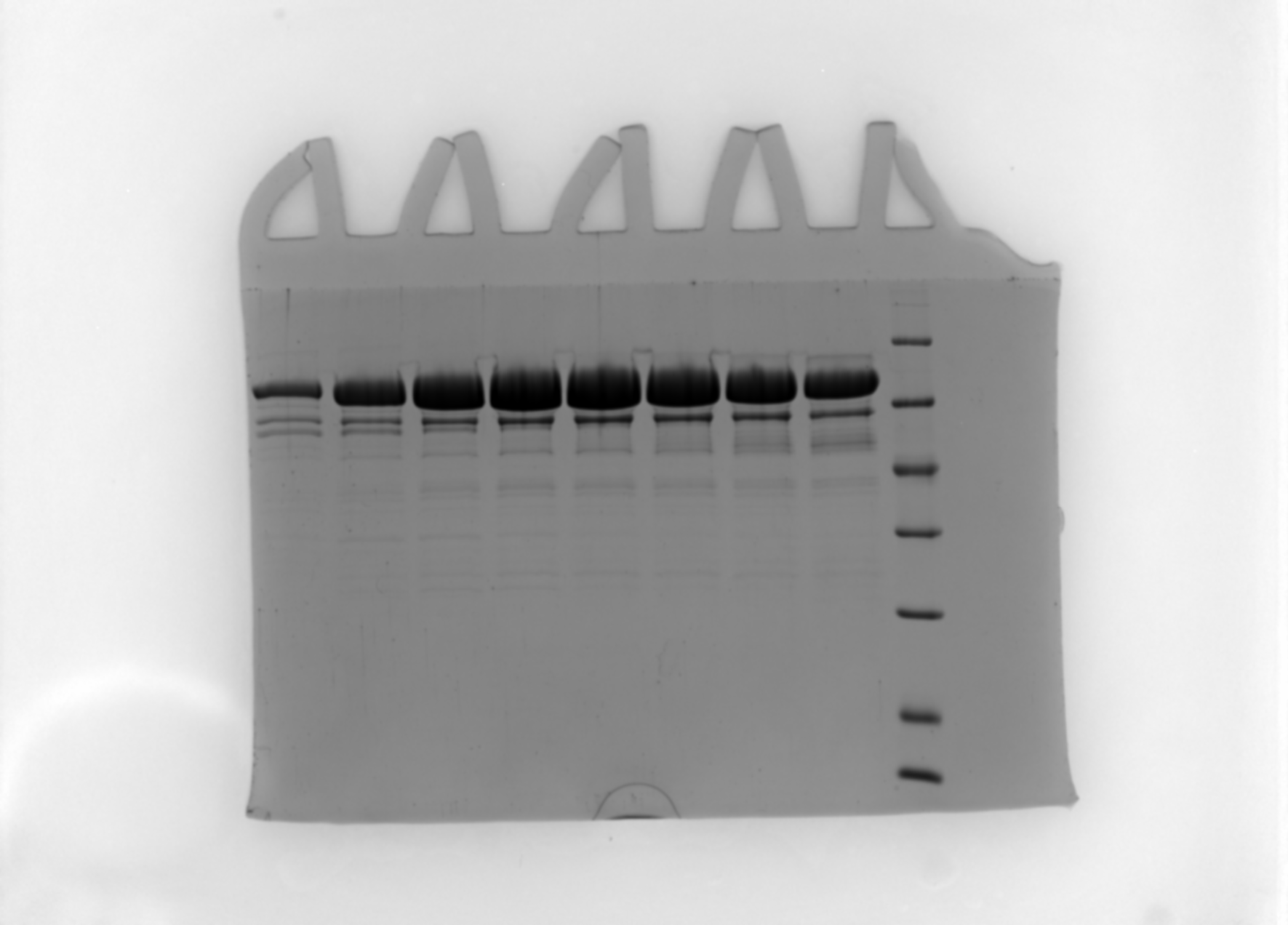

Supplement: Figure 3—figure supplement 1—source data 2. [file elife-104906-fig3-figsupp1-data2.zip › Figure 3-figure supplement 1-source data 2/Figure 3 - figure supplement 1 source data 2 Panel D- coomassie 2.tif]

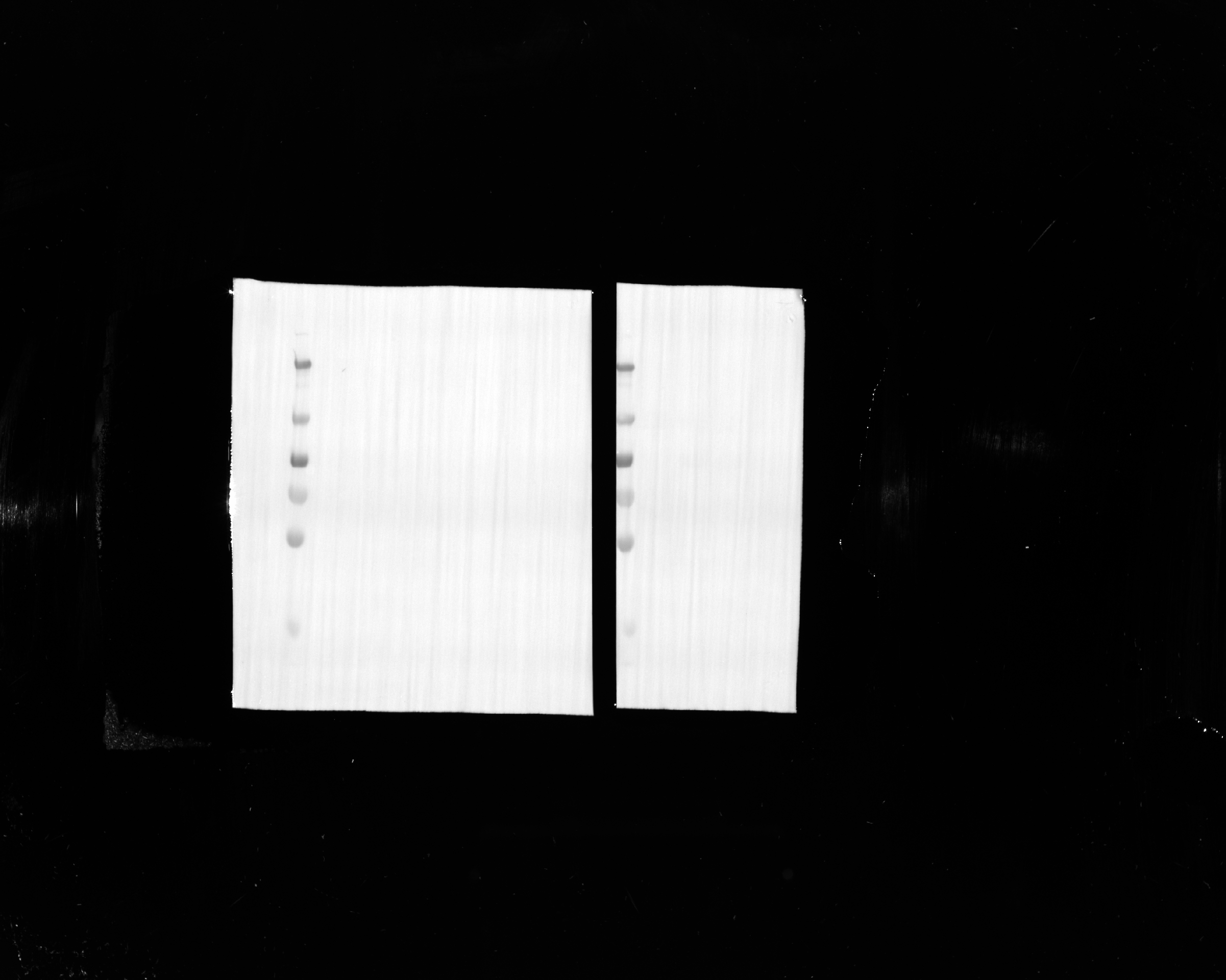

Supplement: Figure 3—figure supplement 1—source data 2. [file elife-104906-fig3-figsupp1-data2.zip › Figure 3-figure supplement 1-source data 2/Figure 3 - figure supplement 1 Source data 2 Panel A - blot.tif]

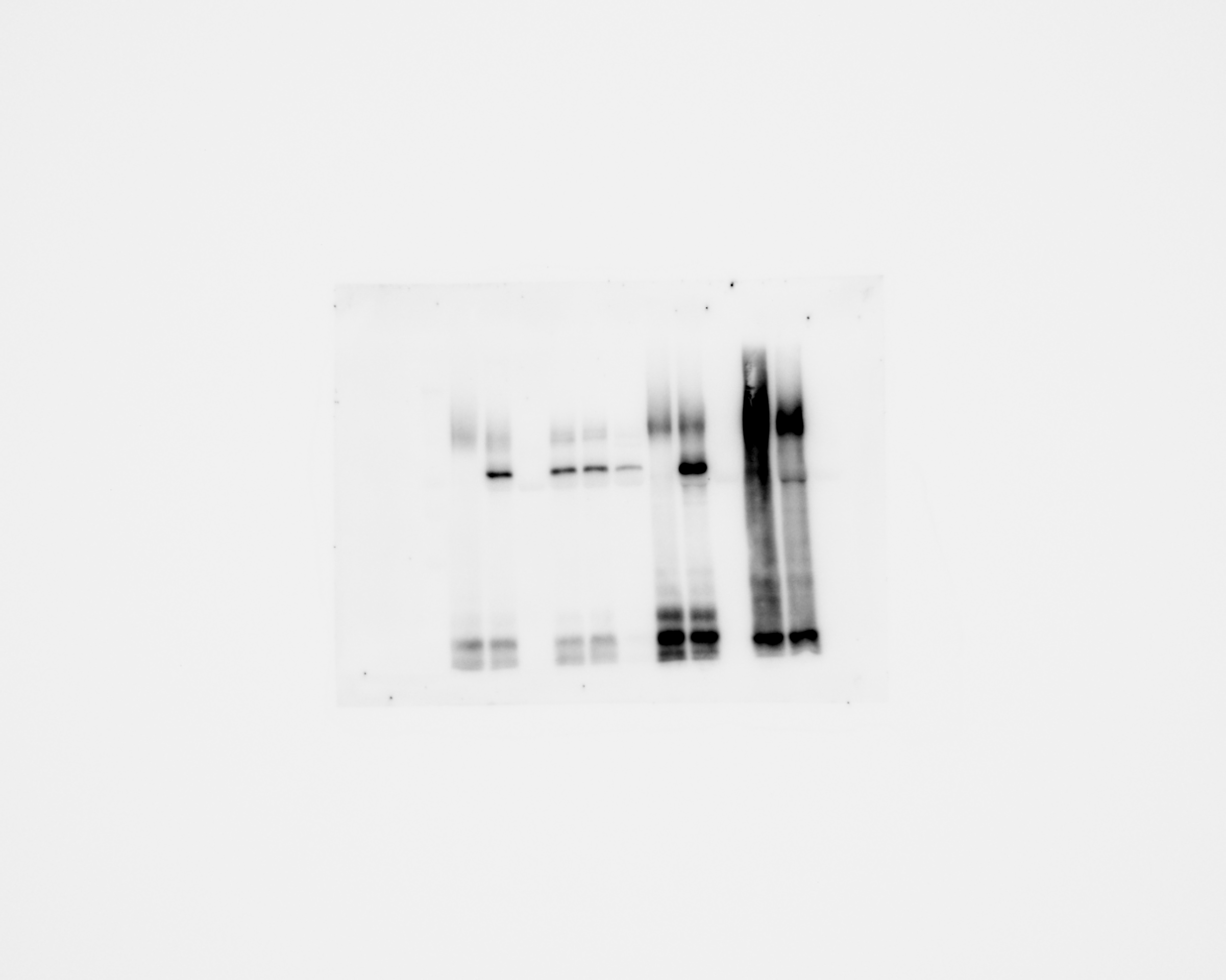

Supplement: Figure 3—figure supplement 1—source data 2. [file elife-104906-fig3-figsupp1-data2.zip › Figure 3-figure supplement 1-source data 2/Figure 3 - figure supplement 1 Source data 2 Panel B- ubiquitin 1.tif]

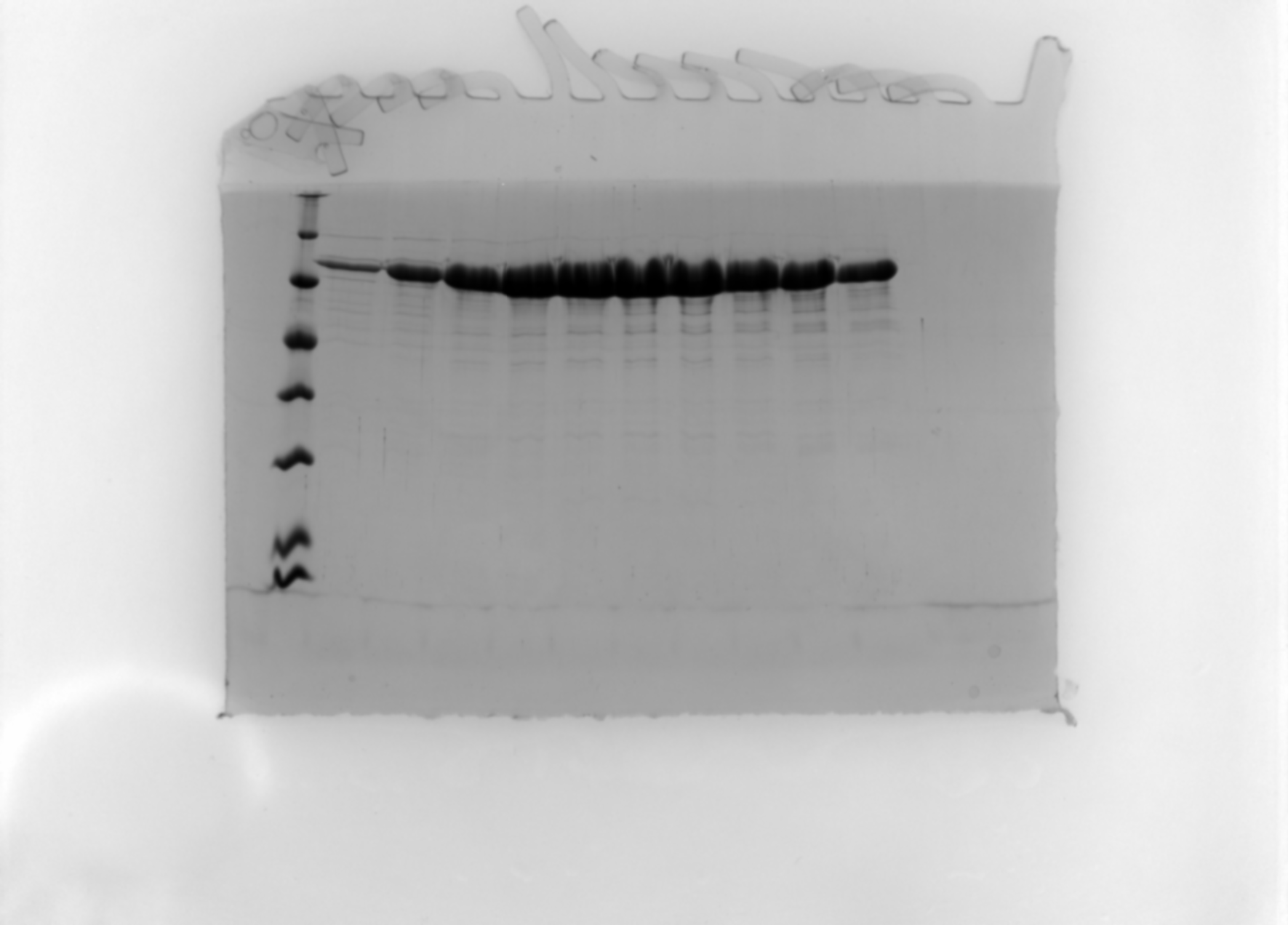

Supplement: Figure 3—figure supplement 1—source data 2. [file elife-104906-fig3-figsupp1-data2.zip › Figure 3-figure supplement 1-source data 2/Figure 3 - figure supplement 1 source data 2 Panel E- coomassie 3.tif]

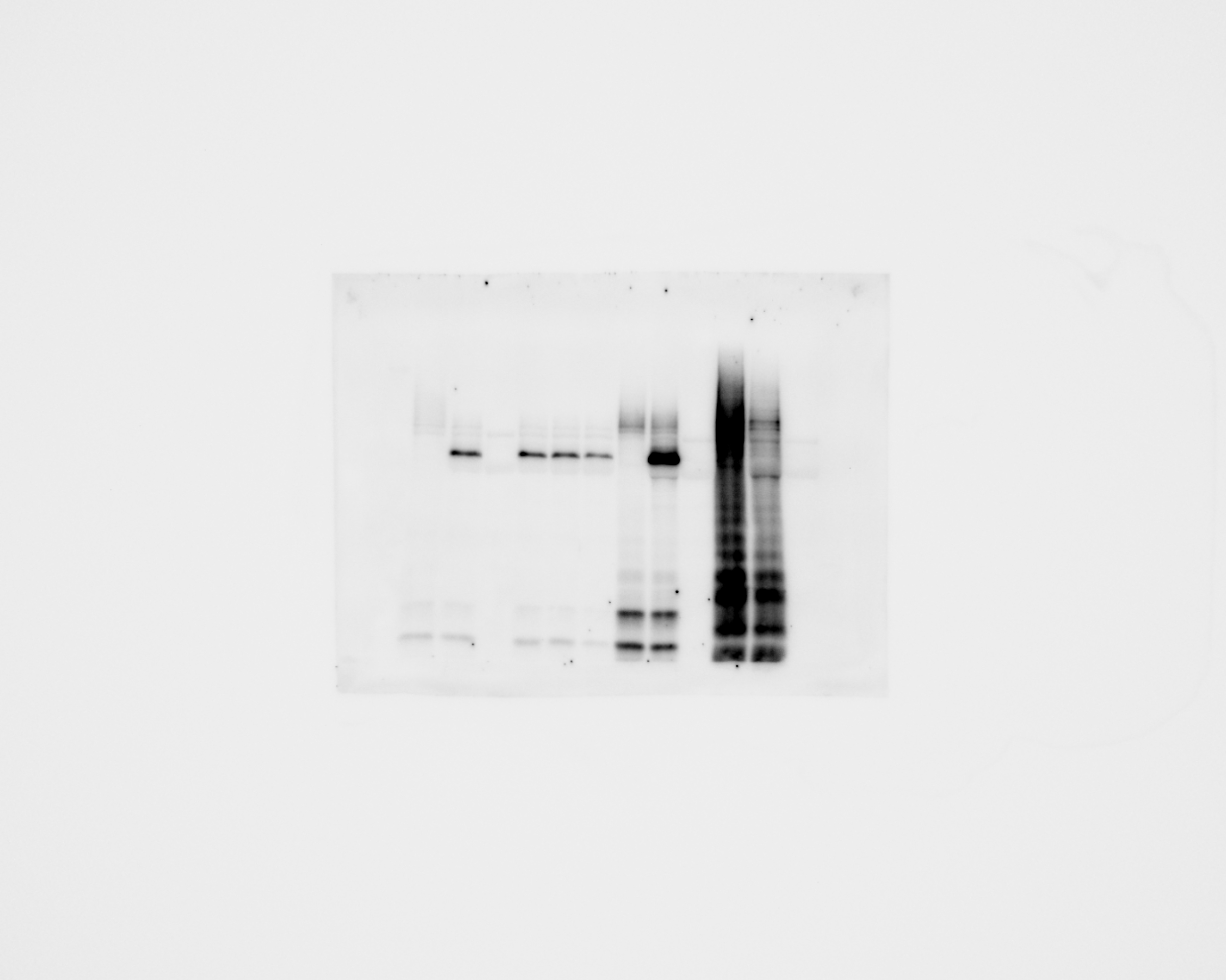

Supplement: Figure 3—figure supplement 1—source data 2. [file elife-104906-fig3-figsupp1-data2.zip › Figure 3-figure supplement 1-source data 2/Figure 3 - figure supplement 1 Source data 2 Panel B- ubiquitin 2.tif]

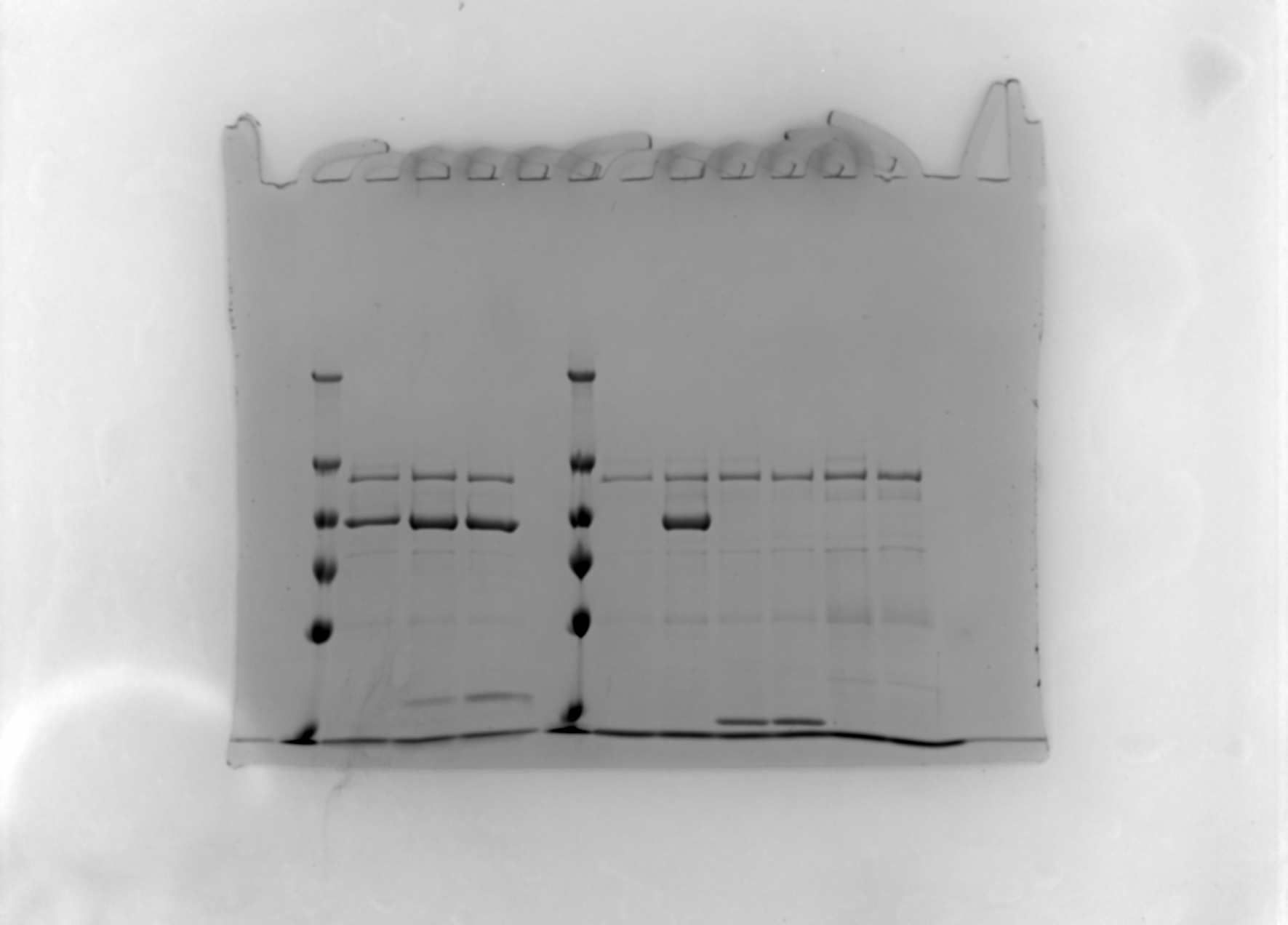

Supplement: Figure 3—figure supplement 1—source data 2. [file elife-104906-fig3-figsupp1-data2.zip › Figure 3-figure supplement 1-source data 2/Figure 3 - figure supplement 1 Source data 2 Panel A - coomassie.tif]

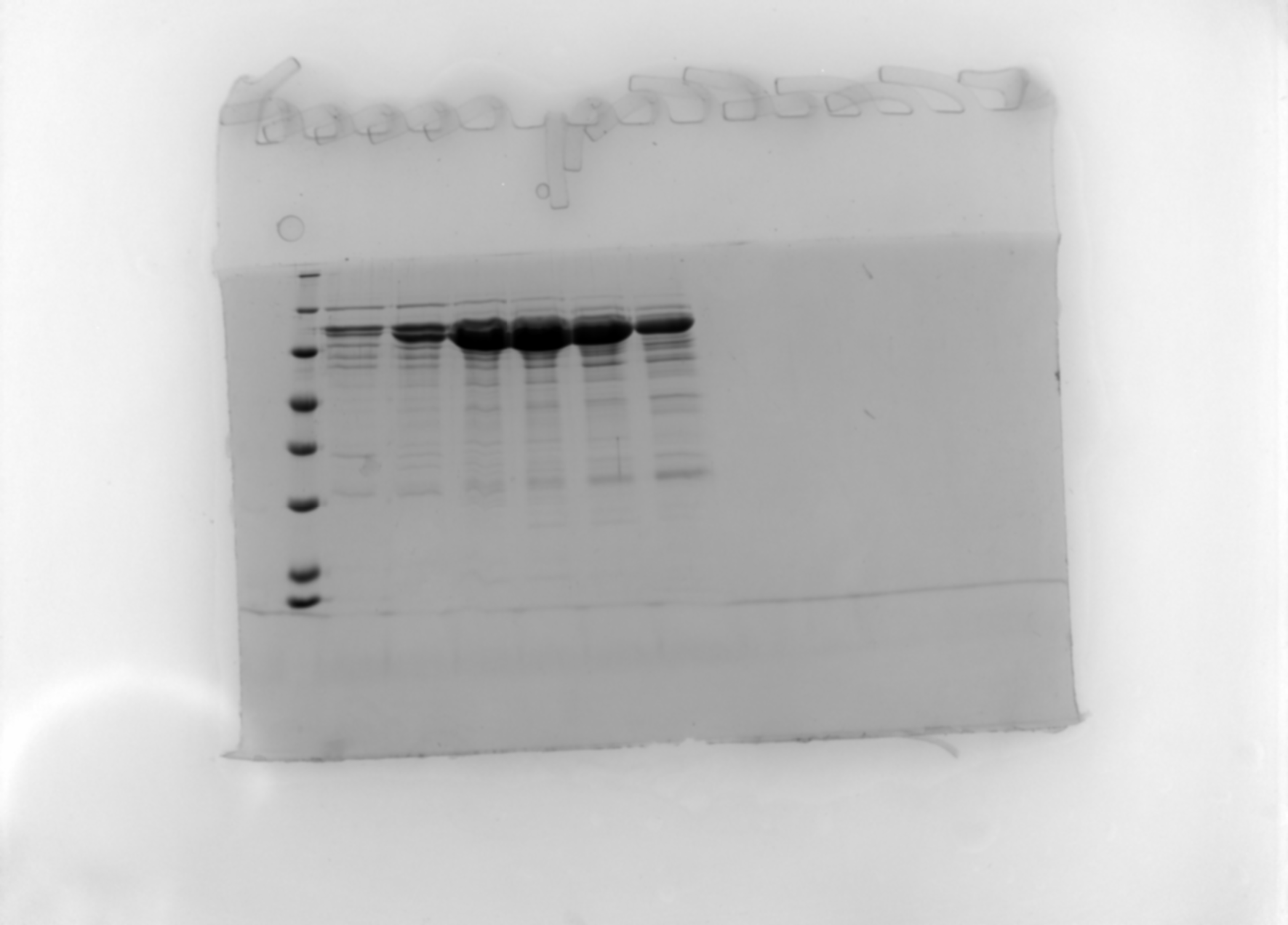

Supplement: Figure 3—figure supplement 1—source data 2. [file elife-104906-fig3-figsupp1-data2.zip › Figure 3-figure supplement 1-source data 2/Figure 3 - figure supplement 1 source data 2 Panel F- coomassie 4.tif]

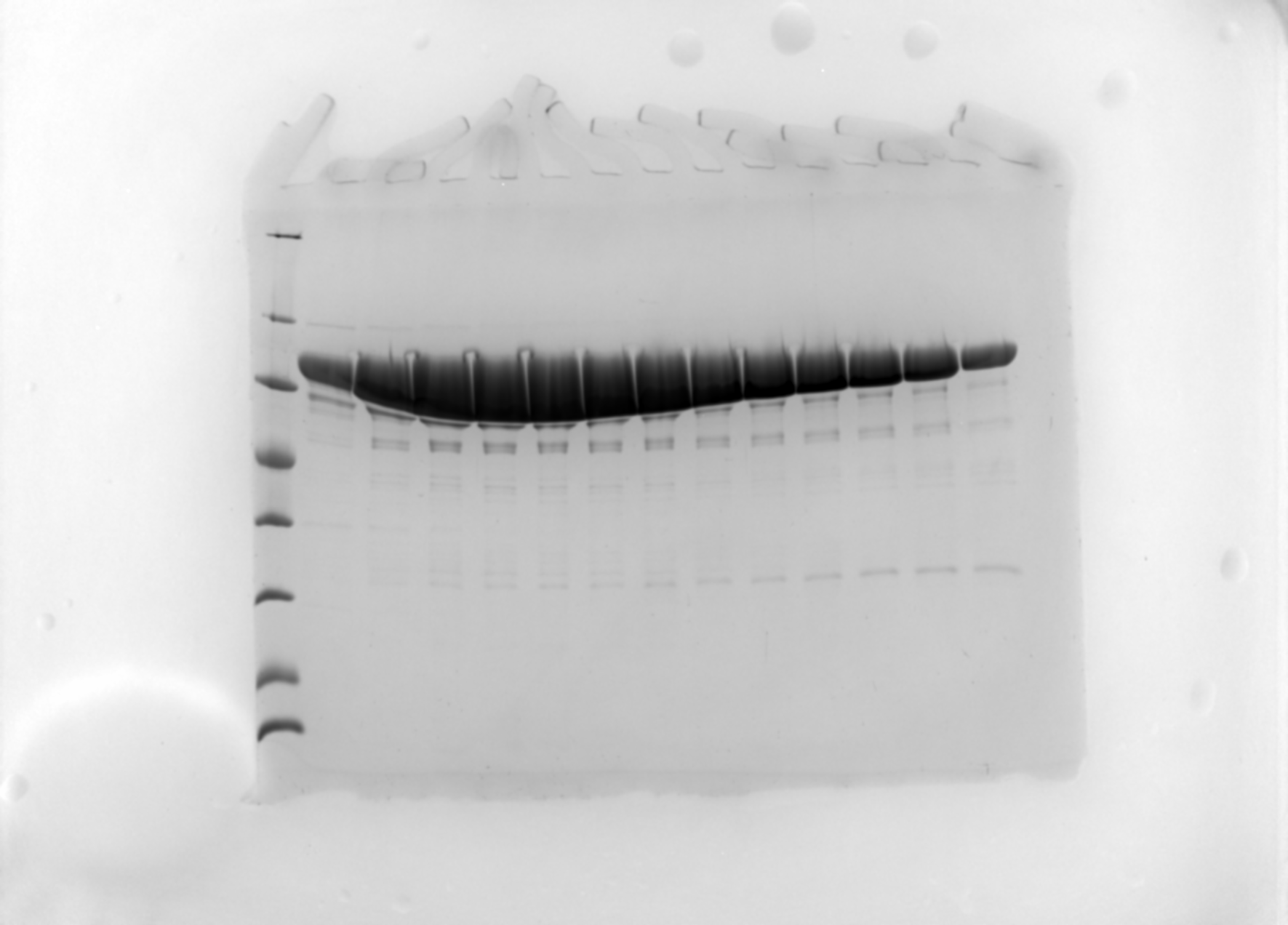

Supplement: Figure 3—figure supplement 1—source data 2. [file elife-104906-fig3-figsupp1-data2.zip › Figure 3-figure supplement 1-source data 2/Figure 3 - figure supplement 1 source data 2 Panel C- coomassie 1.tif]

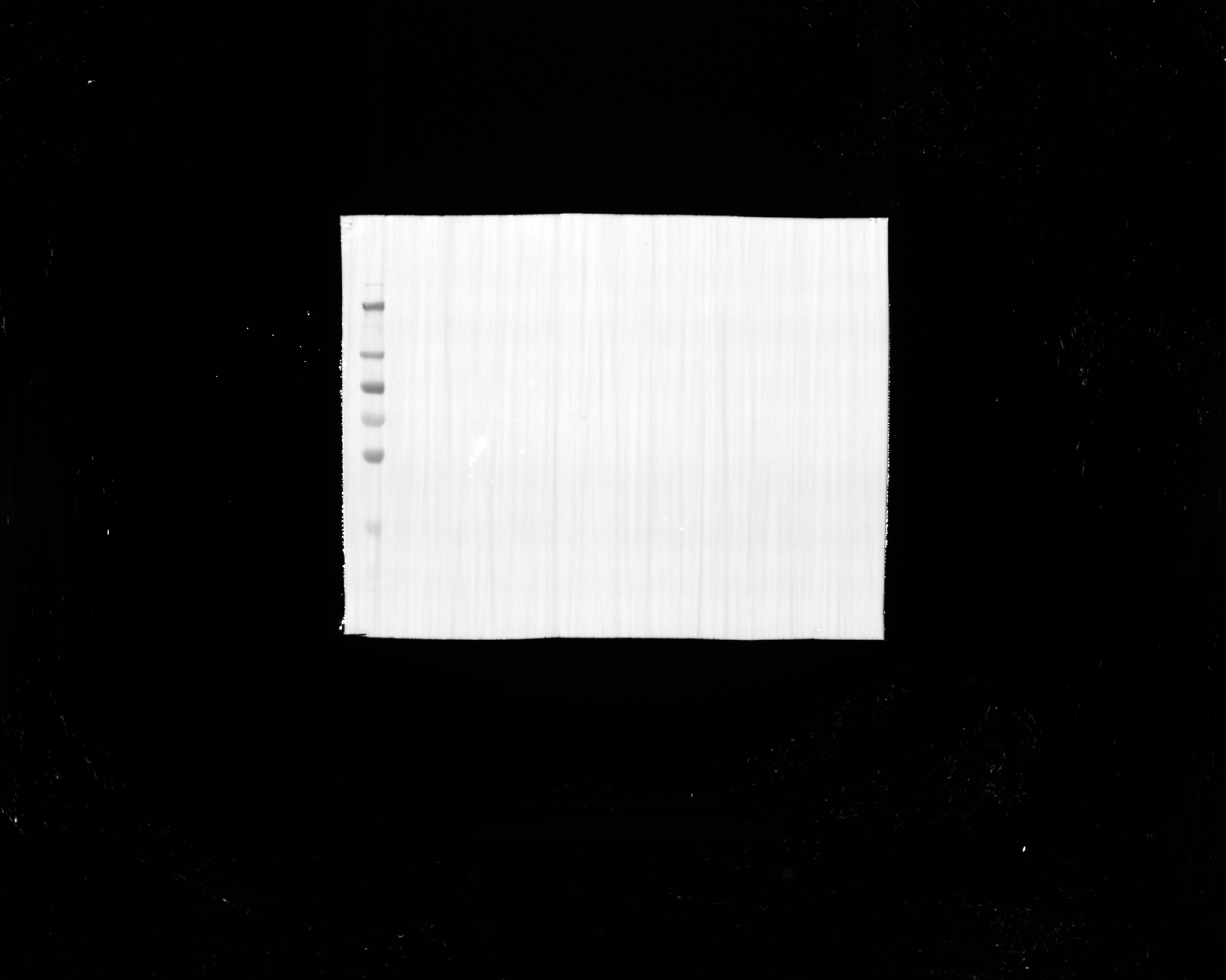

Supplement: Figure 3—figure supplement 1—source data 2. [file elife-104906-fig3-figsupp1-data2.zip › Figure 3-figure supplement 1-source data 2/Figure 3 - figure supplement 1 Source data 2 Panel B- blot 2.tif]

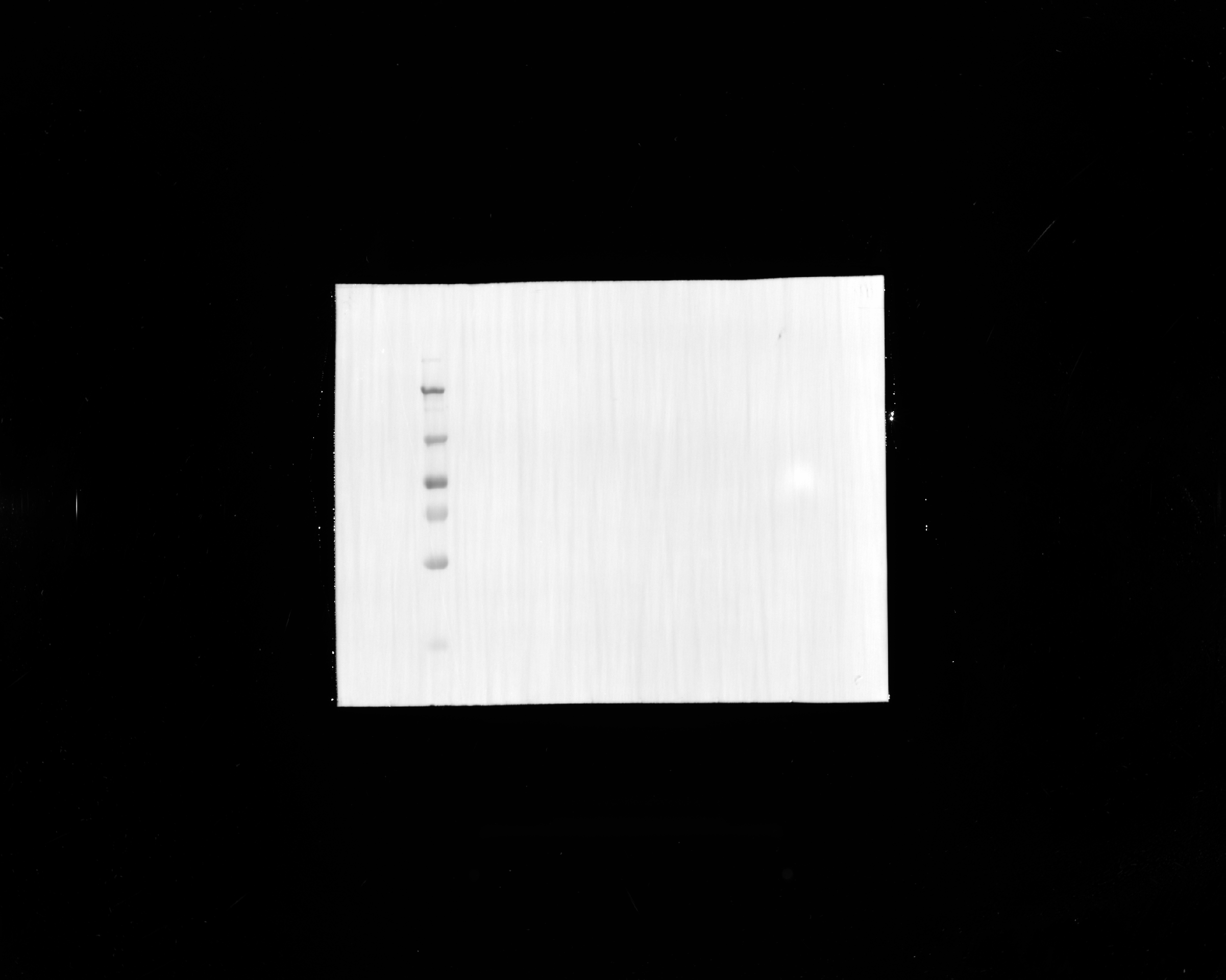

Supplement: Figure 3—figure supplement 1—source data 2. [file elife-104906-fig3-figsupp1-data2.zip › Figure 3-figure supplement 1-source data 2/Figure 3 - figure supplement 1 Source data 2 Panel B- blot 1.tif]

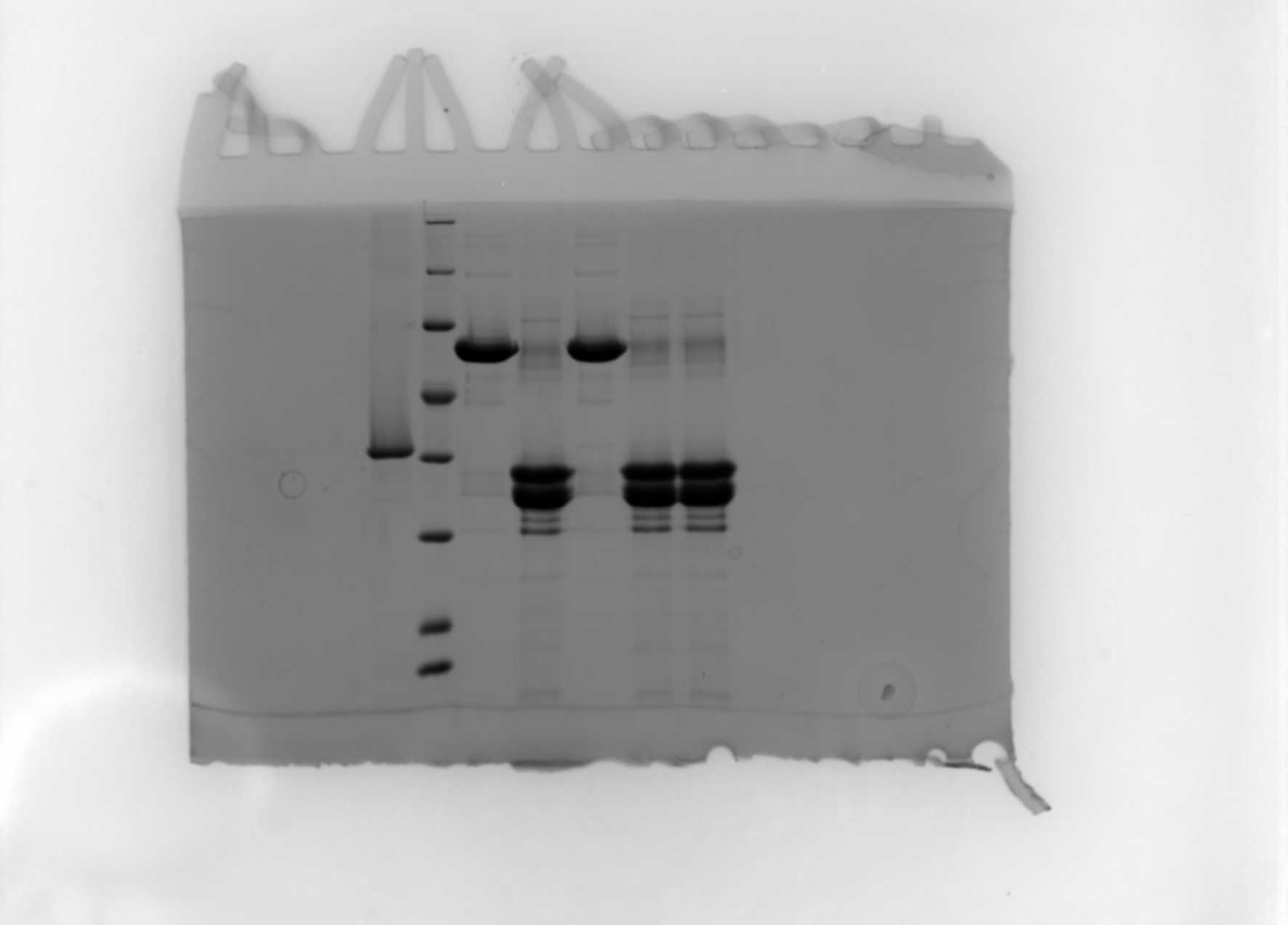

Supplement: Figure 4—source data 2. [file elife-104906-fig4-data2.zip › Figure 4-source data 2/Figure 4 source data 2 Panel D - coomassie.tif]

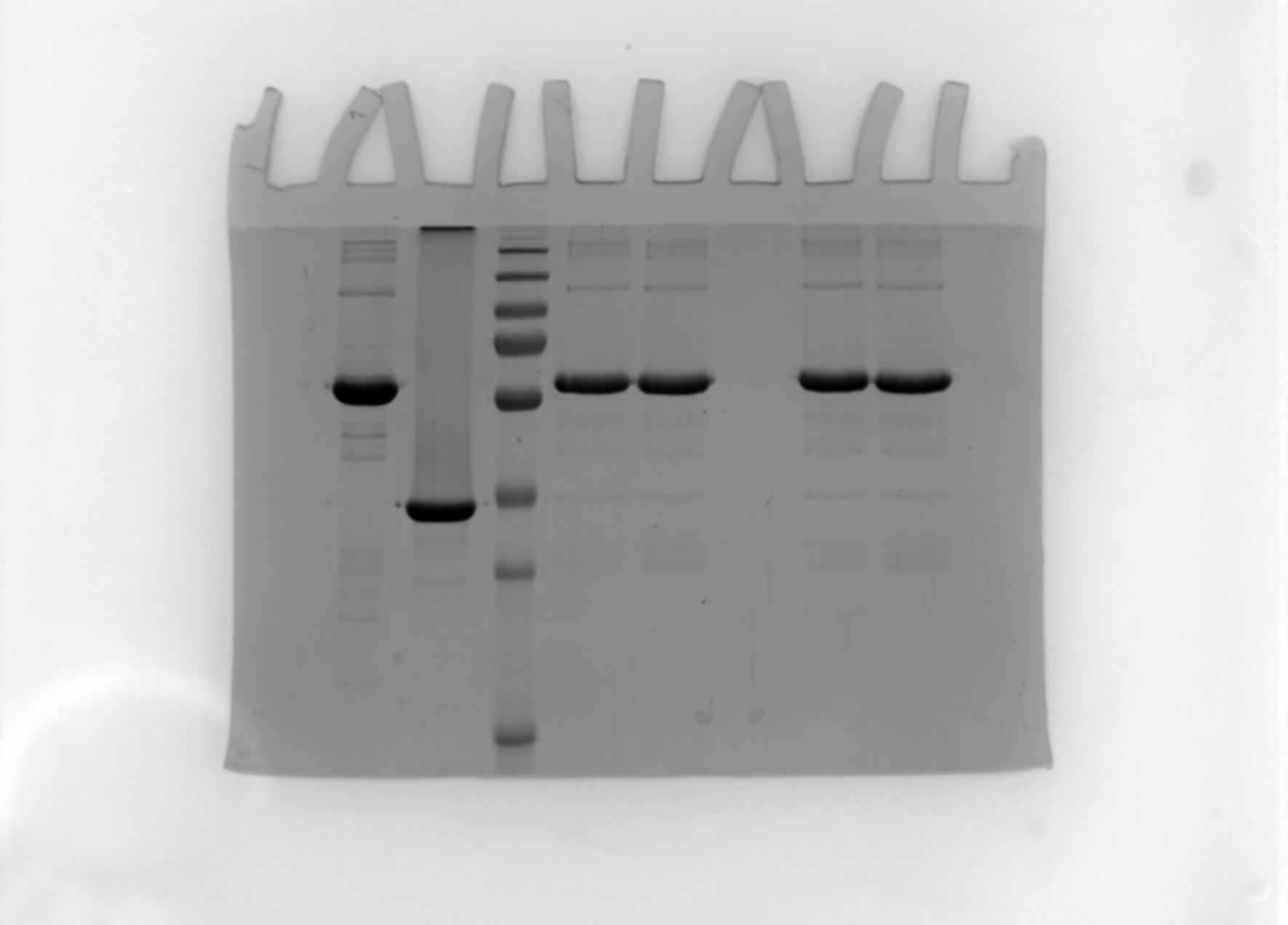

Supplement: Figure 4—source data 2. [file elife-104906-fig4-data2.zip › Figure 4-source data 2/Figure 4 source data 2 Panel C- coomassie.tif]

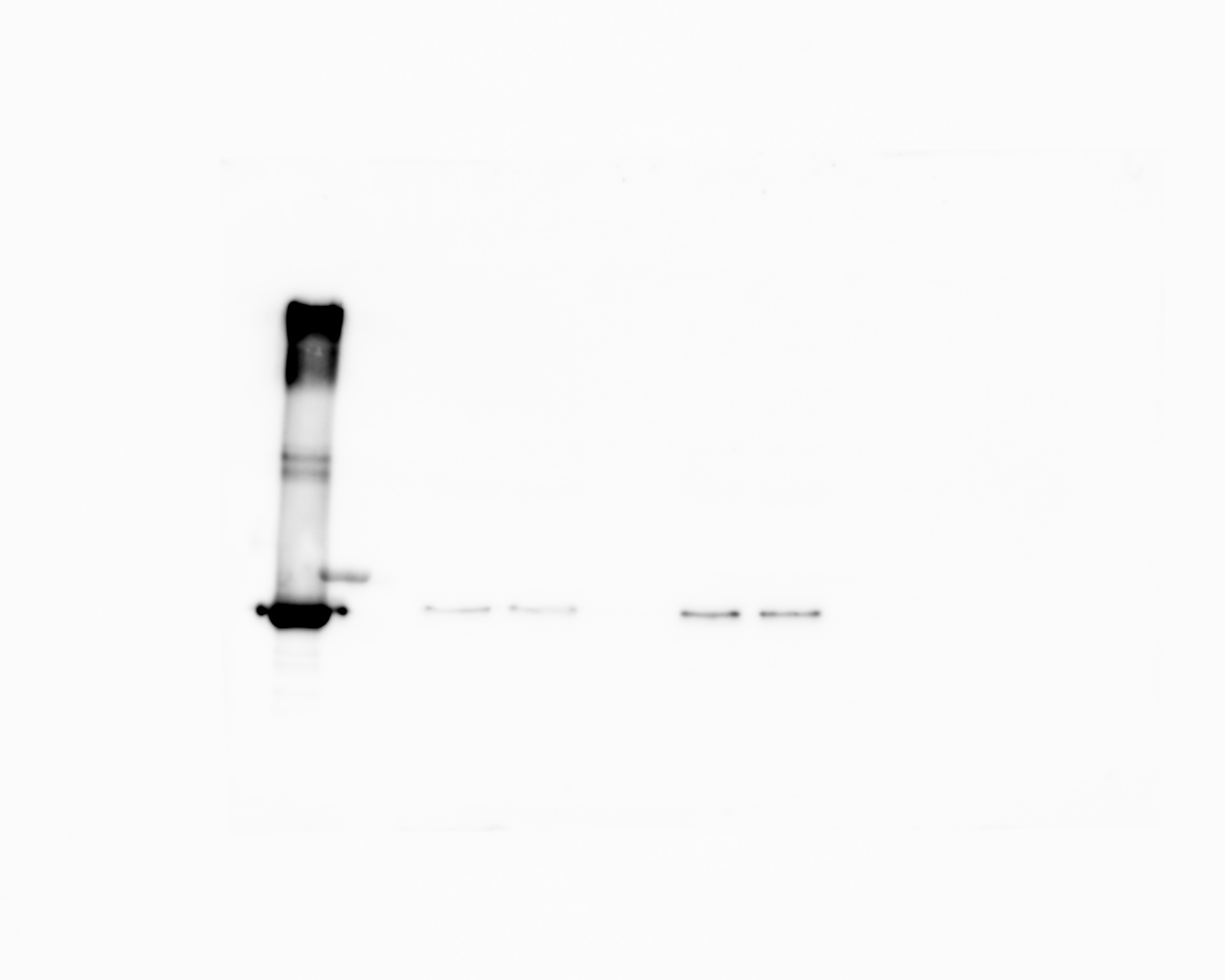

Supplement: Figure 4—source data 2. [file elife-104906-fig4-data2.zip › Figure 4-source data 2/Figure 4 source data 2 Panel C- ubiquitin.tif]

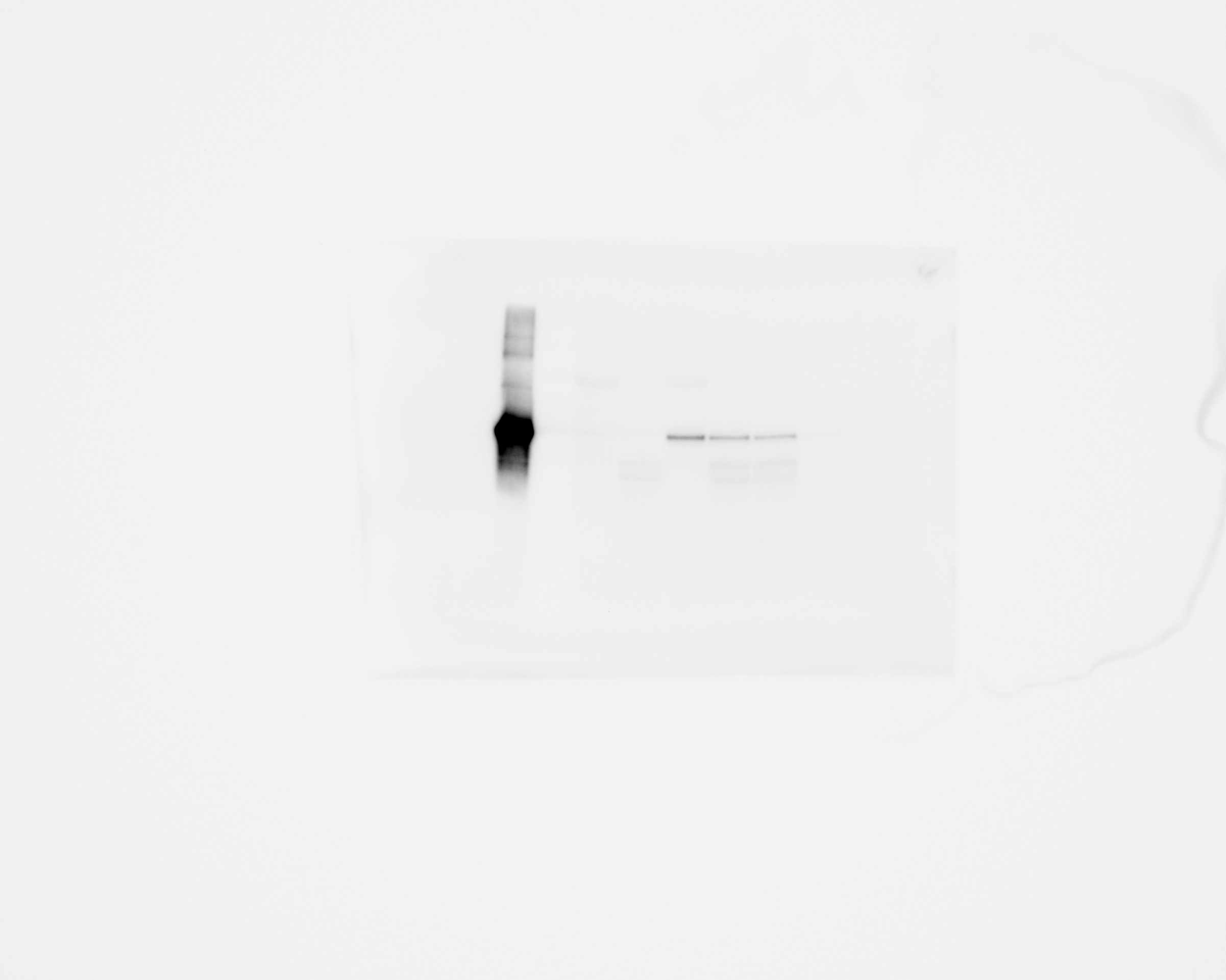

Supplement: Figure 4—source data 2. [file elife-104906-fig4-data2.zip › Figure 4-source data 2/Figure 4 source data 2 Panel D- ubiquitin.tif]

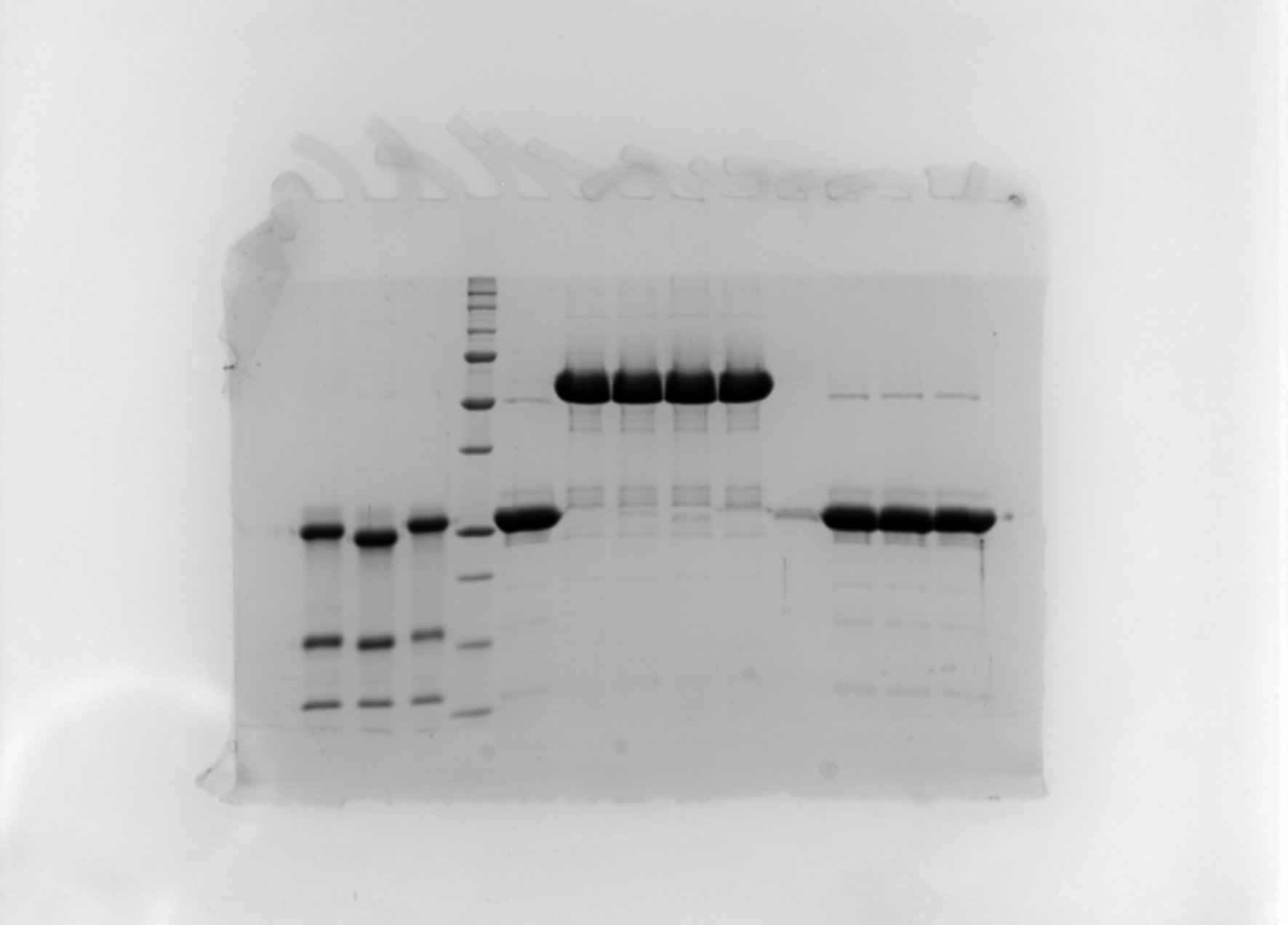

Supplement: Figure 4—source data 2. [file elife-104906-fig4-data2.zip › Figure 4-source data 2/Figure 4 source data 2 Panel A - coomassie.tif]

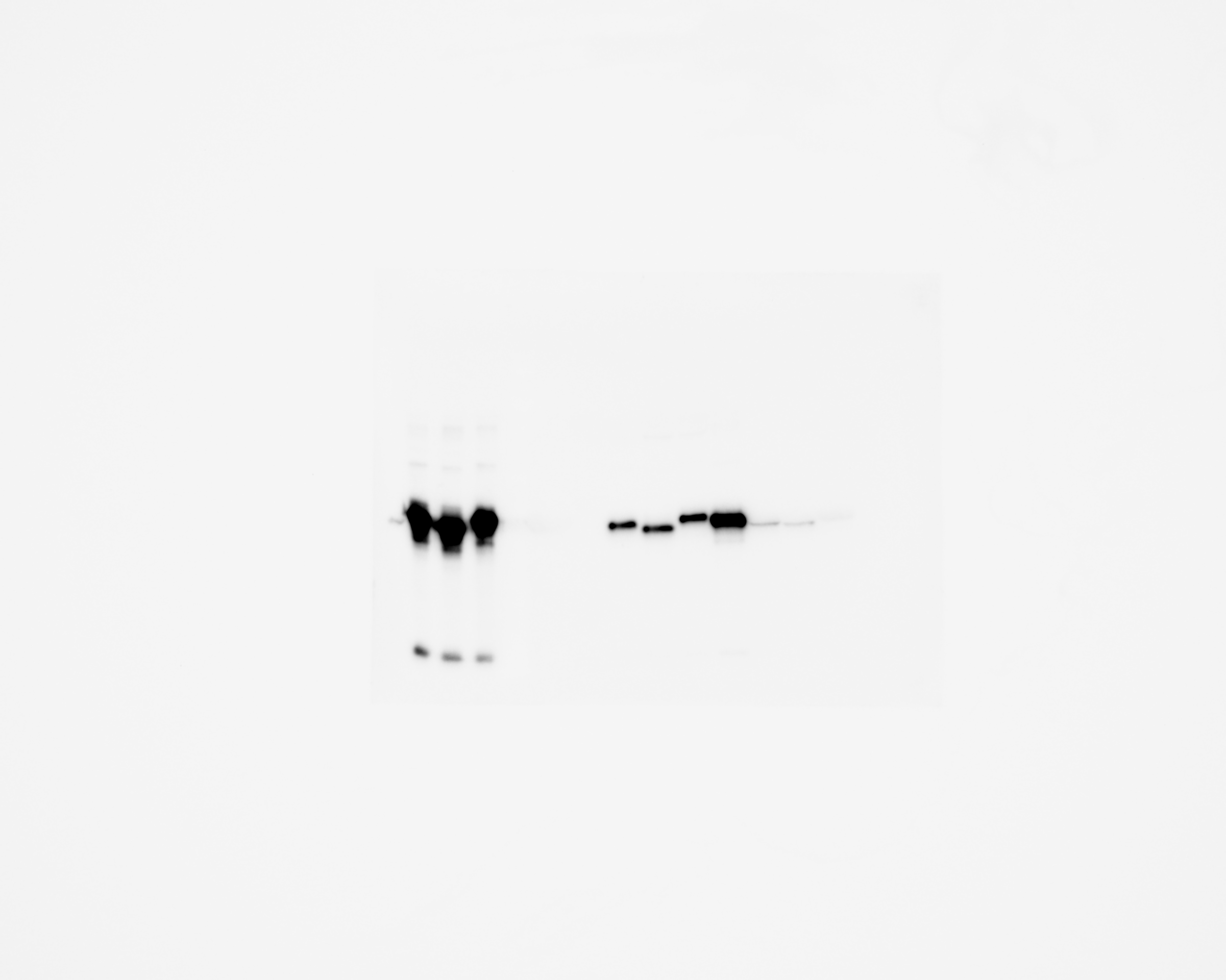

Supplement: Figure 4—source data 2. [file elife-104906-fig4-data2.zip › Figure 4-source data 2/Figure 4 source data 2 Panel A- ubiquitin.tif]

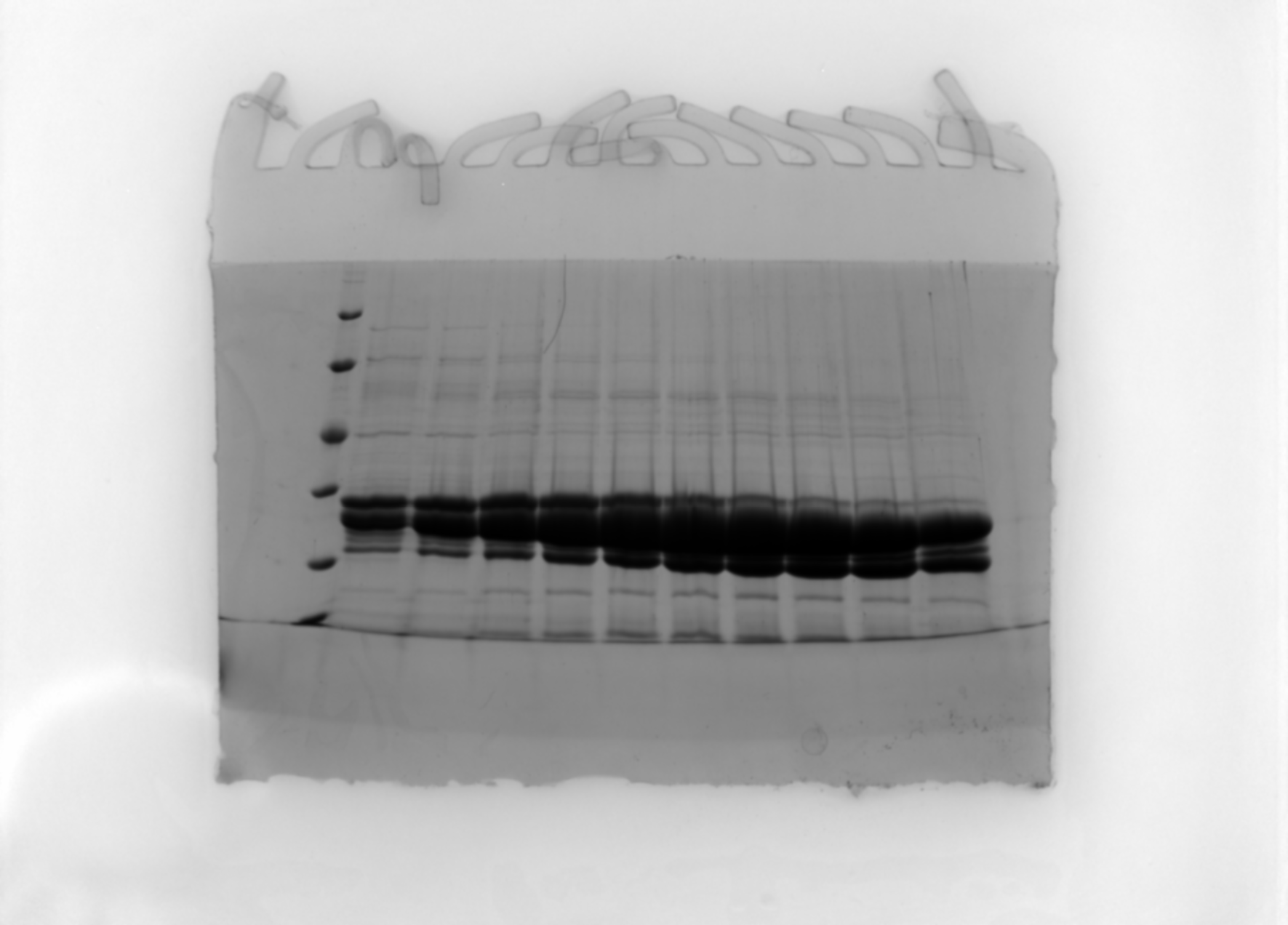

Supplement: Figure 4—figure supplement 1—source data 2. [file elife-104906-fig4-figsupp1-data2.zip › Figure 4-figure supplement 1-source data 2/Figure 4- figure supplement 1 source data 2 Panel B- coomassie 2.tif]

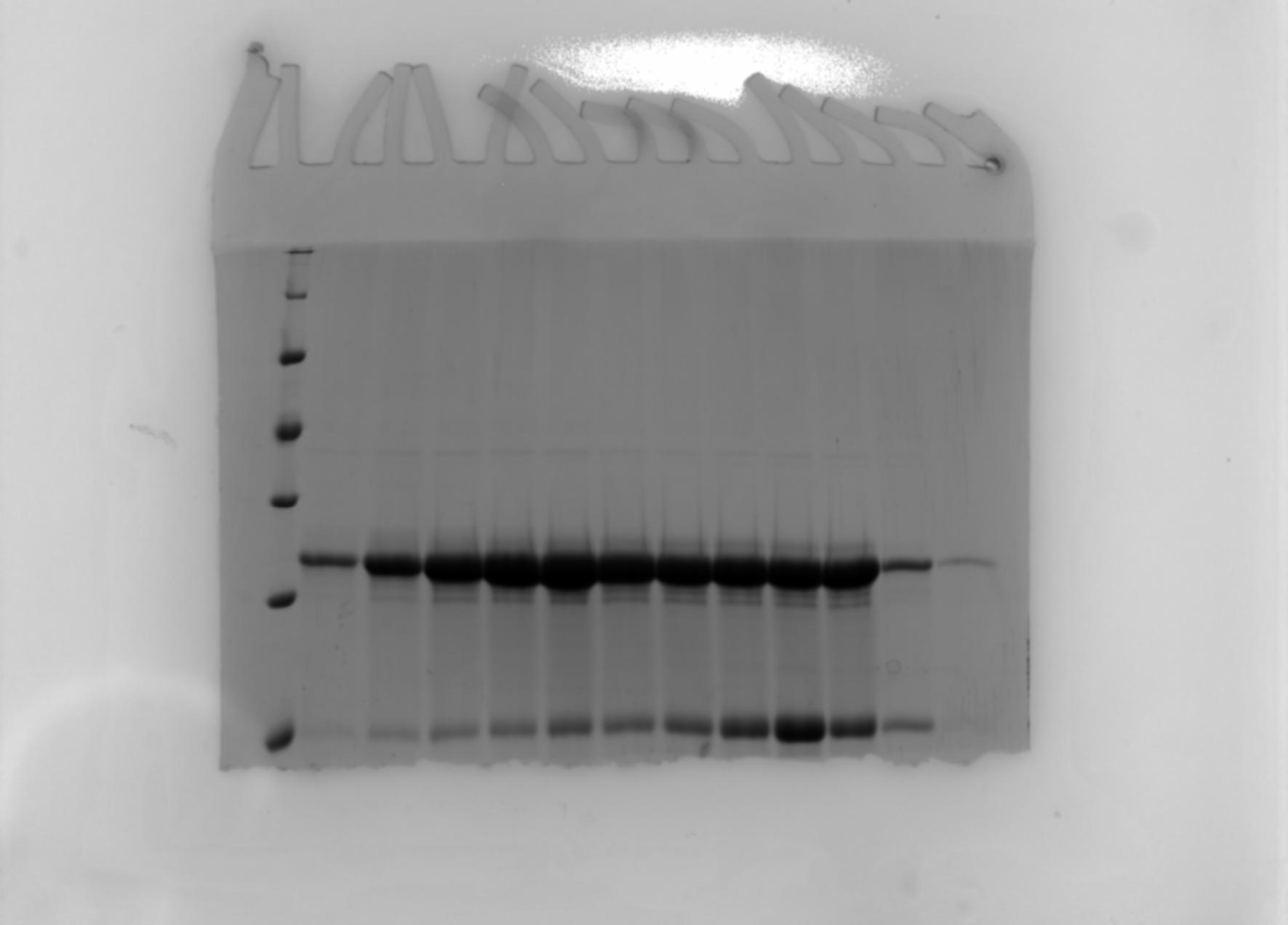

Supplement: Figure 4—figure supplement 1—source data 2. [file elife-104906-fig4-figsupp1-data2.zip › Figure 4-figure supplement 1-source data 2/Figure 4- figure supplement 1 source data 2 Panel C- coomassie 3.tif]

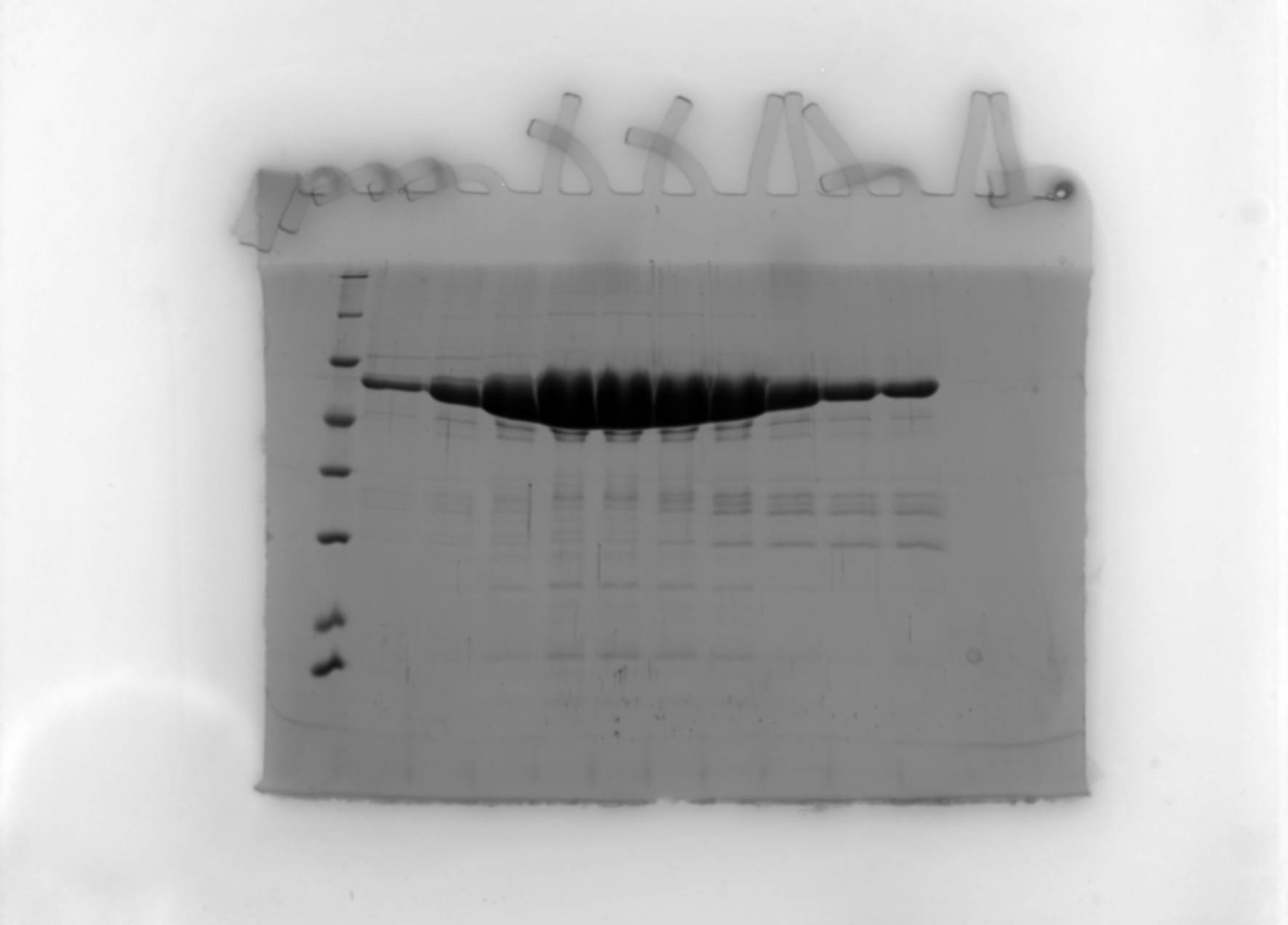

Supplement: Figure 4—figure supplement 1—source data 2. [file elife-104906-fig4-figsupp1-data2.zip › Figure 4-figure supplement 1-source data 2/Figure 4- figure supplement 1 source data 2 Panel A- coomassie 1.tif]

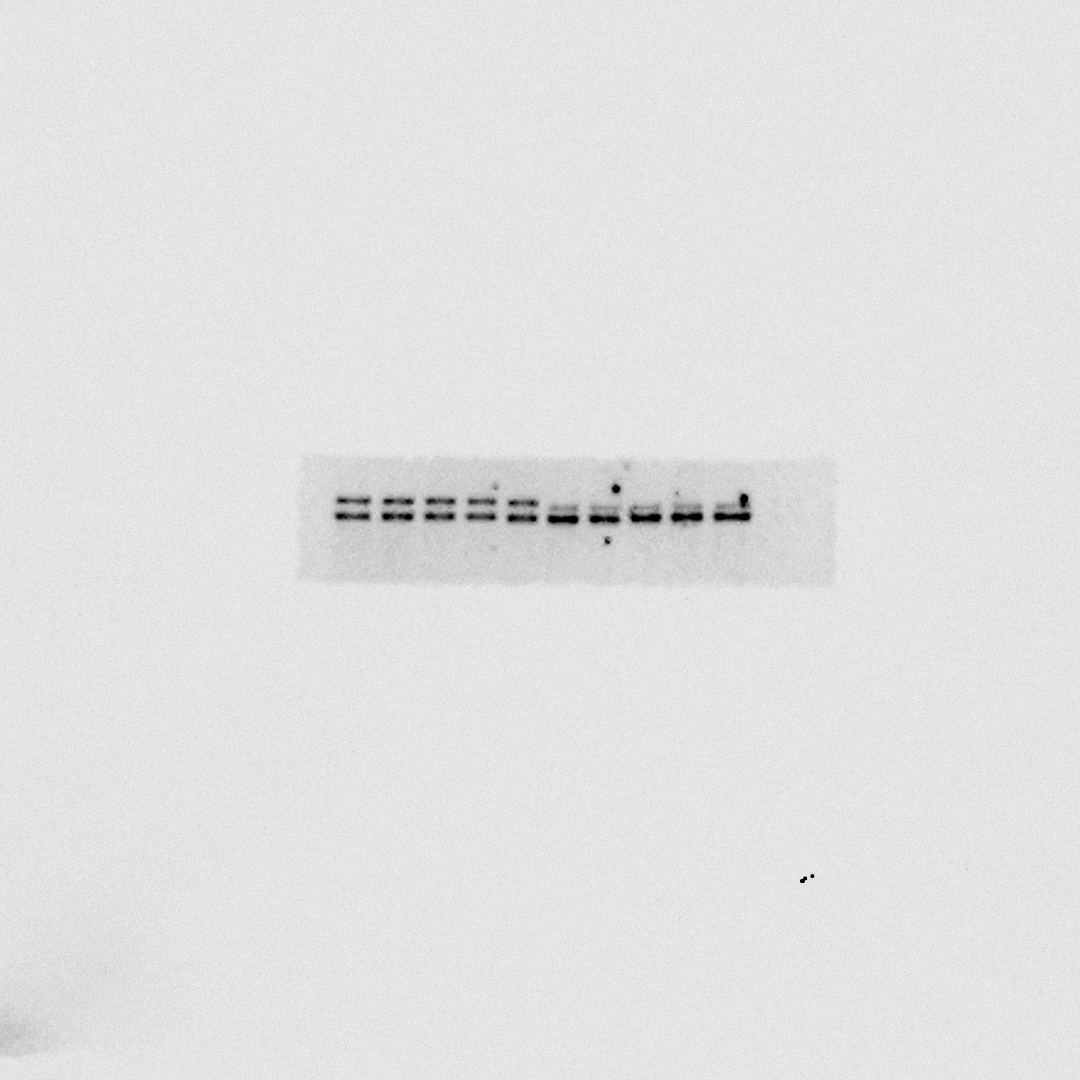

Supplement: Figure 5—source data 2. [file elife-104906-fig5-data2.zip › Figure 5-source data 2/Figure 5 source data 2 Panel D_IFT172.Tif]

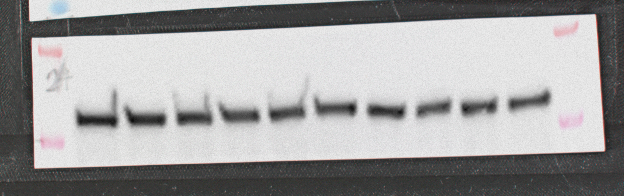

Supplement: Figure 5—source data 2. [file elife-104906-fig5-data2.zip › Figure 5-source data 2/Figure source data 2_Panel E_AKT.tif]

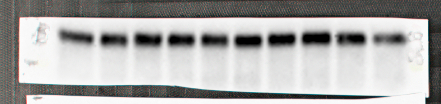

Supplement: Figure 5—source data 2. [file elife-104906-fig5-data2.zip › Figure 5-source data 2/Figure source data 2 Panel E_SMAD2.tif]

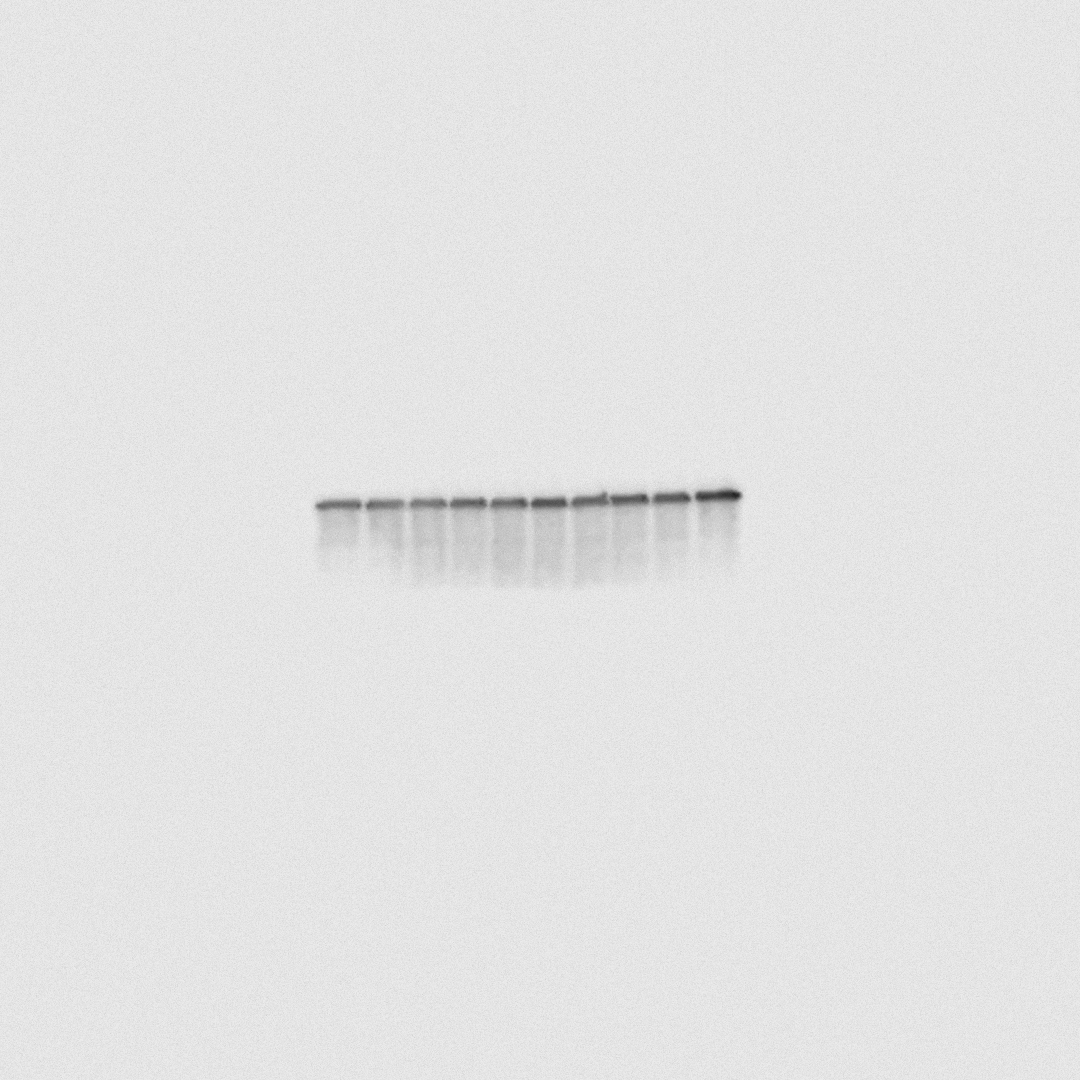

Supplement: Figure 5—source data 2. [file elife-104906-fig5-data2.zip › Figure 5-source data 2/Figure 5 source data 2 Panel D_GAPDHTif.Tif]

Figure 5-figure supplement 2-source data 1

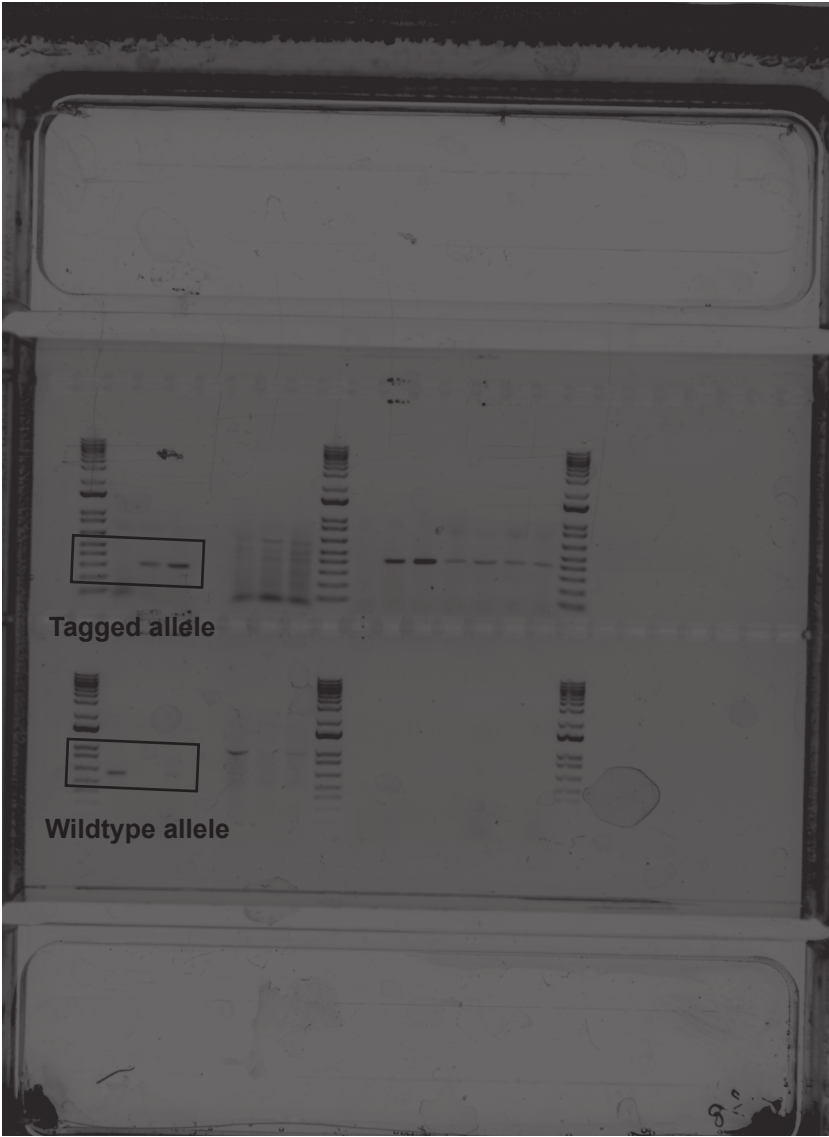

Supplement: Figure 5—figure supplement 2—source data 1. [file elife-104906-fig5-figsupp2-data1.zip › Figure 5-figure supplement 2-source data 1/Figure 5-figure supplement 2-source data 1.pdf]

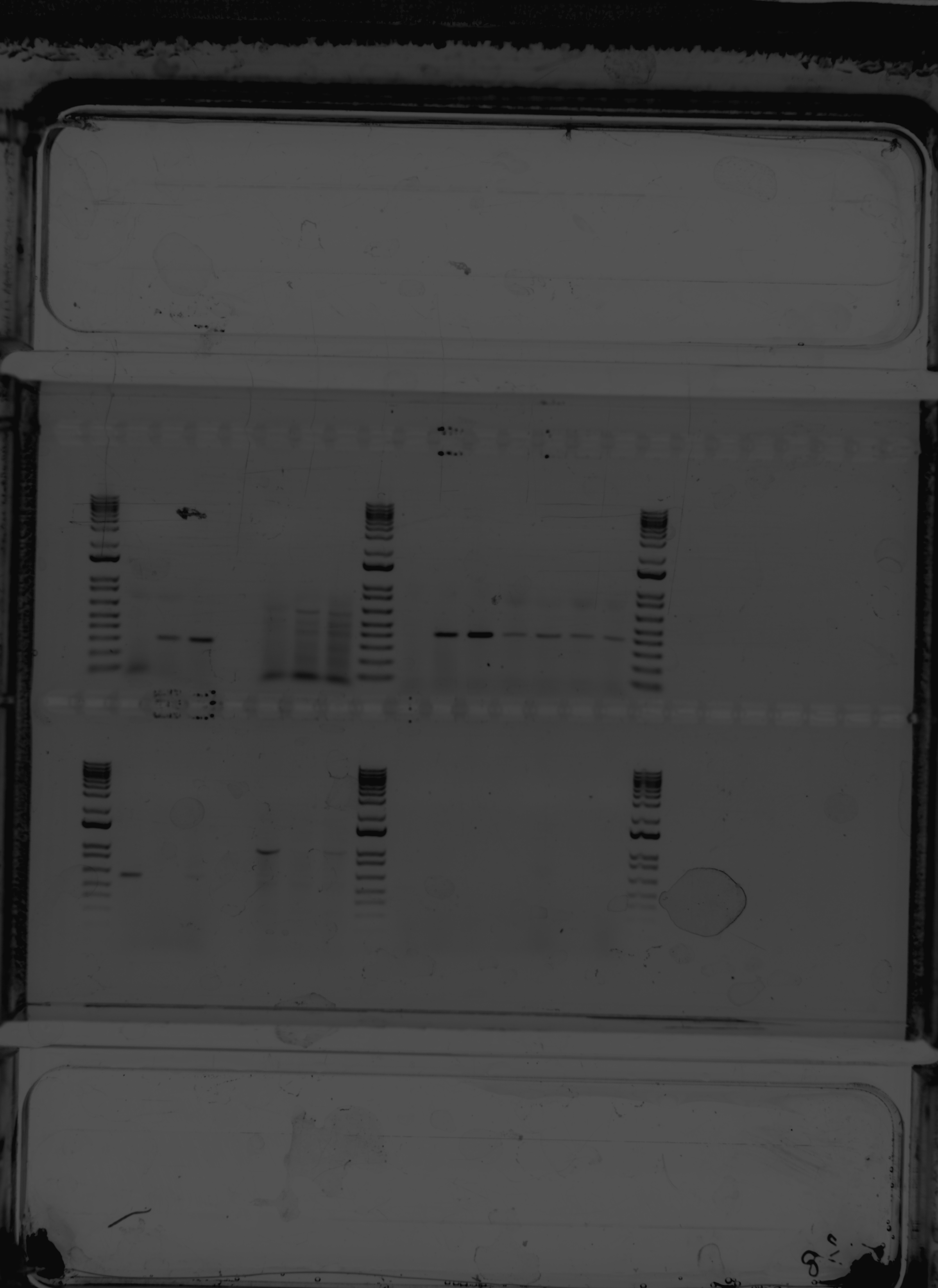

Supplement: Figure 5—figure supplement 2—source data 2. [file elife-104906-fig5-figsupp2-data2.zip › Figure 5-figure supplement 2-source data 2/230213_PCR-IFT172eGclones 20230213_180752_Fl_UV.tif]

Figure 5-figure supplement 3-source data 1

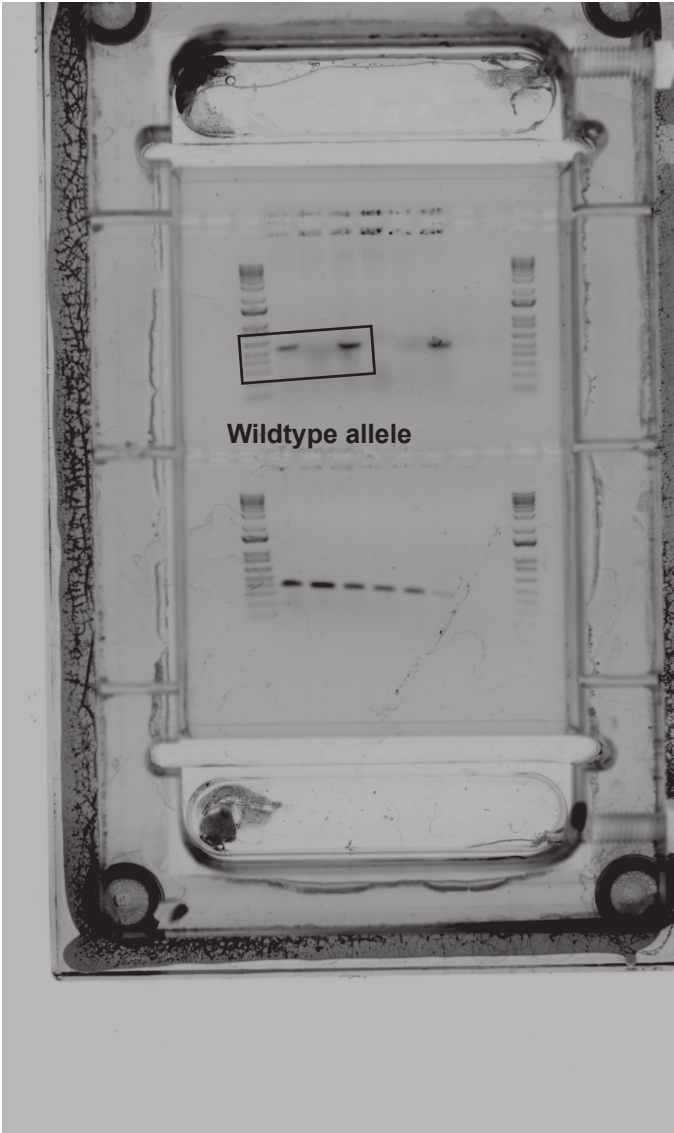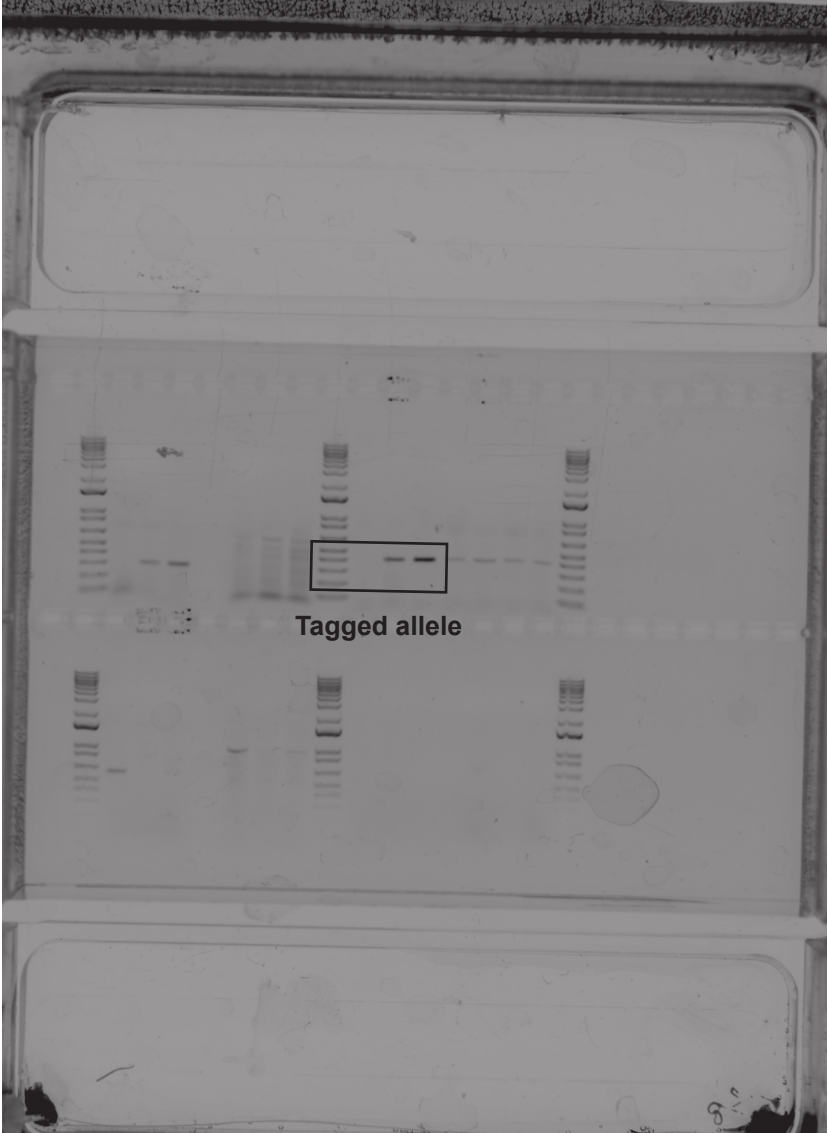

Supplement: Figure 5—figure supplement 3—source data 1. [file elife-104906-fig5-figsupp3-data1.zip › Figure 5-figure supplement 3-source data 1/Figure 5-figure supplement 3-source data 1.pdf]

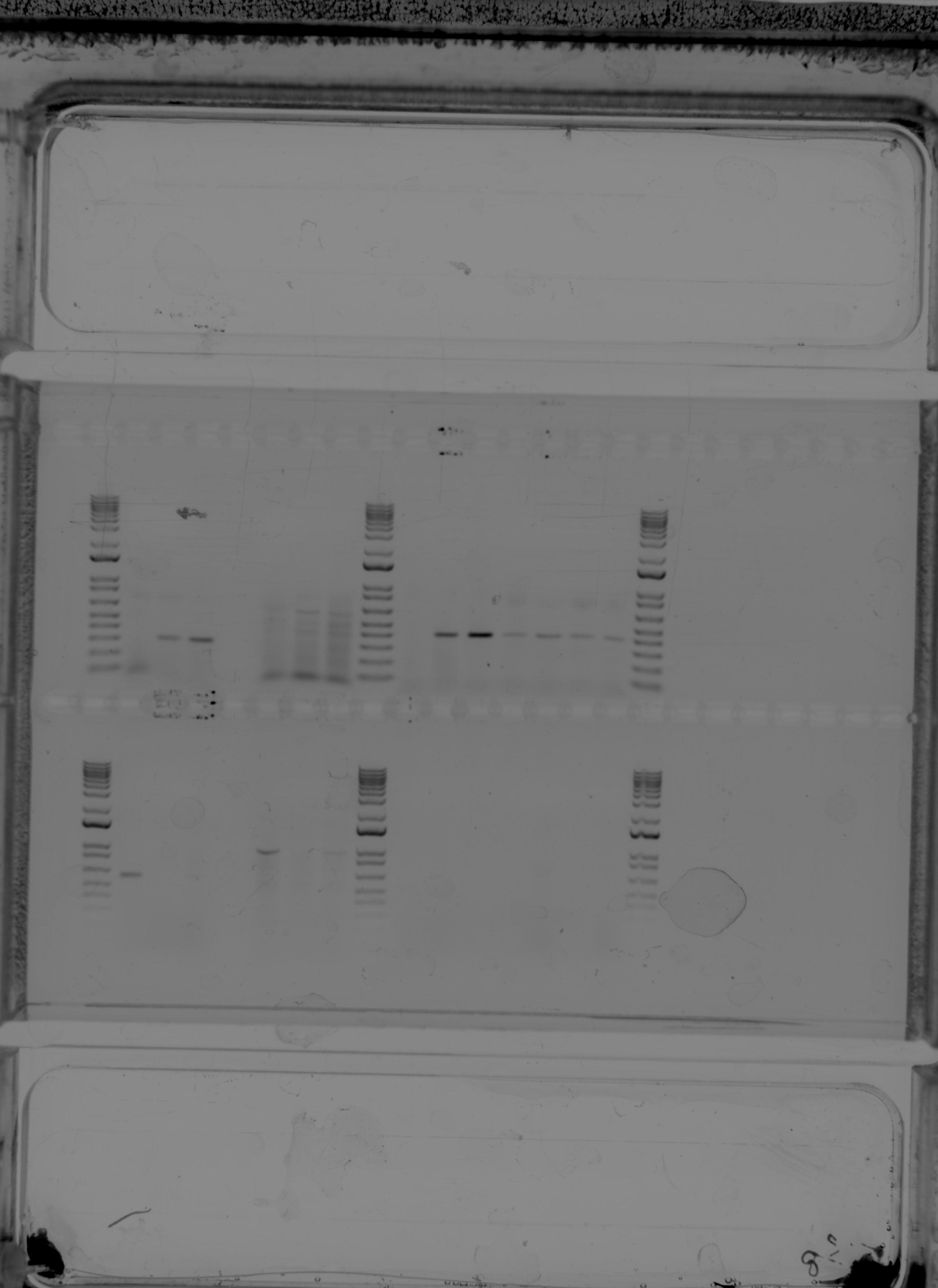

Supplement: Figure 5—figure supplement 3—source data 2. [file elife-104906-fig5-figsupp3-data2.zip › Figure 5-figure supplement 3-source data 2/230213_PCR-IFT172eGclones 20230213_181128_Fl_UV.tif]

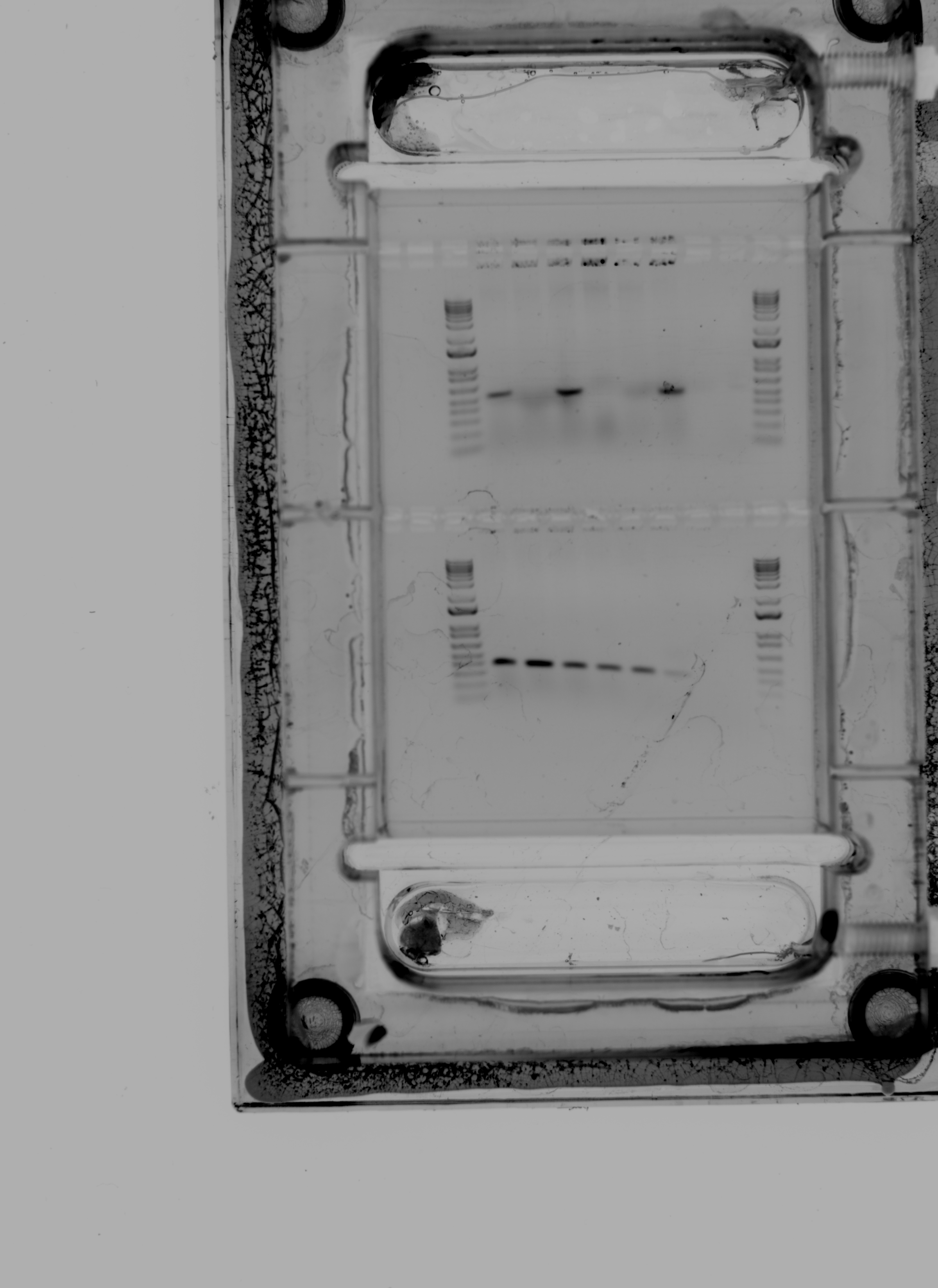

Supplement: Figure 5—figure supplement 3—source data 2. [file elife-104906-fig5-figsupp3-data2.zip › Figure 5-figure supplement 3-source data 2/230216_PCR-IFTclones 20230216_180908_Fl_UV.tif]

Figure 5-figure supplement 4-source data 1

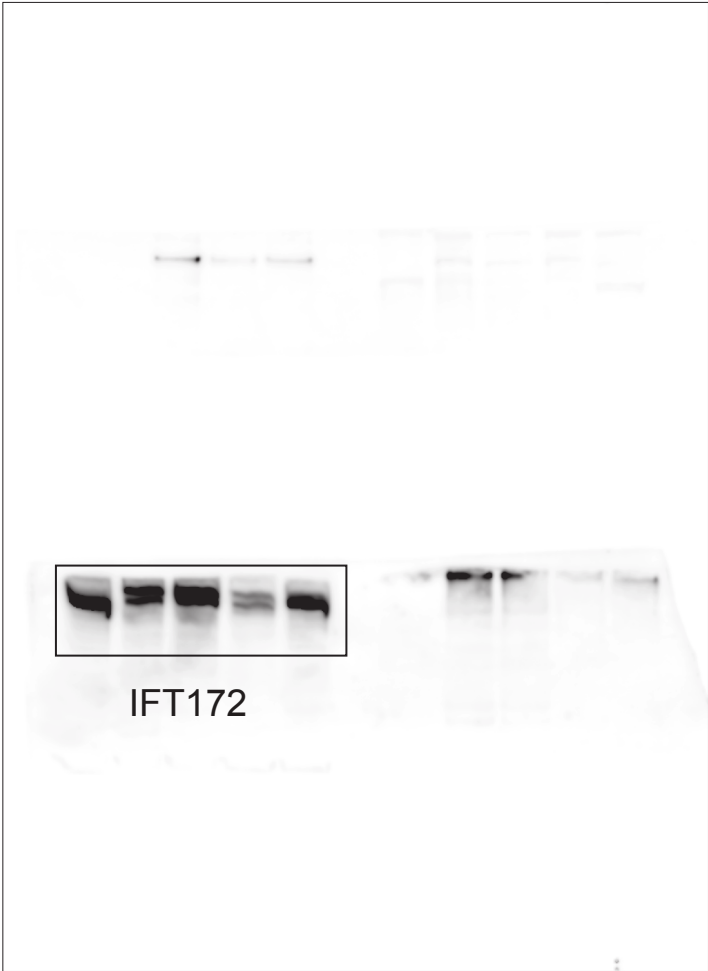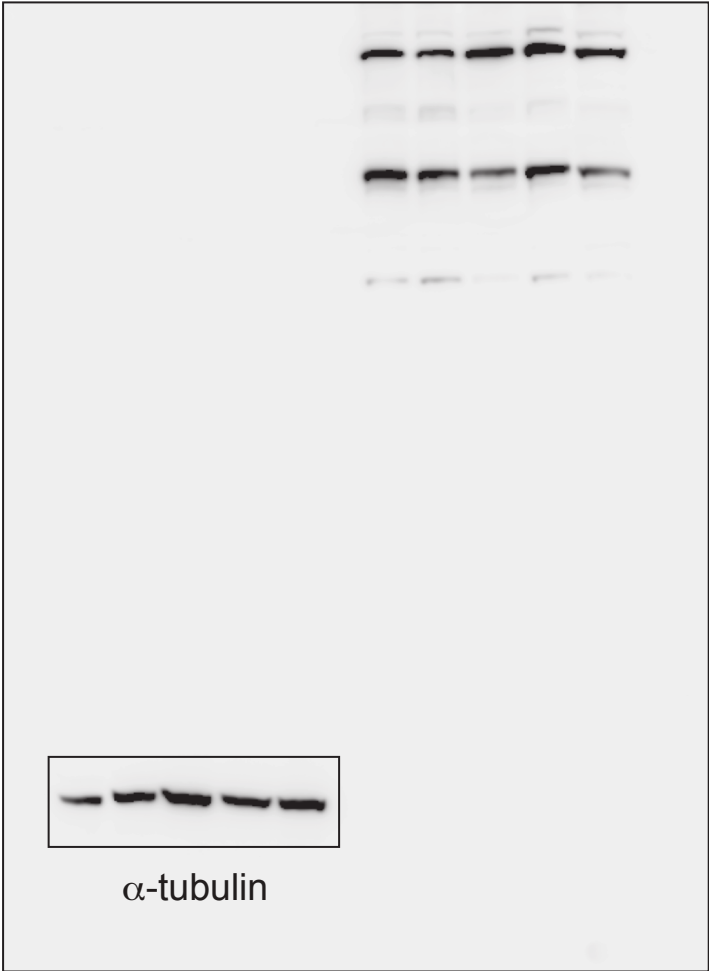

Supplement: Figure 5—figure supplement 4—source data 1. [file elife-104906-fig5-figsupp4-data1.zip › Figure 5-figure supplement 4-source data 1/Figure 5-figure supplement 4-source data 1.pdf]

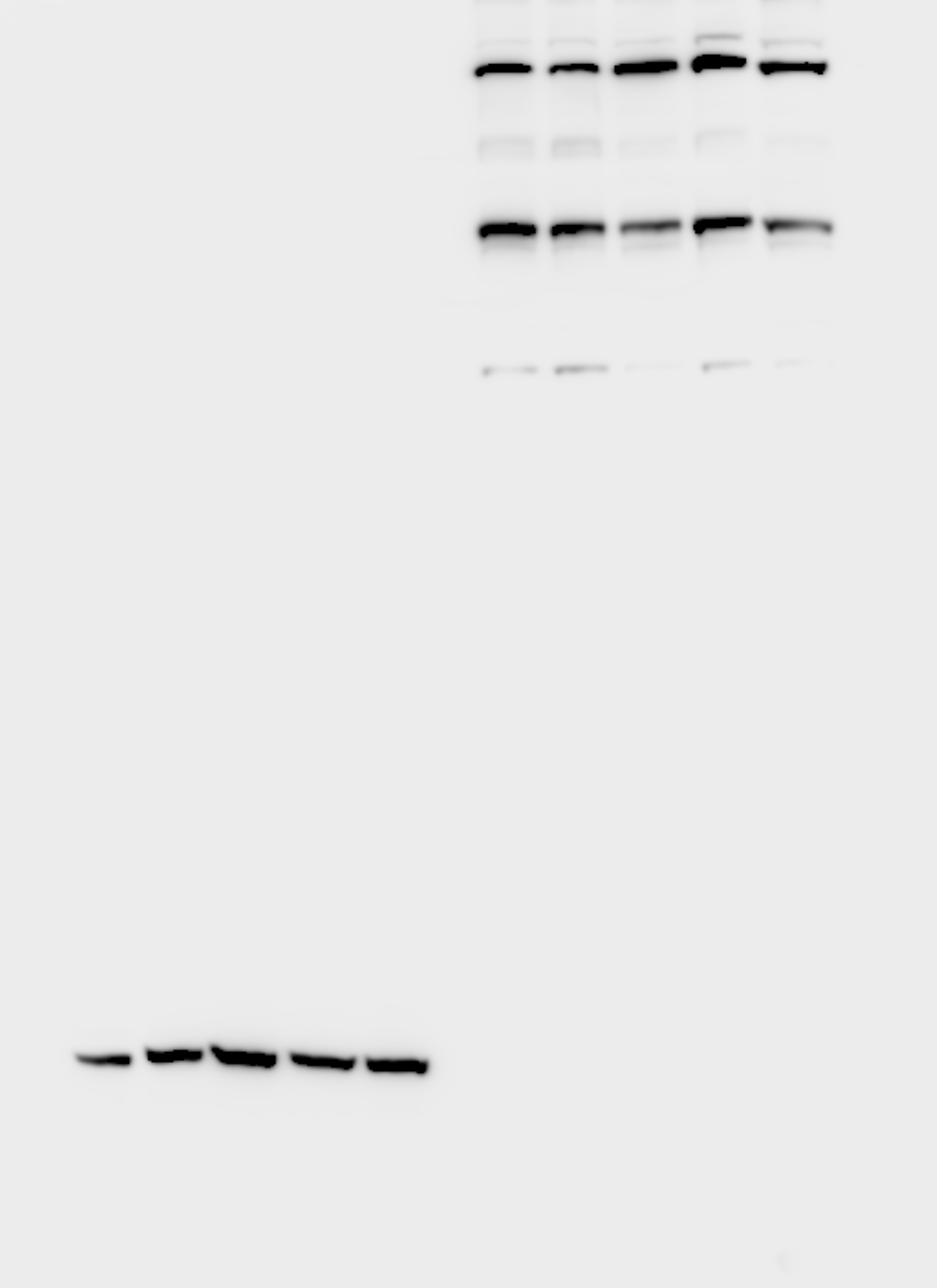

Supplement: Figure 5—figure supplement 4—source data 2. [file elife-104906-fig5-figsupp4-data2.zip › Figure 5-figure supplement 4-source data 2/2022.02.01_18.45.05_Ch.tif]

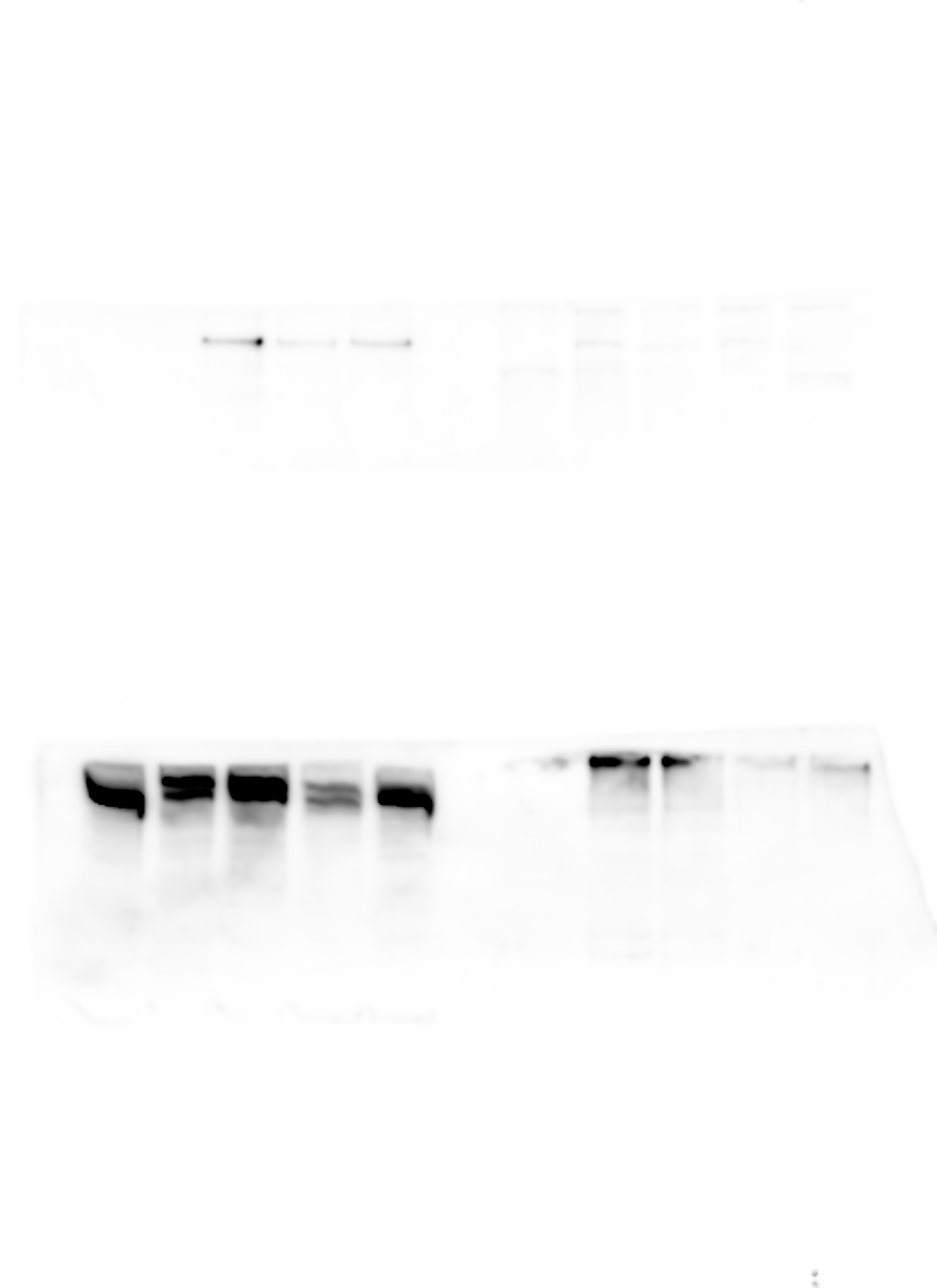

Supplement: Figure 5—figure supplement 4—source data 2. [file elife-104906-fig5-figsupp4-data2.zip › Figure 5-figure supplement 4-source data 2/2022.02.03_18.00.38_Ch.tif]
