# Supplementary material for: Intraflagellar transport protein IFT172 contains a C-terminal ubiquitin-binding U-box-like domain involved in ciliary signaling
Source: eLife. 2026 Jun 23;14:RP104906. doi: 10.7554/eLife.104906 (PMC13290226; doi:10.7554/eLife.104906)
Supplement: Figure 3—source data 1. [file elife-104906-fig3-data1.zip › Figure 3-source data 1/Fig 3 source data 1.pdf]

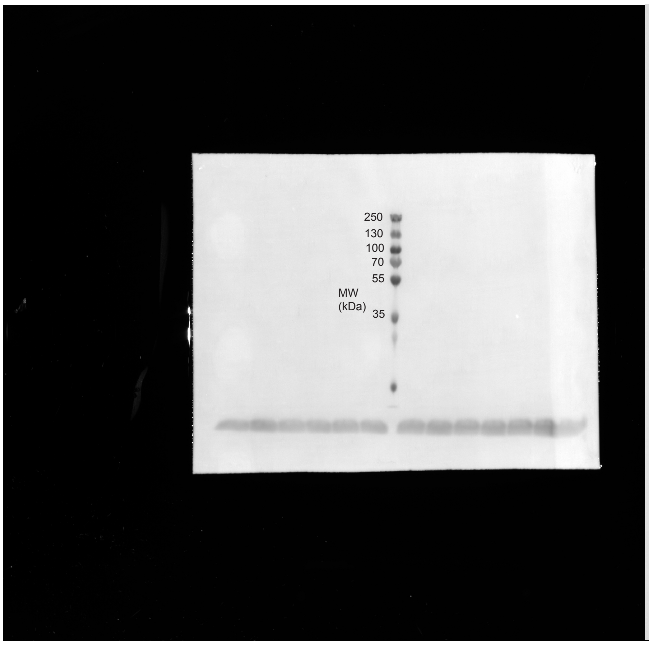

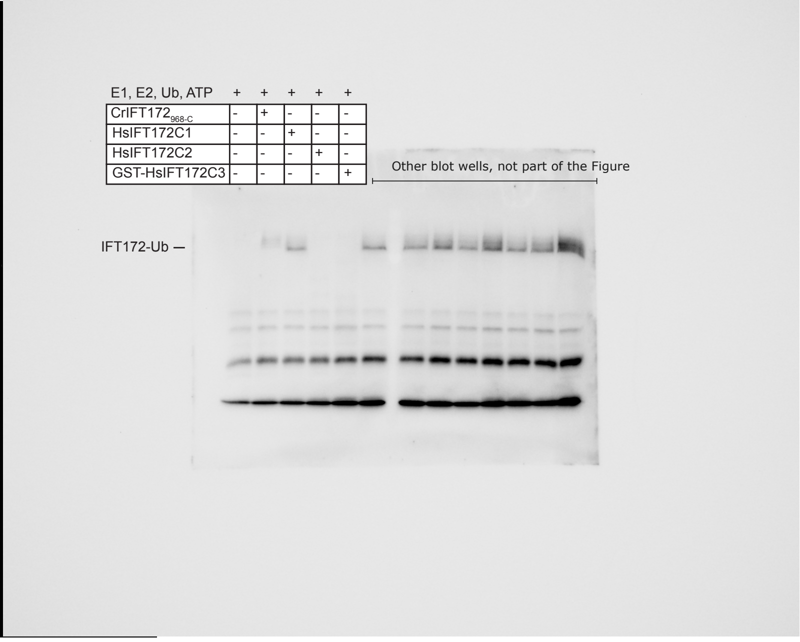

Blot image

α-Ubiquitin

Original blot images and α-Ubiquitin blots used to generate Figure 3, panel A, labelled according to the original figure panel.

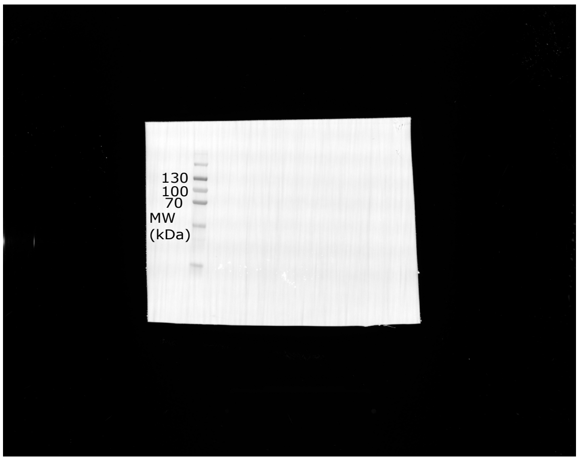

Blot image

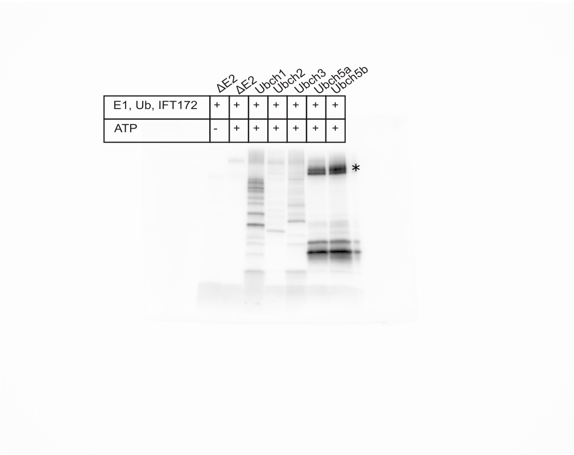

α-Ubiquitin

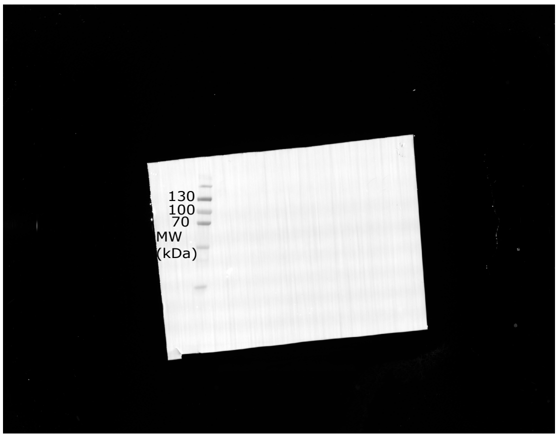

Blot image

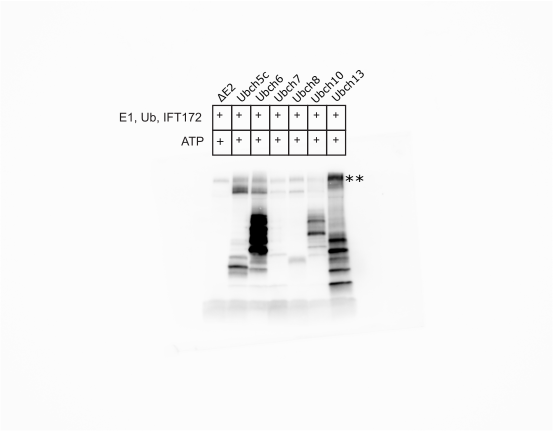

α-Ubiquitin

* Putative IFT172-Ub conjugates
** Ube1~Ub reaction intermediate

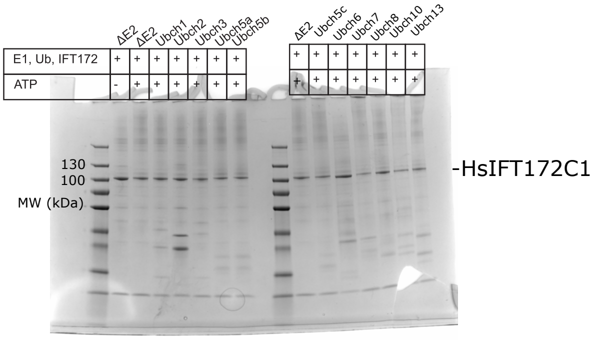

Coomassie

Original blot images, α-Ubiquitin blots and coomassie stainings used to generate Figure 3, panel B, labelled according to the original figure panel.

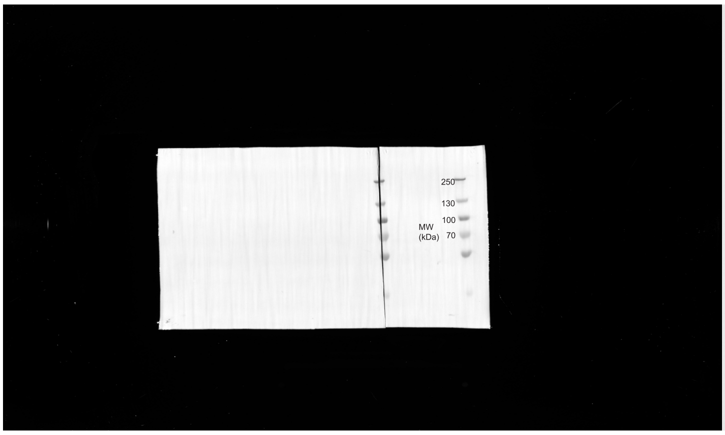

Blot image

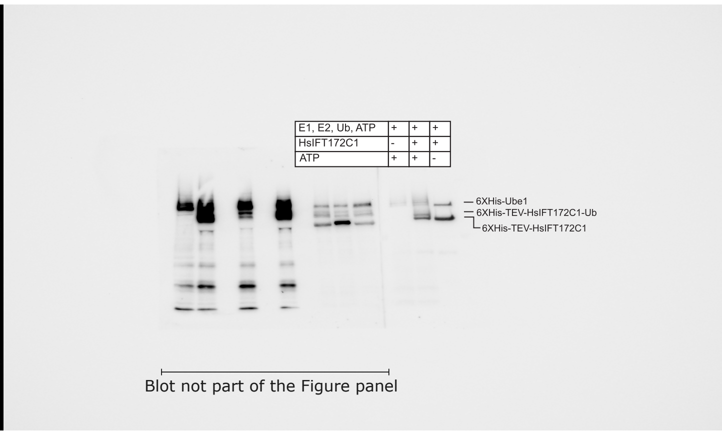

α-His

Original blot images and α-His blots used to generate Figure 3, panel C, labelled according to the original figure panel.

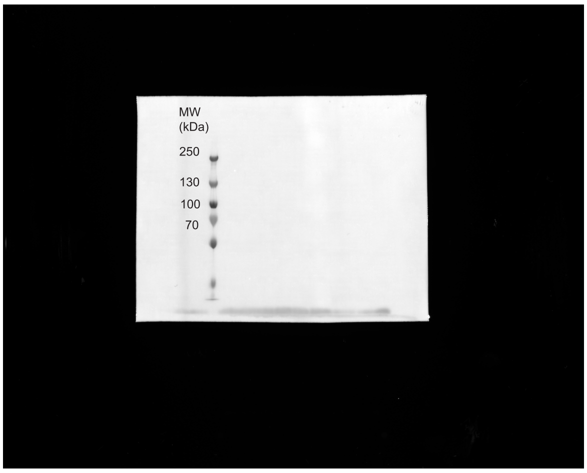

Blot image

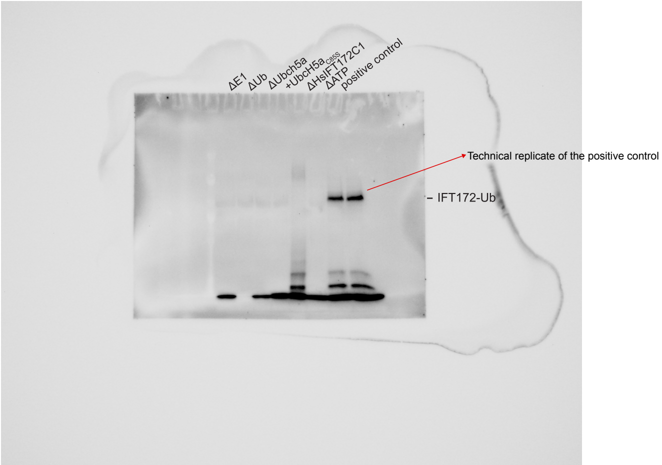

α-Ubiquitin

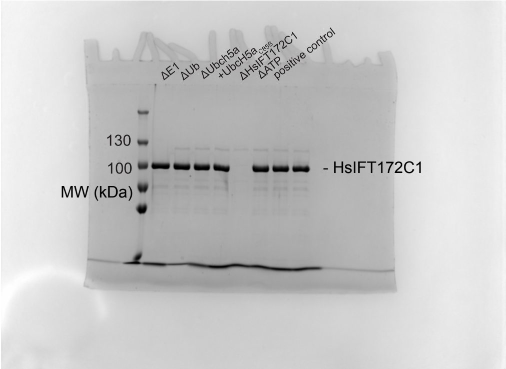

Coomassie

Original blot images, α-Ubiquitin blots and coomassie stainings used to generate Figure 3, panel D, labelled according to the original figure panel.

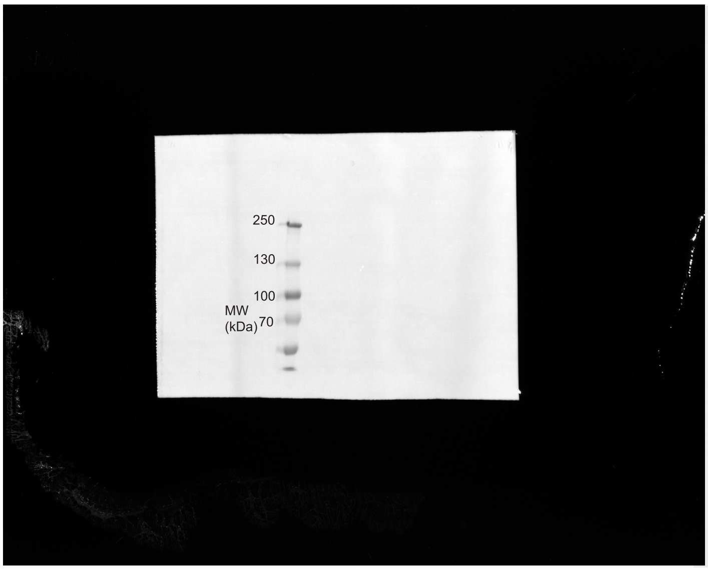

Blot image

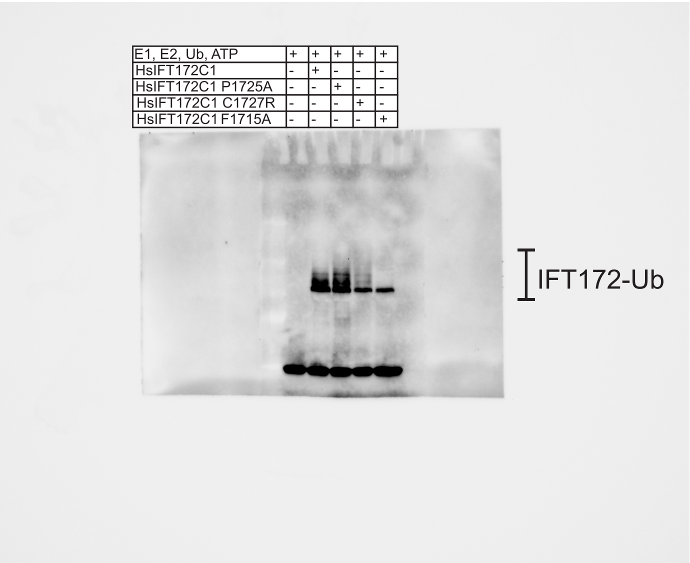

α-Ubiquitin

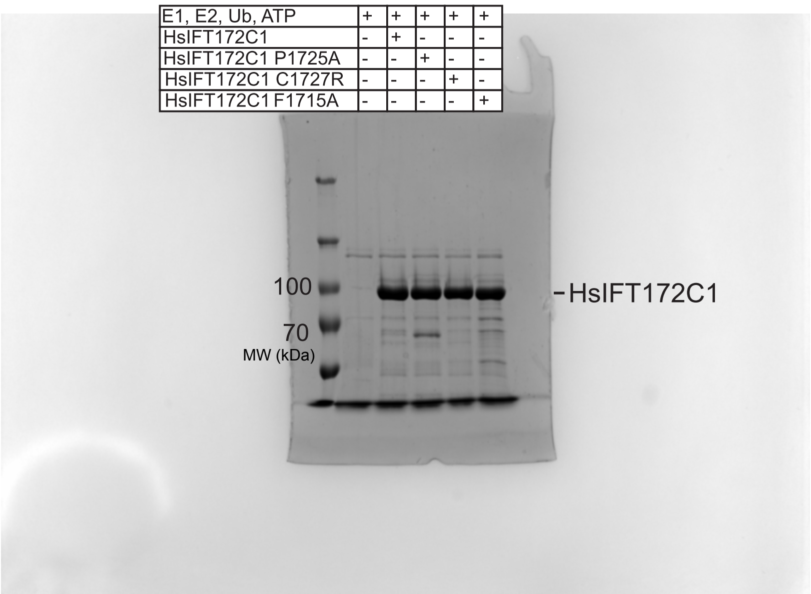

Coomassie

Original blot images, α-Ubiquitin blots and coomassie stainings used to generate Figure 3, panel F, labelled according to the original figure panel.
