# Supplementary material for: Intraflagellar transport protein IFT172 contains a C-terminal ubiquitin-binding U-box-like domain involved in ciliary signaling
Source: eLife. 2026 Jun 23;14:RP104906. doi: 10.7554/eLife.104906 (PMC13290226; doi:10.7554/eLife.104906)
Supplement: Figure 3—figure supplement 1—source data 1. [file elife-104906-fig3-figsupp1-data1.zip › Figure 3-figure supplement 1-source data 1/Figure 3 - figure supplement 1 Source data 1.pdf]

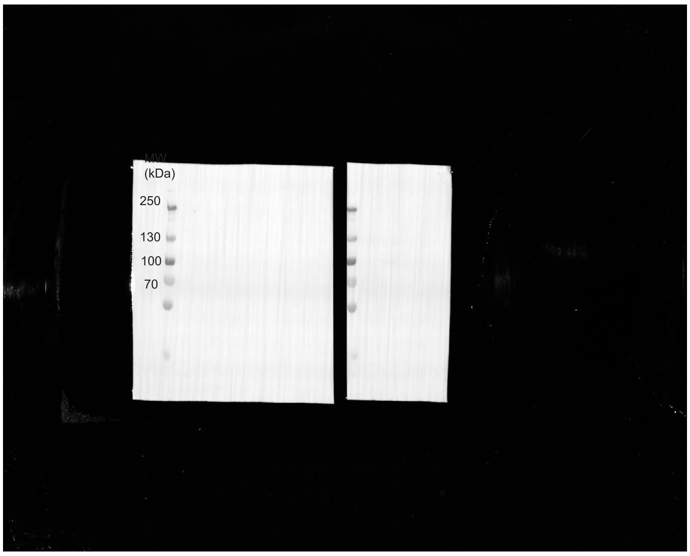

Blot image

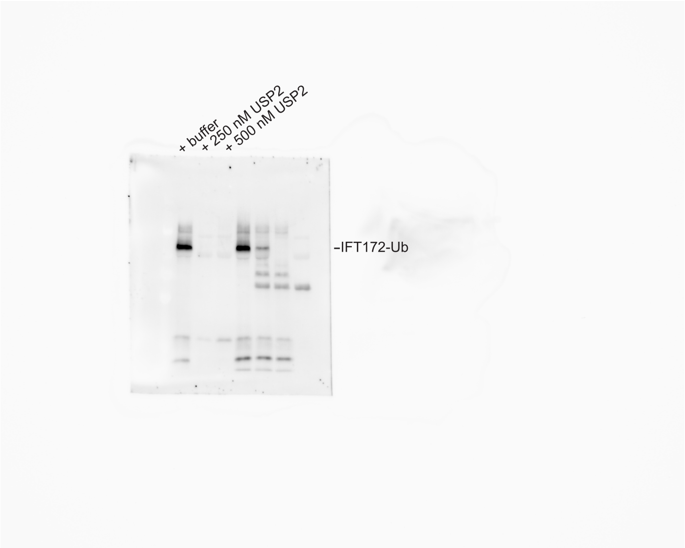

α-Ubiquitin

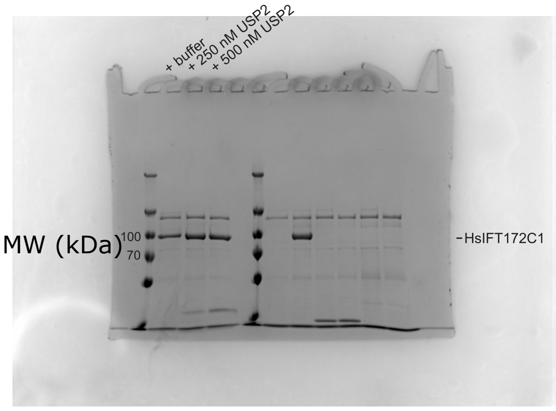

Coomassie

Original blot images, α-Ubiquitin blots and coomassie stainings used to generate Figure 3 - figure supplement 1, panel A, labelled according to the original figure panel.

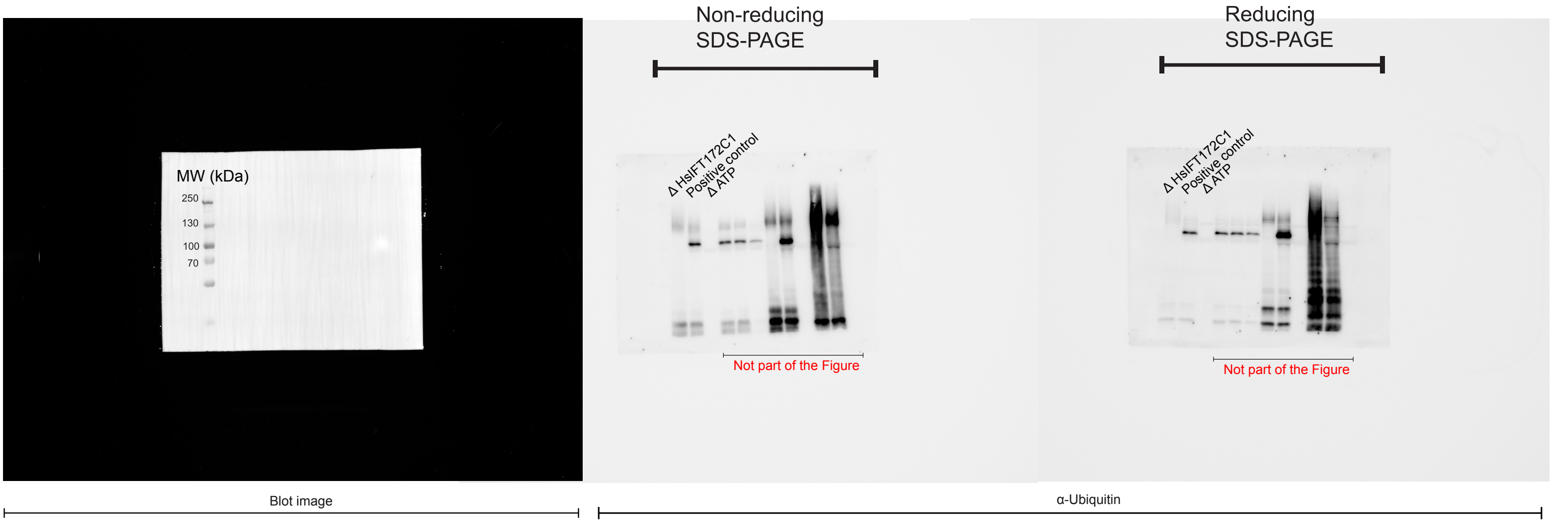

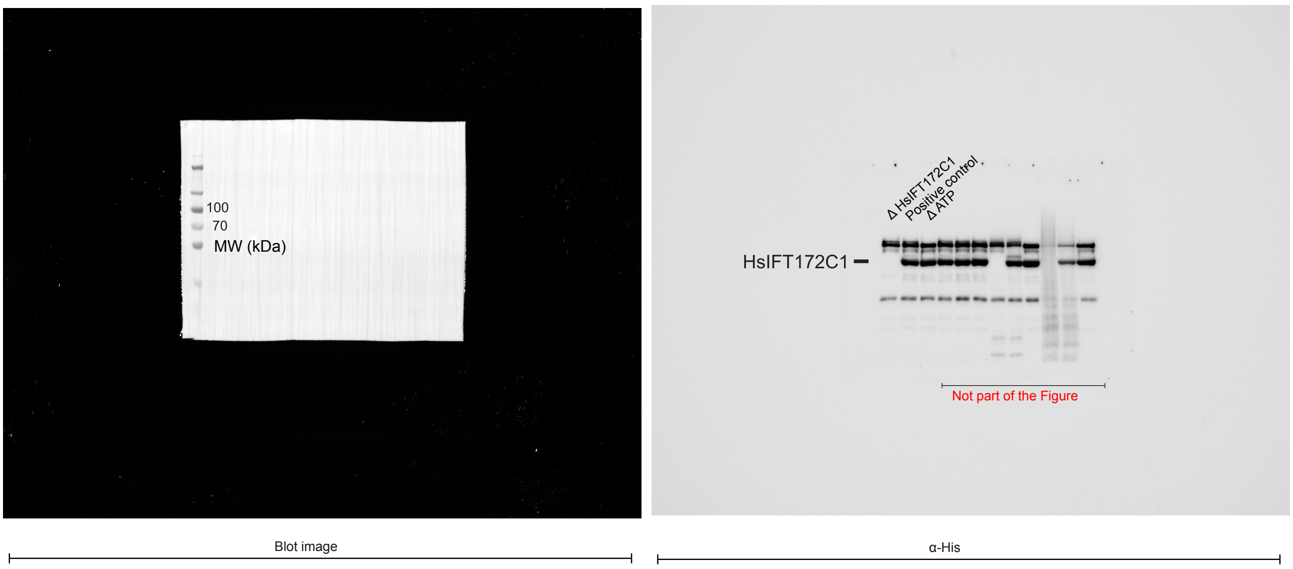

Original blot images, α-Ubiquitin blots and α-His blots used to generate Figure 3 - figure supplement 1, panel B, labelled according to the original figure panel.

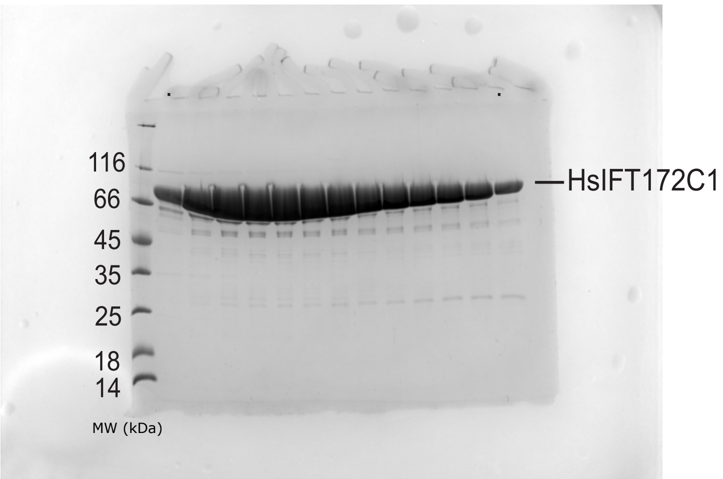

Coomassie Panel C

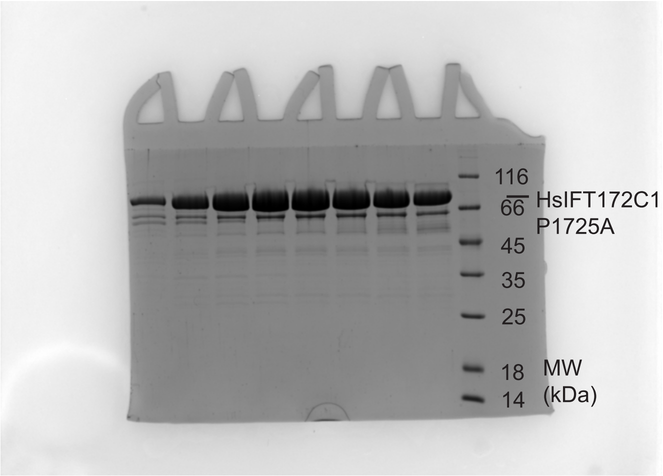

Coomassie Panel D

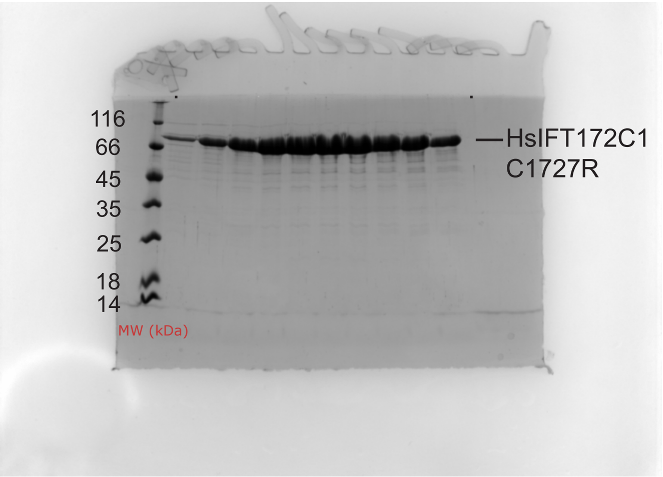

Coomassie Panel E

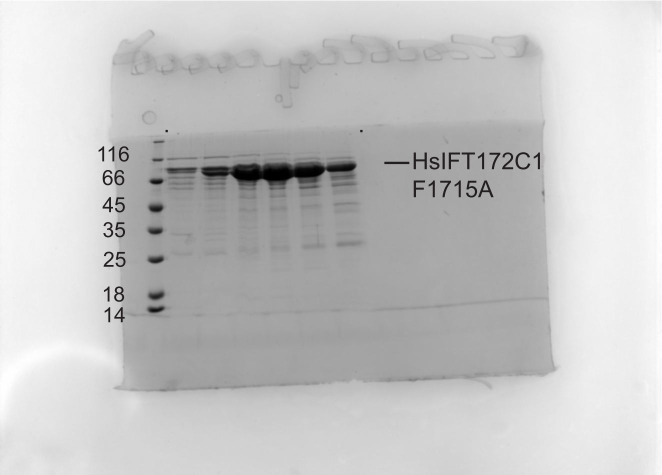

Coomassie Panel F

Original coomassie staining gels used to generate Figure 3 - figure supplement 1, panel C-F, labelled according to the original figure panels.
