# Supplementary material for: Intraflagellar transport protein IFT172 contains a C-terminal ubiquitin-binding U-box-like domain involved in ciliary signaling
Source: eLife. 2026 Jun 23;14:RP104906. doi: 10.7554/eLife.104906 (PMC13290226; doi:10.7554/eLife.104906)
Supplement: Figure 4—source data 1. [file elife-104906-fig4-data1.zip › Figure 4-source data 1/Figure 4 source data 1.pdf]

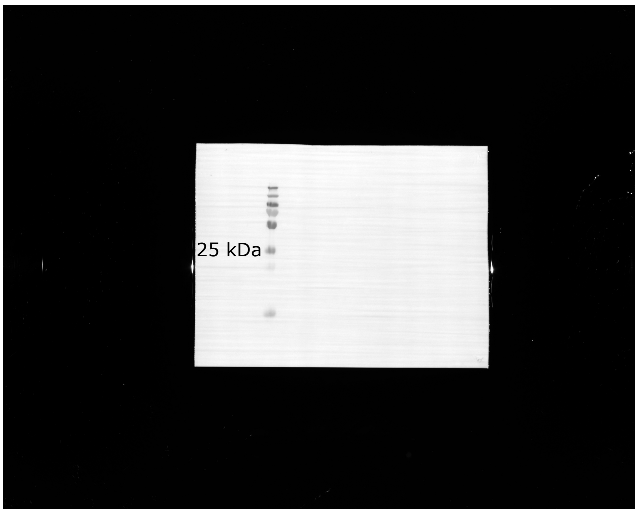

Blot image

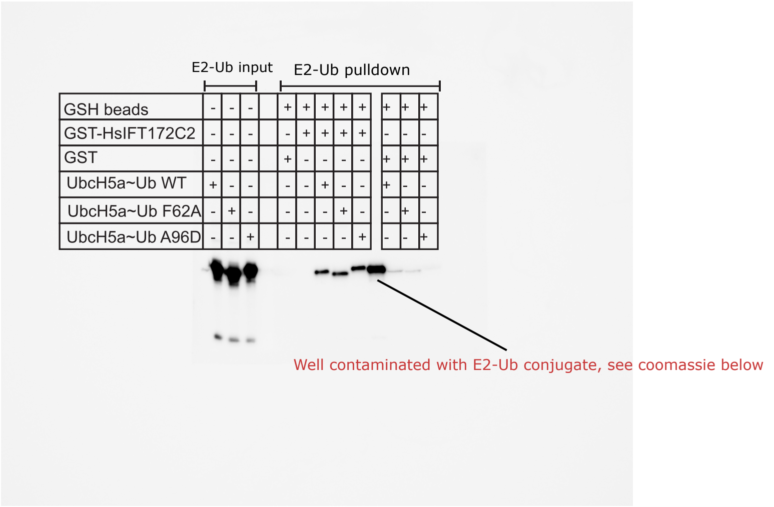

α-Ubiquitin

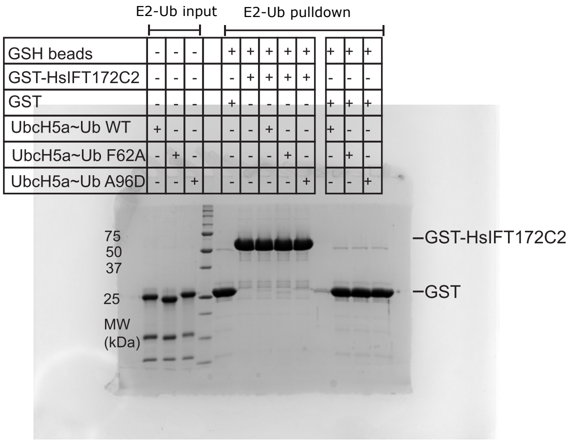

Coomassie

Original blot images, α-Ubiquitin blots and coomassie stainings used to generate Figure 4, panel A, labelled according to the original figure panel.

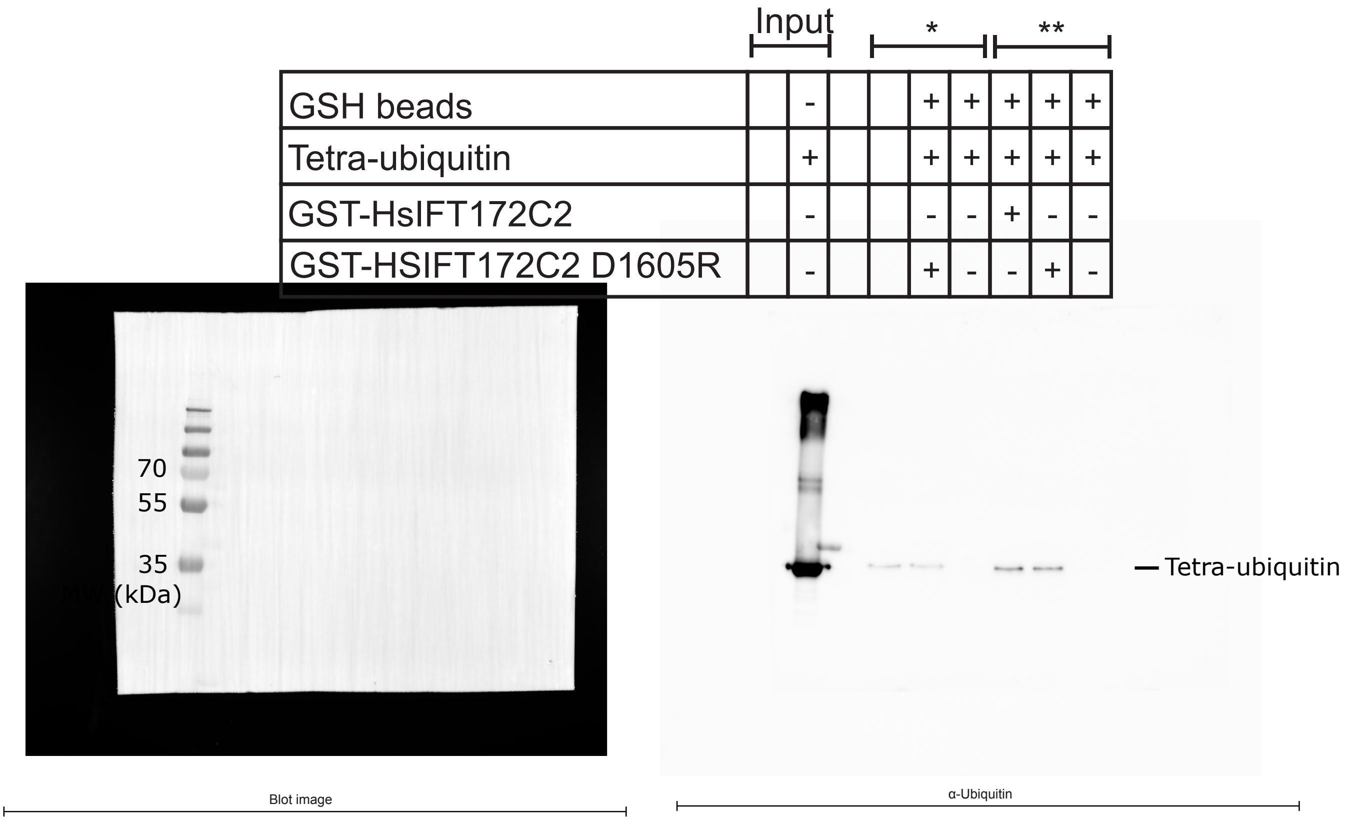

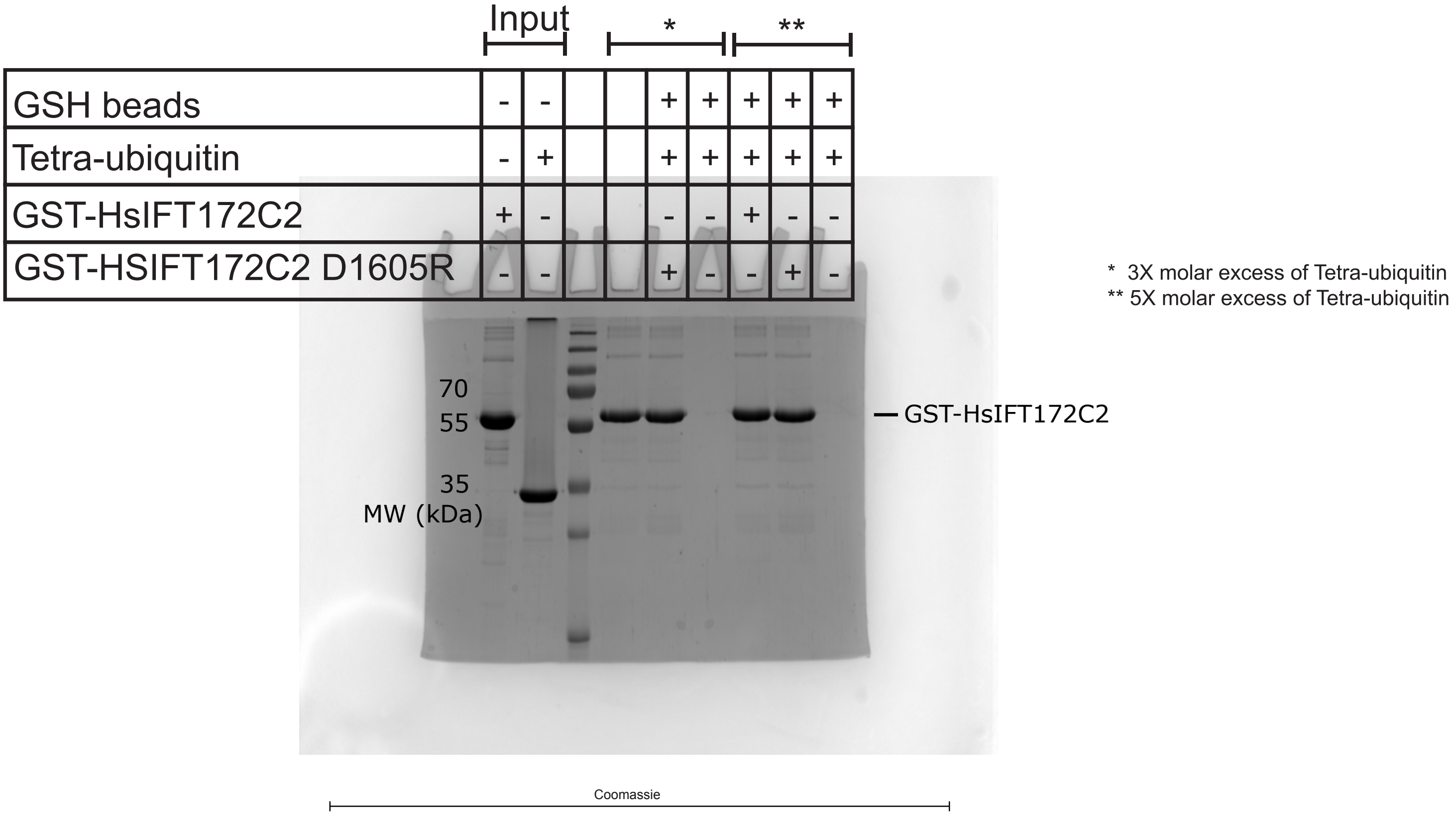

Original blot images, α-Ubiquitin blots and coomassie stainings used to generate Figure 4, panel C, labelled according to the original figure panel.

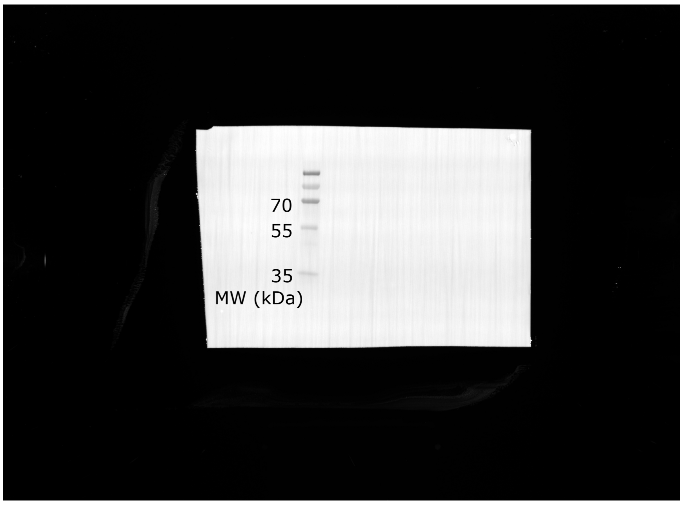

Blot image

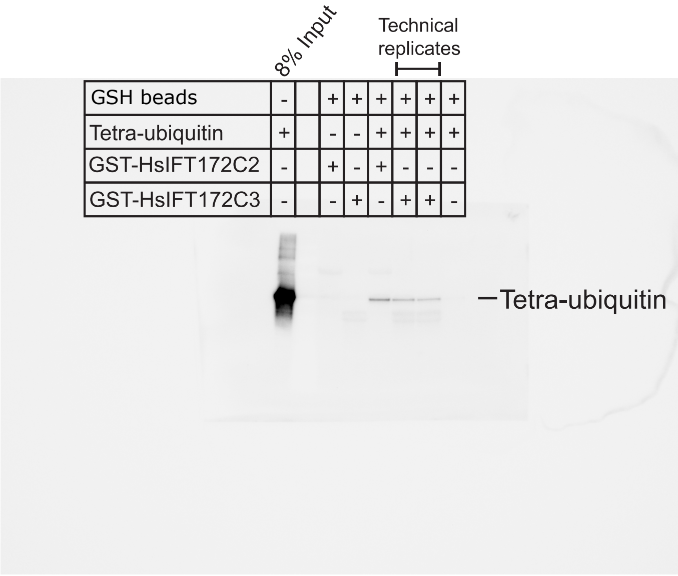

α-Ubiquitin

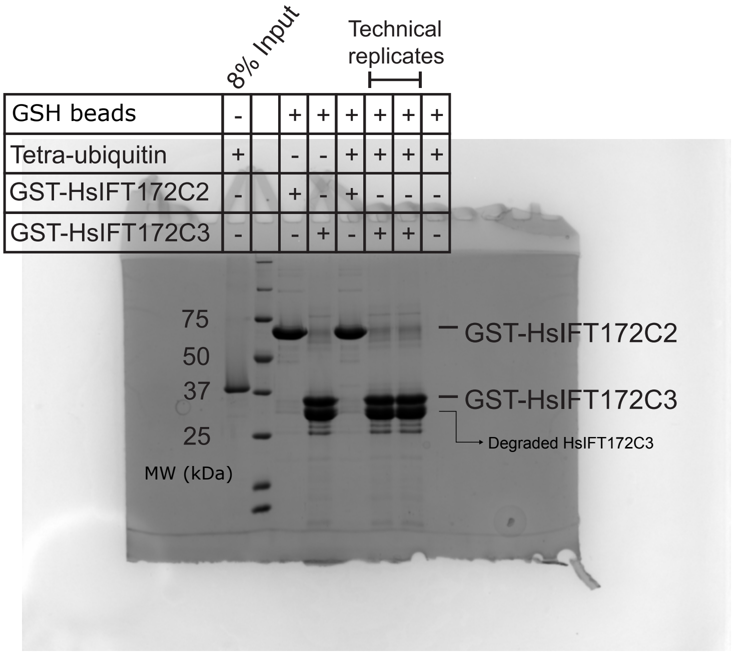

Coomassie

Original blot images, α-Ubiquitin blots and coomassie stainings used to generate Figure 4, panel D, labelled according to the original figure panel.
