# Supplementary material for: Intraflagellar transport protein IFT172 contains a C-terminal ubiquitin-binding U-box-like domain involved in ciliary signaling
Source: eLife. 2026 Jun 23;14:RP104906. doi: 10.7554/eLife.104906 (PMC13290226; doi:10.7554/eLife.104906)
Supplement: Figure 4—figure supplement 1—source data 1. [file elife-104906-fig4-figsupp1-data1.zip › Figure 4-figure supplement 1-source data 1/Figure 4- figure supplement 1 source data 1.pdf]

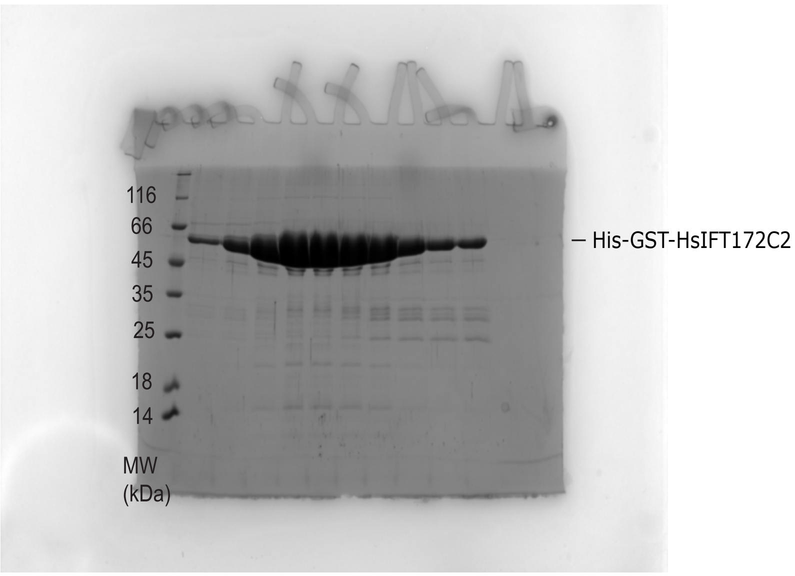

Coomassie Panel A

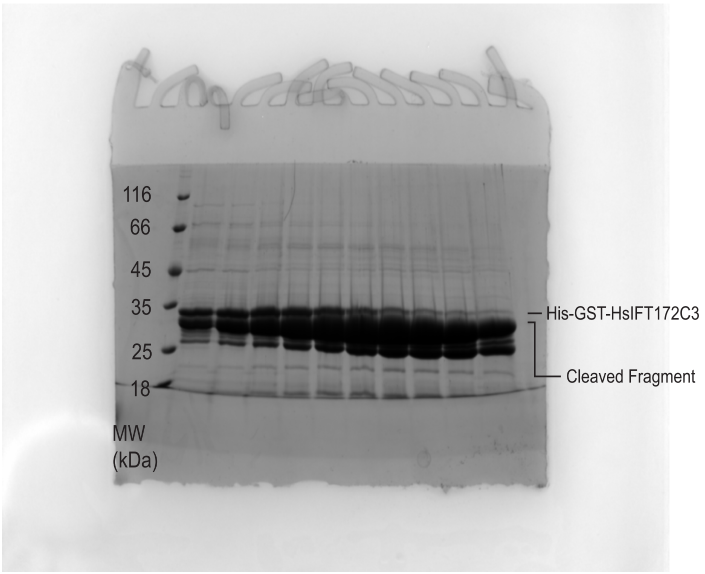

Coomassie Panel B

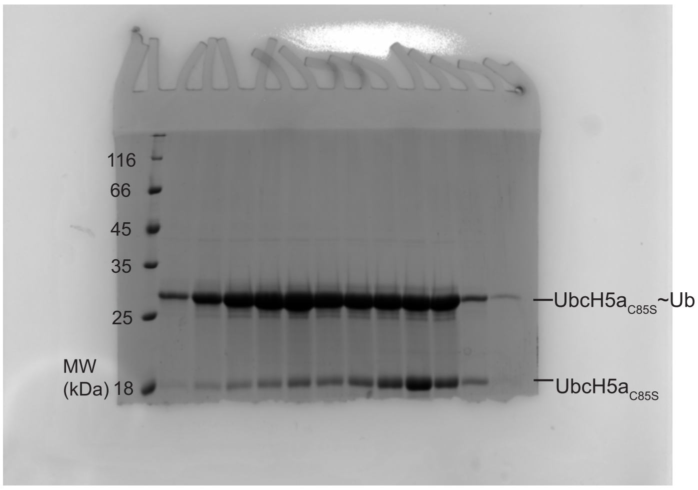

Coomassie Panel C

Original coomassie staining gels used to generate Figure 4 - figure supplement 1, panel A-C, labelled according to the original figure panel.
