# Supplementary material for: Intraflagellar transport protein IFT172 contains a C-terminal ubiquitin-binding U-box-like domain involved in ciliary signaling
Source: eLife. 2026 Jun 23;14:RP104906. doi: 10.7554/eLife.104906 (PMC13290226; doi:10.7554/eLife.104906)
Supplement: Figure 5—source data 1. [file elife-104906-fig5-data1.zip › Figure 5-source data 1/Figure 5 source data 1.pdf]

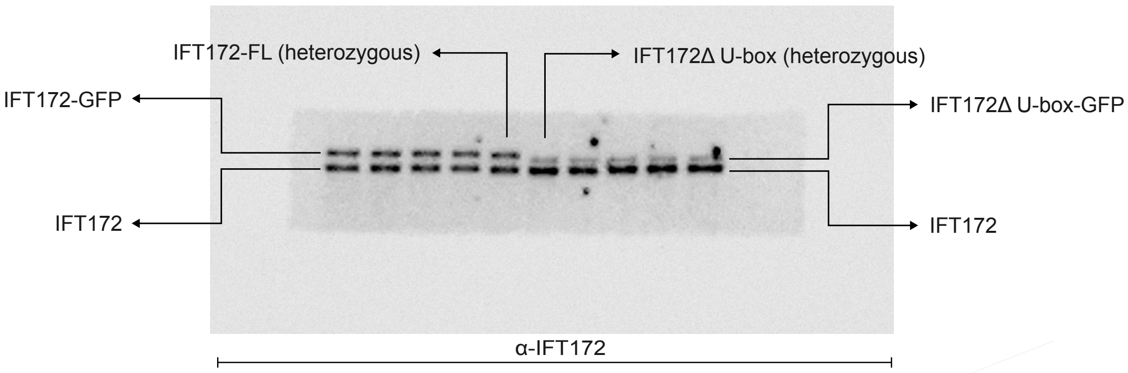


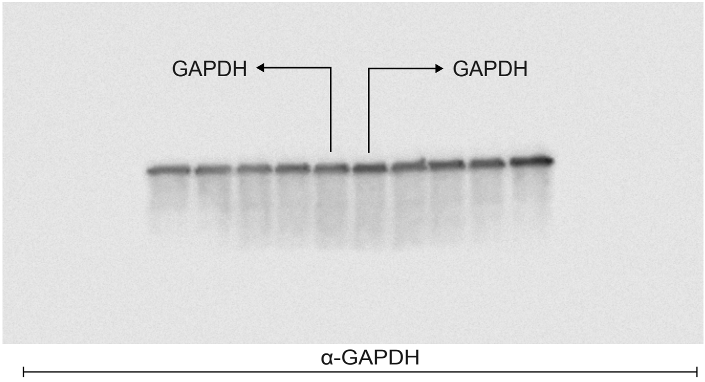


Original western blot aquisitions used to generate Figure 5, panel D, labelled according to the original figure panel. The marked wells and the remaining wells are part of a a TGF-β1 stimulation time course assay in the two respective cell lines.

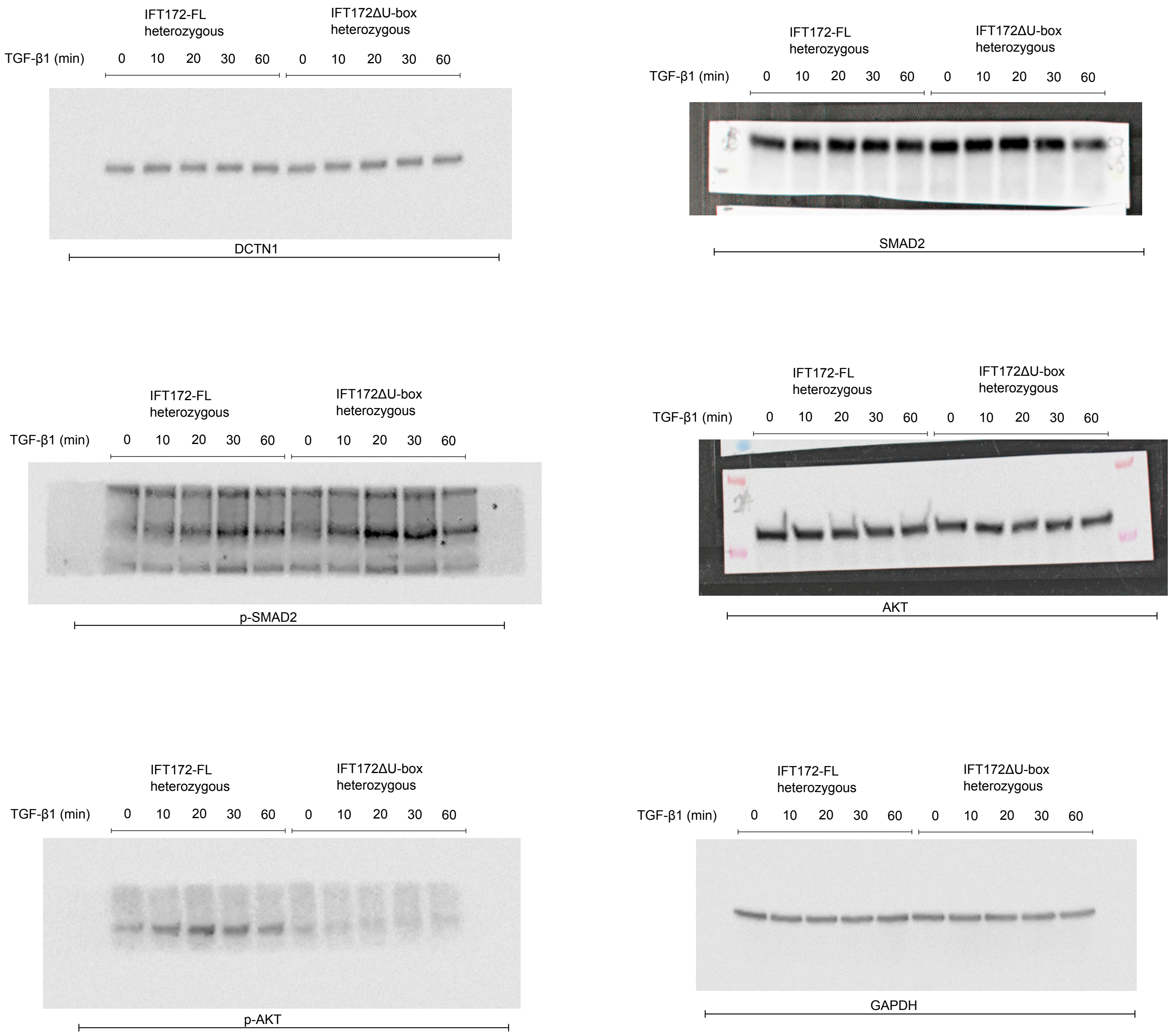


Original western blot aquisitions used to generate Figure 5, panel E, labelled according to the original figure panel.
