# Supplementary material for: Intraflagellar transport protein IFT172 contains a C-terminal ubiquitin-binding U-box-like domain involved in ciliary signaling
Source: eLife. 2026 Jun 23;14:RP104906. doi: 10.7554/eLife.104906 (PMC13290226; doi:10.7554/eLife.104906)
Supplement: Peer review history

# Editor report: eLife Assessment

**DOI:** 10.7554/eLife.104906.3.sa0

**Authors:** Junmin Pan

**Affiliations:** Tsinghua University China

**Keywords:** Convincing, Important

This **important** work advances our understanding of intraflagellar transport, ciliogenesis, and ciliary-based signaling, by identifying the interactions of IFT172 with IFT-A components, ubiquitin-binding, and ubiquitination, mediated by IFT172 C-terminus and its role in ciliogenesis and ciliary signaling. The evidence supporting the findings is **convincing**. This paper will be of interest to cell biologists and biochemists, especially those working on cilia and signaling.

# Referee report: Reviewer #1 (Public review)

**DOI:** 10.7554/eLife.104906.3.sa1

[Editors' note: this version has been assessed by the Reviewing Editor without further input from the original reviewers. The authors have addressed the comments raised in the previous round of review.]

Summary:

Zacharia and colleagues investigate the role of the C-terminus of IFT172 (IFT172c), a component of the IFT-B subcomplex. IFT172 is required for proper ciliary trafficking and mutations in its C-terminus are associated with skeletal ciliopathies. The authors begin by performing a pull-down to identify binding partners of His-tagged CrIFT172968-C in *Chlamydomonas reinhardtii* flagella. Interactions with three candidates (IFT140, IFT144, and a UBX-domain containing protein) are validated by AlphaFold Multimer with the IFT140 and IFT144 predictions in agreement with published cryo-ET structures of anterograde and retrograde IFT trains. They present a crystal structure of IFT172c and find that a part of the C-terminal domain of IFT172 resembles the fold of a non-canonical U-box domain. As U-box domains typically function to bind ubiquitin-loaded E2 enzymes, this discovery stimulates the authors to investigate the ubiquitin-binding and ubiquitination properties of IFT172c. Using in vitro ubiquitination assays with truncated IFT172c constructs, the authors demonstrate partial ubiquitination of IFT172c in the presence of the E2 enzyme UBCH5A. The authors also show a direct interaction of IFT172c with ubiquitin chains in vitro. Finally, the authors demonstrate that deletion of the U-box-like subdomain of IFT172 impairs ciliogenesis and TGFbeta signaling in RPE1 cells.

However, some of the conclusions of this paper are only partially supported by the data, and presented analyses are potentially governed by in vitro artifacts. In particular, the data supporting autoubiquitination and ubiquitin-binding are inconclusive. Without further evidence supporting a ubiquitin-binding role for the C-terminus, the title is potentially misleading.

Strengths:

(1) The pull-down with IFT172 C-terminus from *C. reinhardtii* cilia lysates is well performed and provides valuable insights into its potential roles.

(2) The crystal structure of the IFT172 C-terminus is of high quality.

(3) The presented AlphaFold-multimer predictions of IFT172c:IFT140 and IFT172c:IFT144 are convincing and agree with experimental cryo-ET data.

# Referee report: Reviewer #2 (Public review)

**DOI:** 10.7554/eLife.104906.3.sa2

Summary:

Cilia are antenna-like extensions projecting from the surface of most vertebrate cells. Protein transport along the ciliary axoneme is enabled by motor protein complexes with multimeric so-called IFT-A and IFT-B complexes attached. While the components of these IFT complexes have been known for a while, precise interactions between different complex members, especially how IFT-A and IFT-B subcomplexes interact, are still not entirely clear. Likewise, the precise underlying molecular mechanism in human ciliopathies resulting from IFT dysfunction has remained elusive.

Here, the authors investigated the structure and putative function of the to-date poorly characterised C-terminus of IFT-B complex member IFT172 using alpha-fold predictions, crystallography and biochemical analyses including proteomics analyses followed by mass spectrometry, pull-down assays, and TGFbeta signalling analyses using Chlamydomonas flagellae and RPE cells. The authors hereby provide novel insights into the crystal structure of IFT172 and identify novel interaction sites between IFT172 and the IFT-A complex members IFT140/IFT144. They suggest a U-box-like domain within the IFT172 C-terminus could play a role in IFT172 auto-ubiquitination as well as for TGFbeta signalling regulation.

As a number of disease-causing IFT72 sequence variants resulting in mammalian ciliopathy phenotypes in IFT172 have been previously identified in the IFT172 C-terminus, the authors also investigate the effects of such variants on auto-ubiquitination. This revealed no mutational effect on mono-ubiquitination which the authors suggest could be independent of the U-box-like domain but reduced overall IFT172 ubiquitination.

Strengths:

The manuscript is clear and well written and experimental data is of high quality. The findings provide novel insights into IFT172 function, IFT complex-A and B interactions, and they offer novel potential mechanisms that could contribute to the phenotypes associated with IFT172 C-terminal ciliopathy variants.

# Referee report: Reviewer #3 (Public review)

**DOI:** 10.7554/eLife.104906.3.sa3

Summary:

Zacharia et al report on the molecular function of the C-terminal domain of the intraflagellar transport IFT-B complex component IFT172 by structure determination and biochemical in vitro and cell culture-based assays. The authors identify an IFT-A binding site that mediates a mutually exclusive interaction to two different IFT-A subunits, IFT144 and IFT140, consistent with interactions suggested in anterograde and retrograde IFT trains by previous cryo-electron tomography studies. Additionally, the authors identify a U-box-like domain that binds ubiquitin and conveys ubiquitin conjugation activity in the presence of the UbcH5a E2 enzyme in vitro. RPE1 cell lines that lack the U-box domain show a reduction in ciliation rate with shorter cilia, and heterozygous cells manifest TGF-beta signaling defects, suggesting an involvement of the U-box domain in cilium-dependent signaling.

Strengths:

(1) The structural analyses of the C-terminal domain of IFT172 combine crystallography with structure prediction using state-of-the-art algorithms, which gives high confidence in the presented protein structures. The structure-based predictions of protein interactions are validated by further biochemical experiments to assess the specific binding of the IFT172 C-terminal domains with other proteins.

(2) The finding that the IFT172 C-terminus interactions with the IFT-A components IFT140 and IFT144 appear mutually exclusive confirm a suggested role in mediating the binding of IFT-B to IFT-A in anterograde and retrograde IFT trains, which is of very high scientific value.

(3) The suggested molecular mechanism of IFT train coordination explains previous findings in Chlamydomonas IFT172 mutants, in particular an IFT172 mutant that appeared defective in retrograde IFT, as well as mutations identified in ciliopathy patients.

(4) The identification of other IFT172 interactors by unbiased mass spectrometry-based proteomics is very exciting. Analysis of stoichiometries between IFT components suggests that these interactors could be part of IFT trains, either as cargos or additional components that may fulfill interesting functions in cilia and flagella.

(5) The authors unexpectedly identify a U-box-like fold in the IFT172 C-terminus and thoroughly dissect it by sequence and mutational analyses to reveal unexpected ubiquitin binding and potential intrinsic ubiquitination activity.

(6) The overall data quality is very high. The use of IFT172 proteins from different organisms suggests a conserved function.

Overall, the authors achieved to characterize an understudied protein domain of the ciliary intraflagellar transport machinery and gained important molecular insights into its role in primary cilia biology, beyond IFT. By identifying an unexpected functional protein domain and novel interaction partners the work makes an important contribution to further our understanding of how ciliary processes might be regulated by ubiquitination on a molecular level. Based on this work it will be important for future studies in the cilia community to consider direct ubiquitin binding by IFT complexes.

Conceptually, the study highlights that protein transport complexes can exhibit additional intrinsic structural features for potential auto-regulatory processes. Moreover, the study adds to the functional diversity of small U-box and ubiquitin-binding domains, which will be of interest to a broader cell biology and structural biology audience.

# Author comment: Author response

**DOI:** 10.7554/eLife.104906.3.sa4

**Authors:** Nevin K Zacharia, Stefanie Kuhns, Niels Boegholm, Anni Christensen, Jiaolong Wang, Narcis A Petriman, Anna Lorentzen, Jindriska L Fialova, Lucie Menguy, Sophie Saunier, Søren Tvorup Christensen, Jens S Andersen, Sagar Bhogaraju, Esben Lorentzen

**Affiliations:** Aarhus University Aarhus Denmark; Southern Danish University Odense Denmark; Department of Molecular Biology and Genetics, Aarhus University, Aarhus, Denmark Aarhus Denmark; Department of Molecular Biology and Genetics, Aarhus University, Aarhus, Denmark Aarhus Denmark; Department of Molecular Biology and Genetics, Aarhus University, Aarhus, Denmark Aarhus Denmark; Aarhus University Aarhus Denmark; Aarhus University Aarhus Denmark; University of Copenhagen Copenhagen Denmark; Institut Imagine Paris France; Institut Imagine Paris France; University of Copenhagen Copenhagen Denmark; University of Southern Denmark Odense Denmark; European Molecular Biology Laboratory Grenoble France; Aarhus University Aarhus Denmark

The following is the authors’ response to the original reviews.

**Public Reviews:**

**Reviewer #1 (Public review):**

Summary:

Zacharia and colleagues investigate the role of the C-terminus of IFT172 (IFT172c), a component of the IFT-B subcomplex. IFT172 is required for proper ciliary trafficking and mutations in its C-terminus are associated with skeletal ciliopathies. The authors begin by performing a pull-down to identify binding partners of His-tagged CrIFT172968-C in *Chlamydomonas reinhardtii* flagella. Interactions with three candidates (IFT140, IFT144, and a UBX-domain containing protein) are validated by AlphaFold Multimer with the IFT140 and IFT144 predictions in agreement with published cryo-ET structures of anterograde and retrograde IFT trains. They present a crystal structure of IFT172c and find that a part of the C-terminal domain of IFT172 resembles the fold of a non-canonical U-box domain. As U-box domains typically function to bind ubiquitin-loaded E2 enzymes, this discovery stimulates the authors to investigate the ubiquitin-binding and ubiquitination properties of IFT172c. Using in vitro ubiquitination assays with truncated IFT172c constructs, the authors demonstrate partial ubiquitination of IFT172c in the presence of the E2 enzyme UBCH5A. The authors also show a direct interaction of IFT172c with ubiquitin chains in vitro. Finally, the authors demonstrate that deletion of the U-box-like subdomain of IFT172 impairs ciliogenesis and TGFbeta signaling in RPE1 cells.

However, some of the conclusions of this paper are only partially supported by the data, and presented analyses are potentially governed by in vitro artifacts. In particular, the data supporting autoubiquitination and ubiquitin-binding are inconclusive. Without further evidence supporting a ubiquitin-binding role for the C-terminus, the title is potentially misleading.

Strengths:

(1) The pull-down with IFT172 C-terminus from *C. reinhardtii* cilia lysates is well performed and provides valuable insights into its potential roles.

(2) The crystal structure of the IFT172 C-terminus is of high quality.

(3) The presented AlphaFold-multimer predictions of IFT172c:IFT140 and IFT172c:IFT144 are convincing and agree with experimental cryo-ET data.

Weaknesses:

(1) The crystal structure of HsIFT172c reveals a single globular domain formed by the last three TPR repeats and C-terminal residues of IFT172. However, the authors subdivide this globular domain into TPR, linker, and U-box-like regions that they treat as separate entities throughout the manuscript. This is potentially misleading as the U-box surface that is proposed to bind ubiquitin or E2 is not surface accessible but instead interacts with the TPR motifs. They justify this approach by speculating that the presented IFT172c structure represents an autoinhibited state and that the U-box-like domain can become accessible following phosphorylation. However, additional evidence supporting the proposed autoinhibited state and the potential accessibility of the U-box surface following phosphorylation is needed, as it is not tested or supported by the current data.

We thank the reviewer for this comment. IFT172C contains TPR region and Ubox-like region, which are admittedly tightly bound to each other. While there is a possibility that this region functions and exists as one domain, below are the reasons why we chose to classify these regions as two different domains.

(1) TPR and Ubox-like regions are two different structural classes

(2) TPR region is linked to Ubox-like region via a long linker which seems poised to regulate the relative movement between these regions.

(3) Many ciliopathy mutations are mapped to the interface of TPR region and the Ubox region hinting at a regulatory mechanism governed by this interface.

That said, we agree that the proposed autoinhibited state and its potential relief by phosphorylation remains a hypothesis that requires experimental validation. We have revised the manuscript to present this more clearly as a speculative model rather than an established mechanism. We clearly acknowledge this limitation on pg. 16-17 of the revised discussion: ‘The IFT172 U-box domain appears to be in an auto-inhibited state in our crystal structure of HsIFT172C2 (Fig. 2E), potentially explaining the absence of a robust auto-ubiquitination activity in-vitro. This structural inhibition is reminiscent of the RING ubiquitin ligase CBL [59], where phosphorylation and substrate binding trigger a conformational change that activates ligase activity [59,75]. Intriguingly, the phosphosite database [76] lists four residues (T1533, S1549, T1689, Y1691) at the U-box/TPR interface as phosphorylation sites (Fig. S2D). Phosphorylation of these residues could potentially alleviate the auto-inhibited state, suggesting a possible regulatory mechanism. Furthermore, a 30-residue linker connects the U-box domain to the last TPR of IFT172, likely providing significant conformational flexibility (Fig. 2A-B). This flexibility may be functionally crucial for the U-box domain, allowing it to adopt different conformations as needed for its various roles. However, we note that the proposed autoinhibition model and its potential regulation by phosphorylation remain hypothetical and require future experimental validation.

(2) While in vitro ubiquitination of IFT172 has been demonstrated, in vivo evidence of this process is necessary to support its physiological relevance.

We thank the reviewer for this important point. We agree that in vivo evidence of IFT172 ubiquitination would strengthen the physiological relevance of our findings. While our current study focuses on the in vitro characterization of this activity, we have revised the manuscript to more clearly state that demonstration of IFT172 ubiquitination activity in cells, including identification of bona fide substrates, is required to establish its physiological significance (p. 16). We consider this an important direction for future studies.

(3) The authors describe IFT172 as being autoubiquitinated. However, the identified E2 enzymes UBCH5A and UBCH5B can both function in E3-independent ubiquitination (as pointed out by the authors) and mediate ubiquitin chain formation in an E3-independent manner in vitro (see ubiquitin chain ladder formation in Figure 3A). In addition, point mutation of known E3-binding sites in UBCH5A or TPR/U-box interface residues in IFT172 has no effect on the mono-ubiquitination of IFT172c1. Together, these data suggest that IFT172 is an E3-independent substrate of UBCH5A in vitro. The authors should state this possibility more clearly and avoid terminology such as "autoubiquitination" as it implies that IFT172 is an E3 ligase, which is misleading. Similarly, statements on page 10 and elsewhere are not supported by the data (e.g. "the low in vitro ubiquitination activity exhibited by IFT172" and "ubiquitin conjugation occurring on HsIFT172C1 in the presence of UBCH5A, possibly in coordination with the IFT172 U-box domain").

We now consider this possibility and tone down our statements about the autoubiquitination activity of IFT172 in both the abstract and results/discussion parts of the revised version of the manuscript. We no longer refer to IFT172 as having auto-ubiquitination activity in the manuscript.

(4) Related to the above point, the conclusion on page 11, that mono-ubiquitination of IFT172 is U-box-independent while polyubiquitination of IFT172 is U-box-dependent appears implausible. The authors should consider that UBCH5A is known to form free ubiquitin chains in vitro and structural rearrangements in F1715A/C1725R variants could render additional ubiquitination sites or the monoubiquitinated form of IFT172 inaccessible/unfavorable for further processing by UBCH5A.

We agree and the conclusion on pg. 11 has now been changed to: Therefore, while mutations in the IFT172 U-box domain affect the formation of higher molecular weight ubiquitin conjugates, the prominent mono-ubiquitination of IFT172 is likely attributable to the E3-independent activity of UbcH5a, as this event is not impacted by these U-box mutations, rather than indicating an intrinsic auto-ubiquitination capacity of IFT172 itself.

(5) Identification of the specific ubiquitination site(s) within IFT172 would be valuable as it would allow targeted mutation to determine whether the ubiquitination of IFT172 is physiologically relevant. Ubiquitination of the C1 but not the C2 or C3 constructs suggests that the ubiquitination site is located in TPRs ranging from residues 969-1470. Could this region of TPR repeats (lacking the IFT172C3 part) suffice as a substrate for UBCH5A in ubiquitination assays?

We thank the reviewer for raising this important point about ubiquitination site identification. While not included in our manuscript, we did perform mass spectrometry analysis of ubiquitination sites using wild-type IFT172 and several mutants (P1725A, C1727R, and F1715A). As shown in Author response image 1, we detected multiple ubiquitination sites across these constructs. The wild-type protein showed ubiquitination at positions K1022, K1237, K1271, and K1551, while the mutants displayed slightly different patterns of modification. However, we should note that the MS intensity signals for these ubiquitinated peptides were relatively low compared to unmodified peptides, making it difficult to draw strong conclusions about site specificity or physiological relevance.

**Author response image 1.** 

Consistent with the reviewer's suggestion, all detected ubiquitination sites fall within the TPR-containing region (residues 1022-1551), which is present in the C1 construct but absent from C2 and C3, explaining the construct-dependent ubiquitination pattern. We did not test the TPR region alone as a UBCH5A substrate, but this would be an informative experiment for future studies.

(6) The discrepancy between the molecular weight shifts observed in anti-ubiquitin Western blots and Coomassie-stained gels is noteworthy. The authors show the appearance of a mono-ubiquitinated protein of ~108 kDa in anti-ubiquitin Western blots. However, this molecular weight shift is not observed for total IFT172 in the corresponding Coomassie-stained gels (Figures 3B, D, F). Surprisingly, this MW shift is visible in an anti-His Western blot of a ubiquitination assay (Fig 3C). Together, this raises the concern that only a small fraction of IFT172 is being modified with ubiquitin. Quantification of the percentage of ubiquitinated IFT172 in the in vitro experiments could provide helpful context.

We acknowledge that the ubiquitin conjugation of IFT172 in vitro is weak, as stated in the manuscript (p. 16). The discrepancy between anti-ubiquitin Western blots and Coomassie-stained gels is consistent with only a small fraction of IFT172 being modified, which is expected given that the reaction likely reflects E3-independent ubiquitination by UBCH5A rather than a robust enzymatic activity of IFT172 itself. The anti-His Western blot (Fig. 3C) is more sensitive than Coomassie staining, explaining why the shift is visible there but not on Coomassie. We have not performed formal quantification of the ubiquitinated fraction, but based on the Coomassie data, we estimate it to be a minor proportion of total IFT172, consistent with the toned-down conclusions in our revised manuscript. The identification of physiological substrates and in vivo validation will be important future directions to establish the biological relevance of these observations.

(7) The authors propose that IFT172 binds ubiquitin and demonstrate that GST-tagged HsIFT172C2 or HsIFT172C3 can pull down tetra-ubiquitin chains. However, ubiquitin is known to be "sticky" and to have a tendency for weak, nonspecific interactions with exposed hydrophobic surfaces. Given that only a small proportion of the ubiquitin chains bind in the pull-down, specific point mutations that identify the ubiquitin-binding site are required to convincingly show the ubiquitin binding of IFT172.

We appreciate the reviewer's point regarding the potential for non-specific ubiquitin interactions and the value of mutational analysis for confirming specificity. While further mutagenesis of the predicted ubiquitin-binding interface was not performed for this revision, we note that our data show comparable tetra-ubiquitin pull-down by both the larger HsIFT172C2 construct and, importantly, the isolated HsIFT172C3 U-box domain itself (Fig. 4D). This localization of binding to the smaller U-box domain, coupled with our AlphaFold model predicting a specific interface with ubiquitin (Fig. 4E-F) and the observation that a mutation elsewhere (D1605R, Fig. 4C) does not abrogate this binding, collectively suggest a degree of specificity. We have revised the manuscript to more cautiously present these findings and acknowledge the need for future studies to definitively map the binding site. Specifically, we have now toned down the conclusion in the section on pg. 12-13 of the revised manuscript including a toned down heading: “IFT172 U-box domain pulls down ubiquitin in vitro”.

(8) The authors generated structure-guided mutations based on the predicted Ub-interface and on the TPR/U-box interface and used these for the ubiquitination assays in Fig 3. These same mutations could provide valuable insights into ubiquitin binding assays as they may disrupt or enhance ubiquitin binding (by relieving "autoinhibition"), respectively. Surprisingly, two of these sites are highlighted in the predicted ubiquitin-binding interface (F1715, I1688; Figure 4E) but not analyzed in the accompanying ubiquitin-binding assays in Figure 4.

We thank the reviewer for emphasizing the importance of mutational analysis to confirm the specificity of ubiquitin binding and for specifically inquiring about residues like F1715 and I1688 at the predicted ubiquitin interface. We tested purified HsIFT172C1 constructs containing the F1715A mutation (along with P1725A and C1727R variants) in pull-down assays with GST-Ubiquitin, see Author response image 2.

**Author response image 2.** 

However, these experiments did not reveal a conclusive difference in ubiquitin binding for any of the tested variants compared to wild-type IFT172. The I1688A mutant, unfortunately, yielded insoluble protein and could not be evaluated. It is conceivable that the F1715A mutation was not disruptive enough to significantly alter binding, and future studies with different substitutions might be more informative. Nevertheless, our observations that the isolated HsIFT172C3 U-box domain itself pulls down tetra-ubiquitin (Fig. 4D), that our AlphaFold model predicts a specific interface (Fig. 4E-F), and that a mutation elsewhere (D1605R, Fig. 4C) does not abrogate this binding, collectively suggest a degree of specificity. We have revised the manuscript to present these ubiquitin binding findings cautiously, acknowledging the need for further investigation to definitively map the binding site and its functional relevance.

(9) If IFT172 is a ubiquitin-binding protein, it might be expected that the pull-down experiments in Figure S1 would identify ubiquitin, ubiquitinated proteins, or E2 enzymes. These were not observed, raising doubt that IFT172 is a ubiquitin-binding protein.

We acknowledge that the absence of ubiquitin or ubiquitinated proteins in our pull-down/MS experiment (Fig. S1) could raise questions about the ubiquitin-binding capacity of IFT172. However, several technical factors likely explain this. First, IFT172 appears to bind ubiquitin with low affinity, as indicated by our in vitro pull-downs and the AF-predicted interface. Second, we used extensive washes to remove non-specific interactors, which would also remove weak but potentially genuine ubiquitin interactions. Third, we did not include ubiquitination-preserving reagents such as NEM in our pull-down buffers, exposing ubiquitinated proteins to DUB-mediated deubiquitination during the experiment. These factors combined would strongly select against the detection of ubiquitin-related interactors under our experimental conditions.

(10) The cell-based experiments demonstrate that the U-box-like region is important for the stability of IFT172 but does not demonstrate that the effect on the TGFb pathway is due to the loss of ubiquitin-binding or ubiquitination activity of IFT172.

We acknowledge that our current data cannot definitively distinguish whether the TGFβ pathway defects arise from reduced IFT172 protein stability or from specific loss of ubiquitin-related functions of the U-box domain. Our experiments demonstrate that the U-box region is required for both IFT172 stability and proper TGFβ signaling, but we agree that establishing a direct mechanistic link between ubiquitin-binding/conjugation and signaling would require additional experiments such as point mutations that selectively disrupt ubiquitin-related activity without affecting protein stability. We have revised the discussion (p. 18-19) to more clearly acknowledge this limitation. Addition to text: “However, we note that our current experiments cannot distinguish whether these signaling effects result specifically from loss of ubiquitin-related functions of the U-box domain or from the reduced levels of functional IFT172 protein in the heterozygous U-box deleted cells. Targeted point mutations that selectively disrupt ubiquitin binding without affecting protein stability would be required to resolve this question.”

(11) The challenges in experimentally validating the interaction between IFT172 and the UBX-domain-containing protein are understandable. Alternative approaches, such as using single domains from the UBX protein, implementing solubilizing tags, or disrupting the predicted binding interface in Chlamydomonas flagella pull-downs, could be considered. In this context, the conclusion on page 7 that "The uncharacterized UBX-domain-containing protein was validated by AF-M as a direct IFT172 interactor" is incorrect as a prediction of an interaction interface with AF-M does not validate a direct interaction per se.

We agree with the reviewer that our AlphaFold-Multimer (AF-M) predictions alone do not constitute experimental validation of a direct interaction. We appreciate the reviewer's understanding of the technical challenges in validating this interaction experimentally. We have revised our text (p. 7) to state that "The uncharacterized UBX-domain-containing protein was predicted by AF-M as a potential direct IFT172 interactor" and discuss the AF-M predictions as computational evidence that suggests, but does not prove, a direct interaction.

**Reviewer #2 (Public review):**

Summary:

Cilia are antenna-like extensions projecting from the surface of most vertebrate cells. Protein transport along the ciliary axoneme is enabled by motor protein complexes with multimeric so-called IFT-A and IFT-B complexes attached. While the components of these IFT complexes have been known for a while, precise interactions between different complex members, especially how IFT-A and IFT-B subcomplexes interact, are still not entirely clear. Likewise, the precise underlying molecular mechanism in human ciliopathies resulting from IFT dysfunction has remained elusive.

Here, the authors investigated the structure and putative function of the to-date poorly characterised C-terminus of IFT-B complex member IFT172 using alpha-fold predictions, crystallography and biochemical analyses including proteomics analyses followed by mass spectrometry, pull-down assays, and TGFbeta signalling analyses using chlamydomonas flagellae and RPE cells. The authors hereby provide novel insights into the crystal structure of IFT172 and identify novel interaction sites between IFT172 and the IFT-A complex members IFT140/IFT144. They suggest a U-box-like domain within the IFT172 C-terminus could play a role in IFT172 auto-ubiquitination as well as for TGFbeta signalling regulation.

As a number of disease-causing IFT72 sequence variants resulting in mammalian ciliopathy phenotypes in IFT172 have been previously identified in the IFT172 C-terminus, the authors also investigate the effects of such variants on auto-ubiquitination. This revealed no mutational effect on mono-ubiquitination which the authors suggest could be independent of the U-box-like domain but reduced overall IFT172 ubiquitination.

Strengths:

The manuscript is clear and well written and experimental data is of high quality. The findings provide novel insights into IFT172 function, IFT complex-A and B interactions, and they offer novel potential mechanisms that could contribute to the phenotypes associated with IFT172 C-terminal ciliopathy variants.

Weaknesses:

Some suggestions/questions are included in the comments to the authors below.

**Reviewer #3 (Public review):**

Summary:

Zacharia et al report on the molecular function of the C-terminal domain of the intraflagellar transport IFT-B complex component IFT172 by structure determination and biochemical in vitro and cell culture-based assays. The authors identify an IFT-A binding site that mediates a mutually exclusive interaction to two different IFT-A subunits, IFT144 and IFT140, consistent with interactions suggested in anterograde and retrograde IFT trains by previous cryo-electron tomography studies. Additionally, the authors identify a U-box-like domain that binds ubiquitin and conveys ubiquitin conjugation activity in the presence of the UbcH5a E2 enzyme in vitro. RPE1 cell lines that lack the U-box domain show a reduction in ciliation rate with shorter cilia, and heterozygous cells manifest TGF-beta signaling defects, suggesting an involvement of the U-box domain in cilium-dependent signaling.

Strengths:

(1) The structural analyses of the C-terminal domain of IFT172 combine crystallography with structure prediction using state-of-the-art algorithms, which gives high confidence in the presented protein structures. The structure-based predictions of protein interactions are validated by further biochemical experiments to assess the specific binding of the IFT172 C-terminal domains with other proteins.

(2) The finding that the IFT172 C-terminus interactions with the IFT-A components IFT140 and IFT144 appear mutually exclusive confirm a suggested role in mediating the binding of IFT-B to IFT-A in anterograde and retrograde IFT trains, which is of very high scientific value.

(3) The suggested molecular mechanism of IFT train coordination explains previous findings in Chlamydomonas IFT172 mutants, in particular an IFT172 mutant that appeared defective in retrograde IFT, as well as mutations identified in ciliopathy patients.

(4) The identification of other IFT172 interactors by unbiased mass spectrometry-based proteomics is very exciting. Analysis of stoichiometries between IFT components suggests that these interactors could be part of IFT trains, either as cargos or additional components that may fulfill interesting functions in cilia and flagella.

(5) The authors unexpectedly identify a U-box-like fold in the IFT172 C-terminus and thoroughly dissect it by sequence and mutational analyses to reveal unexpected ubiquitin binding and potential intrinsic ubiquitination activity.

(6) The overall data quality is very high. The use of IFT172 proteins from different organisms suggests a conserved function.

Weaknesses:

(1) Interaction studies were carried out by pulldown experiments, which identified more IFT172 interaction partners. Whether these interactions can be seen in living cells remains to be elucidated in subsequent studies.

We agree with the reviewer that validation of protein-protein interactions in living cells provides important physiological context. While our pulldown experiments have identified several promising interaction partners and the AF-M predictions provide computational support for these interactions, we acknowledge that demonstrating these interactions in vivo would strengthen our findings. However, we believe our current biochemical and structural analyses provide valuable insights into the molecular basis of IFT172's interactions, laying important groundwork for future cell-based studies.

(2) The cell culture-based experiments in the IFT172 mutants are exciting and show that the U-box domain is important for protein stability and point towards involvement of the U-box domain in cellular signaling processes. However, the characterization of the generated cell lines falls behind the very rigorous analysis of other aspects of this work.

We thank the reviewer for noting that the characterization of our cell lines could be more rigorous. In the revised version of the manuscript, we have addressed this by providing additional validation data for all four engineered RPE1 cell lines. First, we performed Sanger sequencing to confirm precise in-frame integration of the GFP tag at the targeted loci and to exclude unintended insertions or deletions (indels), both for the full-length IFT172-eGFP lines (Fig. S6) and for the IFT172∆U-box-eGFP lines (Fig. S7). Second, we performed anti-IFT172 immunoblotting on all four cell lines alongside parental RPE1 cells, confirming expression of both the full-length and U-box-truncated IFT172 proteins (Fig. S8). Notably, the immunoblot revealed reduced steady-state levels of the IFT172∆U-box protein compared to full-length IFT172, providing direct biochemical evidence that loss of the U-box domain compromises IFT172 protein stability consistent with the ciliogenesis phenotype described in the main text. Together, these data verify the integrity of the edited loci at both the genomic and protein levels, and strengthen the validation of the cellular models used in this study.

Overall, the authors achieved to characterize an understudied protein domain of the ciliary intraflagellar transport machinery and gained important molecular insights into its role in primary cilia biology, beyond IFT. By identifying an unexpected functional protein domain and novel interaction partners the work makes an important contribution to further our understanding of how ciliary processes might be regulated by ubiquitination on a molecular level. Based on this work it will be important for future studies in the cilia community to consider direct ubiquitin binding by IFT complexes.

Conceptually, the study highlights that protein transport complexes can exhibit additional intrinsic structural features for potential auto-regulatory processes. Moreover, the study adds to the functional diversity of small U-box and ubiquitin-binding domains, which will be of interest to a broader cell biology and structural biology audience.

Additional comments:

The authors investigate the consequences of the U-box deletion on ciliary TGF-beta signaling. While a cilium-dependent effect of TGF-beta signaling on the phosphorylation of SMAD2 has been demonstrated, the precise function of cilia in AKT signaling has not been fully established in the field. Therefore, the relevance of this finding is somewhat unclear. It may help to discuss relevant literature on the topic, such as Shim et al., PNAS, 2020.

We appreciate the reviewer's comment highlighting that the role of primary cilia in AKT signaling is not as well established as for SMAD2/3. However, we note that a direct functional link between AKT signaling and ciliogenesis has been demonstrated, showing that AKT regulates ciliogenesis initiation through a Rab11-effector switch mechanism (Walia et al., 2019; PMID: 31204173, co-authored by the corresponding author of this study). Furthermore, Shim et al. (PMID: 33753495) demonstrated a cilia-dependent reciprocal activation of AKT1 and SMAD2/3. In the revised manuscript (p. 19, ref. 97), we have expanded the discussion to cite these studies and provide a clearer literature context for the cilia-AKT connection, while acknowledging that the precise mechanism by which the IFT172 U-box domain influences AKT activation requires further investigation.

**Recommendations for the authors:**

**Reviewer #1 (Recommendations for the authors):**

Points for the discussion:

(1) The discussion should mention that IFT-A subunits IFT121, IFT122 and IFT144 share a similar domain organization to IFT172 (TPRs terminating in Zn-finger-like domains). Do the authors consider these as potential ubiquitin-binding proteins with E3 ligase activity? The possibility that these Zn-finger-like regions share a common origin, and function to stabilize the proteins or mediate IFT subunit interactions without a role in ubiquitin biology should be considered.

We appreciate this important point. We agree that the shared domain architecture across IFT121, IFT122, IFT144, and IFT172 raises the question of whether these C-terminal domains primarily serve structural rather than ubiquitin-related roles. We have added a discussion paragraph (p. 16) acknowledging that a structural/stabilizing function is the more parsimonious explanation, while noting that whether IFT172's U-box-like domain has additionally acquired ubiquitin-related activity remains an open question.

(2) From their modeling data, do the authors have an explanation for why a substitution as conservative as D1605E would cause disease?

The D1605E substitution maps to the IFT172-IFT-A interaction interface (Fig. 1F). While this is a conservative change, D1605 is located at a tightly packed protein-protein interface where even the addition of a single methylene group (the difference between aspartate and glutamate) could introduce steric clashes with residues of IFT140 or IFT144, or alter the precise geometry of hydrogen bonds or salt bridges critical for the interaction. Unfortunately, this level of detail is beyond the resolution of AlphaFold models. However, the fact that this residue is positioned directly at the binding interface provides a plausible structural rationale for its pathogenicity.

(3) The authors speculate that the L1615P mutation in the Chlamydomonas fla11 strain causes a faulty switch to retrograde IFT and this provides a molecular basis for the retrograde IFT phenotype. However, because the mutation is also within the IFT144 binding site, why is anterograde IFT also not affected?

The fla11 L1615P mutation resides in helix αA, which participates in both IFT144 (anterograde) and IFT140 (retrograde) interactions. The predominantly retrograde phenotype can be rationalized by the fundamentally different structural roles of the IFT172 C-terminus in anterograde versus retrograde trains. In anterograde trains, the IFT172 C-terminus acts as a flexible tether in stoichiometric excess (2:1 IFT-B:IFT-A ratio), providing an avidity effect that likely compensates for reduced binding affinity caused by L1615P (Lacey et al., 2023). Additional lateral interactions between IFT-B subunits further stabilize the anterograde polymer independently of the IFT172-IFT144 link. In contrast, the retrograde train requires the IFT172 C-terminus to adopt a rigid, resolved conformation that is integral to the IFT-A dimeric interface, with no redundant lateral interactions to compensate (Lacey et al., 2024). The helix-breaking L1615P mutation would specifically disrupt this precise structural requirement, explaining the selective retrograde IFT defect in fla11. We have added this discussion to the revised manuscript (p. 16).

Minor:

(1) On page 5, the authors describe the fla11 phenotypes including accumulation of IFT particles at the tip and accumulation of ubiquitinated proteins in the cilium. Could the authors please expand on how this suggests that IFT172 could be involved in ciliary ubiquitination events and discuss an alternative scenario of impaired assembly of functional retrograde IFT in this strain leading to accumulation of ubiquitinated proteins?

In the revised manuscript (p. 16), we have expanded the discussion of the fla11 phenotype to address this point. We now discuss how the distinct structural roles of the IFT172 C-terminus in anterograde versus retrograde trains explain the selective retrograde IFT defect in fla11, and explicitly note that the accumulation of ubiquitinated proteins in fla11 cilia may reflect impaired retrograde IFT-mediated clearance rather than a direct role of IFT172 in ciliary ubiquitination.

(2) The authors should also expand on the literature of known UBX-IFT interactions in their manuscript (e.g. Raman et al. PMID 26389662).

We have expanded the discussion of UBX-IFT interactions in the revised manuscript (p. 7) by citing the work of Raman et al. (PMID 26389662), who identified a direct interaction between the UBX-domain protein UBXN10 and IFT-B via CLUAP1/IFT38 for VCP-mediated regulation of IFT complex integrity. This provides important context for our identification of a UBX-domain protein as an IFT172 interactor.

(3) On page 11, I1688 is incorrectly referred to as I688.

Fixed.

**Reviewer #2 (Recommendations for the authors):**

(1) The finding that the interaction with IFT140/144 is mutually exclusive is very interesting. Could you speculate on or do you have any data regarding the effects to the overall IFT-complex conformation and downstream biological effects depending on which partner is bound?

I am not a structural biologist so this may be an irrelevant/impossible-to-answer question: I was also wondering as Ref 46 has shown that the dynein-2 motor complex binds to the edge of IFT-B2 (for assembled trains): Could the IFT172 C-terminus be involved here or somehow influence this interaction? In your mass spec data from Cr cilia using CrIFT172_968-C you don`t mention pulling down dynein-2 components so there doesn`t seem to be a direct interaction, but could the IFT-B2 conformation depend on if IFT172 has bound IFT-140 or IFT144 and hence this interaction influence the dynein-2 binding?

We thank the reviewer for this insightful question. Based on recent cryo-ET structures of anterograde and retrograde IFT trains (Lacey et al., 2023; 2024), the switch from IFT144 to IFT140 binding fundamentally changes IFT172's structural role. In anterograde trains, the IFT172 C-terminus acts as a flexible tether tolerating the 2:1 IFT-B:IFT-A stoichiometry and permitting long polymer formation. In retrograde trains, it adopts a rigid conformation integral to the IFT-A dimeric interface, driving the formation of discrete retrograde units with distinct architecture.

Regarding Dynein-2: while IFT172 does not directly bind Dynein-2 (consistent with our MS data), the reviewer's intuition is correct that IFT172's binding partner influences Dynein-2 association. In anterograde trains, autoinhibited Dynein-2 binds a composite surface formed between adjacent IFT-B2 repeats. When IFT172 switches to IFT140 at the ciliary tip, the resulting train depolymerization destroys this composite binding site, releasing Dynein-2 from its cargo mode to function as an active retrograde motor. The IFT172 binding switch may thus indirectly acts as a structural checkpoint for Dynein-2 activation.

(2) The data provided regarding TGFbeta signalling effects in cells with heterozygous U-box-like domain deletions is interesting. While secondary effects of impaired ciliogenesis due to homozygous deletion of the U-box-like domain can cause difficulties to analysing cell signalling effects, it would still be interesting to check the effects of bi-allelic human IFT172 disease variants in this region as well (the human disease phenotype is recessive and human mutations are likely hypomorphic variants still allowing for ciliogenesis).

Also, while there may be secondary effects, it would still be interesting to check homozygous U-box deleted cells as an aggravated effect would further support the data from the het cells.

We agree that testing bi-allelic human disease variants would strengthen the physiological relevance of our findings. While generating knock-in RPE1 lines was beyond the scope of this revision, we have obtained preliminary data from patient-derived fibroblasts carrying bi-allelic IFT172 missense variants in the U-box region (NPH2161). TGF-β1 stimulation time courses in these fibroblasts show altered p-SMAD2 kinetics compared to control fibroblasts, consistent with the phenotype observed in our heterozygous U-box deleted RPE1 cells (see Author response image 3).

**Author response image 3.** 

While these results are preliminary and require further replication, they support the involvement of the IFT172 U-box domain in TGF-β signaling regulation in a disease-relevant context. Regarding homozygous U-box deleted cells, the severe reduction in IFT172 protein levels and ciliogenesis defects (Fig. 5B,D) make it difficult to separate U-box-specific effects from secondary consequences of impaired cilia formation, as the reviewer notes. We consider this an important direction for future studies using targeted point mutations rather than domain deletions.

(3) Figure 5 E-G: Overall, the effects upon TGFB1 addition are rather small compared to previously published data eg Clement et al Cell reports 2013 where one of the authors is the senior. Are RPE cells less responsive or do you have another theory? Did you check TGFB receptor levels to ensure the differences are not due to different levels of receptor expression? I feel it could be interesting to also check ciliary phopsho-SMAD localisation by IF. In Clement et al, loss of IFT88 results in reduced phospho-SMAD2 levels, do you have any theory why these opposite effects compared to the IFT172 loss of function could occur?

We thank the reviewer for this insightful comment. The Tg737orpk fibroblasts used in Clement et al. (2013), which harbor a hypomorphic mutation in IFT88, exhibit severely stunted cilia. This defect broadly disrupts cilium-dependent signaling pathways, including R-SMAD activation, and is therefore expected to produce more pronounced signaling phenotypes. In contrast, our study utilizes RPE-1 cells with structurally intact cilia, enabling us to investigate more specific alterations in ciliary signaling associated with IFT172 function rather than the global effects of cilia loss. Consequently, the more modest effects observed in our system are consistent with the less severe structural and functional perturbation. Both fibroblasts and RPE-1 cells are known to express TGF-β receptors and to respond robustly to TGF-β stimulation, making it unlikely that differences in receptor abundance alone account for the observed discrepancies. We also note that increasing evidence supports a role for the primary cilium in fine-tuning TGF-β signaling output by coordinating both canonical (R-SMAD-mediated) and non-canonical (e.g., AKT/ERK-mediated) pathways. Our data raise the possibility that loss of the IFT172 U-box domain, or reduced IFT172 levels, may differentially affect this balance, rather than simply attenuating signaling uniformly, as seen with more severe ciliary defects such as IFT88 disruption in Tg737orpk cells. We agree that the current dataset does not fully resolve the underlying mechanism. We therefore consider it an important direction for future work to examine, in greater detail, the localization and phosphorylation status of key canonical and non-canonical signaling components in context of the primary cilium by IF analyses.

(4) In the summary conclusion at the end of the discussions, the authors propose that IFT72 could directly influence the fate of ubiquitinated TGFB receptors. Do you have any data supporting the theory that TGFB ubiquitination is influenced by IFT172 ?

We acknowledge that our current data are insufficient to establish a direct link between IFT172-dependent ubiquitination events and TGF-β receptor regulation. Accordingly, we have revised the Discussion (page 19) to remove our previous hypothesis proposing a role for IFT172 in modulating TGF-β receptor ubiquitination.

While our experiments demonstrate that the U-box region is required for both IFT172 stability and proper TGF-β signaling, we agree that establishing a direct mechanistic connection between ubiquitin-related activity of IFT172 and signaling outcomes would require additional approaches such as targeted point mutations that selectively disrupt ubiquitin-binding or conjugation functions.

Furthermore, we note that our current data do not allow us to distinguish whether the observed signaling phenotypes arise specifically from the loss of ubiquitin-related functions of the U-box domain or from reduced levels of functional IFT172 protein in the heterozygous U-box–deleted cells.

(5) Wording:

Abstract

"IFT72..is associated with several disease variants causing ciliopathies". I would change this to "..and several disease-causing IFT172 variants have been identified in ciliopathy patients".

Corrected.

Introduction

"Another cohort of patients with milder ciliopathy resembling BBS also presented with ...". I would reword this to "Another cohort of patients with phenotypically slightly different ciliopathy features resembling BBS also presented with ...". It`s not necessarily less severe (they may die of cardiovascular complications in their early thirties for example due to metabolic syndrome, they are intellectually impaired, become blind...), but rather different.

Changed according to the reviewer’s recommendations.

**Reviewer #3 (Recommendations for the authors):**

(1) Recommended modifications:

(a) The RPE lines generated should be described better, i.e. sequencing information should be provided, or some kind of evidence that the lines are what they are supposed to be.

As also noted above, we acknowledge that the characterization presented for the RPE cell lines was insufficient in the initial version of the manuscript. In the revised version, we have addressed this limitation by including detailed sequencing analyses to validate the modifications introduced. Specifically, we provide sequencing data confirming both the integration of the GFP tag and the successful deletion of the U-box domain in all four engineered RPE cell lines. These data verify the integrity of the edited loci and exclude the presence of unintended insertions or deletions at the targeted regions. The corresponding results are presented in Figures S6 and S7 of the revised manuscript, thereby strengthening the validation of the cellular models used in this study.

(b) It would be more convincing if more than one clone of the RPE lines were presented, as this could rule out possible clonal effects.

We acknowledge that only a single clone was characterized for each of the four genotypes (IFT172-FL homozygous, IFT172-FL heterozygous, IFT172∆U-box homozygous, IFT172∆U-box heterozygous), and we agree that independent clones would provide stronger protection against clonal artifacts. Generating and validating additional clones was not feasible within the scope of this revision. However, several features of our data mitigate this concern. First, the phenotypes scale with allele dosage: the homozygous ∆U-box line shows the strongest reduction in IFT172 protein level, ciliation, and cilium length, while the heterozygous line shows intermediate defects (Fig. 5B, D and Fig. S8). A clonal off-target effect would not be expected to produce this dose-dependent pattern across two independently isolated lines. Second, the reduced steady-state IFT172 level in the ∆U-box lines (Fig. S8) is consistent with our in vitro observation that the U-box/TPR interface is required for protein stability, providing an independent biochemical rationale for the cellular phenotype. Third, Sanger sequencing of all four lines confirmed precise in-frame integration with no indels at the targeted locus (Figs. S6, S7). We have added a sentence to the Discussion (p. 20) acknowledging that confirmation in additional independent clones remains an important goal for follow-up work.

(c) Figure 5C: distribution of the GFP-tagged IFT172∆U-box protein could be quantified to support the statement.

In the revised version of the manuscript, we have included additional quantification of GFP fluorescence across all four cell lines to support our conclusions regarding IFT172 ciliary localization. The corresponding data for each cell line are presented in Figure S5C–F.

(d) The final sentences include quite bold statements about a general function of IFT172 in signal regulation. Yet, the evidence is the weakest part of the work. It is only shown in (i) one cell line, (ii) in one cell clone that is not extensively characterized, and (iii) for one signaling pathway that is not the best-studied cilia signaling pathway. Therefore, I recommend a more moderate statement.

Abstract last sentence has now been toned down and reads: Our findings suggest that IFT172, beyond its structural role in bridging IFT-A and IFT-B complexes within IFT trains, harbors a conserved U-box-like domain with potential involvement in ciliary ubiquitination processes and signaling, providing new insights into the molecular mechanisms underlying IFT172-related ciliopathies.

(e) The order of the figures is not followed in the main text, which is distracting.

The order of figures is now consecutive in the revised manuscript.

(2) Questions and comments to consider:

(a) It is unclear why tetra-ubiquitin chains have been used.

We thank the reviewer for this question. Recent evidence suggests that ubiquitin chains, rather than monomeric ubiquitin, act as sorting and signaling cues at the primary cilium (Shinde et al., 2020). To probe the ubiquitin-binding activity of IFT172, we therefore used a tetrameric ubiquitin chain as a model substrate, which better reflects the multivalent nature and binding avidity expected for physiological polyubiquitin signals than a ubiquitin monomer. Specifically, we used a recombinantly expressed linear (Met1-linked) tetra-ubiquitin chain, generated as a genetically encoded fusion. Linear ubiquitin chains are well-established non-degradative signaling chains recognized by a dedicated class of ubiquitin-binding domains, making them a suitable probe for detecting ubiquitin-binding activity outside the canonical proteasomal pathway. In addition, monomeric ubiquitin (~8 kDa) is poorly retained during membrane transfer in Western blotting, which further precluded its reliable use as a probe in our pull-down assays. Together, these considerations motivated the use of tetrameric ubiquitin as a biologically and technically appropriate substrate for assessing IFT172's ubiquitin-binding activity.

(b) Figure 4D: described in the text as "pulldown tetraubiquitin at comparable levels", which is not obvious from the figure presented, it appears reduced by at least 30%.

We thank the reviewer for this observation. As described on page 10 of the manuscript and evident from Figure 4D, the purified GST–HsIFT172C3 construct underwent substantial proteolytic cleavage during purification. This degradation limited our ability to include amounts of intact GST–HsIFT172C3 comparable to those of the full-length GST–HsIFT172C2 construct in the pull-down assays. Importantly, when accounting for the reduced proportion of full-length GST–HsIFT172C3 present in the assay, the observed differences in tetra-ubiquitin pull-down efficiency between the two constructs are expected to be comparable. This is supported by the Coomassie staining shown in Figure 4D, which reflects the relative abundance of the intact protein species used in the experiment.

(c) With the proposed model, why would the fla11 mutant only affect retrograde IFT?

We have revised our manuscript in page 16 of the discussion section providing a plausible explanation of why only retrograde IFT is affected in the fla11 mutant.

(3) Minor copy-editing:

(a) Page 3, first paragraph: led : = leads.

(b) Kinesin-2 and Dynein-2 should be hyphenated.

(c) Page 4: wwp1 should be WWP1.

(d) Bonafide should be italicized: bona fide.

(e) Some abbreviations appear uncommon and therefore somewhat distracting: TGFB instead of TGF-beta, Cr in instances where specifically referred to the organism.

(f) Unprecise lab jargon: "very C-terminal".

(g) Lab jargon: "purified a C-terminal construct".

(h) Lab jargon: "pull-downs".

(i) Page 8: "DALI" only abbreviated.

(j) Page 9: "Appearance ... were observed" should be "was".

(k) Page 11: "I688" should be "I1688".

(l) Page 12: "PDs" unclear.

These minor points have been corrected.

We have revised the text and figures to ensure using the widely accepted nomenclature, using TGF-β to refer to the signaling pathway and TGF-β1 specifically when referring to the ligand.

We further revised the text to reflect the use “*Chlamydomonas reinhardtii*” in instances when referring to the organism and “Cr” when referring to the protein.

We have removed the informal phrases "very C-terminal" and "purified a C-terminal construct" from the revised manuscript. We have retained the term "pull-down," as this is well-established and widely used terminology in the biochemistry literature to describe the affinity-based co-isolation assays used here. PD has been replaced with pull-down.

The grammatical error on page 9 ("Appearance... “were observed") has been corrected to "was observed”.
